# Traditional Chinese Medicine, Ziyin-Mingmu Decoction, Regulates Cholesterol Metabolism, Oxidative Stress, Inflammation and Gut Microbiota in Age-related Macular Degeneration Models

**DOI:** 10.1007/s11095-025-03887-3

**Published:** 2025-06-26

**Authors:** Xing Li, Khalid S. Ibrahim, Michal R. Baran, Gabriel Mbuta Tchivelekete, Xinzhi Zhou, Yi Wu, James Reilly, Zhoujin Tan, Zhiming He, Xinhua Shu

**Affiliations:** 1 Pu Ai Medical School, Shaoyang University, Shaoyang, Hunan 422000 P.R. China; 2 Department of Biology, Faculty of Science, University of Zakho, Kurdistan Region, Iraq; 3 Department of Biological and Biomedical Sciences, Glasgow Caledonian University, Glasgow, G4 0BA Scotland UK; 4 Department of Marine Biology, Faculty of Natural Science, University of Namibe, Namibe, Angola; 5 School of Traditional Chinese Medicine, Hunan University of Chinese Medicine, Changsha, Hunan 410208 P.R. China; 6 Department of Vision Science, Glasgow Caledonian University, Glasgow, G4 0BA Scotland UK

**Keywords:** age-related macular degeneration, cholesterol, gut microbiota, inflammation, oxidative stress, ziyin-mingmu decoction

## Abstract

**Background:**

Age-related macular degeneration (AMD) is the commonest cause of retinal disorders in the aged population. Ziyin-Mingmu decoction (ZD) has been widely used to treat AMD patients over thousands of years, however the underlying functional mechanisms of ZD are largely elusive. In this study, we aim to elucidate the therapeutic mechanisms of ZD in AMD models.

**Methods:**

An *in vivo* AMD mouse model and an *in vitro* AMD model were established. Cholesterol level in mouse tissues was measured. Expression of antioxidant genes and proinflammatory cytokines in mouse tissues and in human retinal pigment epithelial (RPE) cells were detected using biochemical approaches. Gut microbiota community and functional pathways were analysed using bioinformatics approach. Compounds in ZD were identified using HPLC/MS.

**Results:**

High fat diet (HFD)-fed mice had significantly higher levels of cholesterol in the retina, RPE, liver and serum, and markedly decreased expression of cholesterol metabolism-associated genes in those tissues, compared to mice fed with normal diet. Similarly, expression of antioxidant and inflammation genes was dysregulated in HFD-fed mouse tissues. ZD treatment reversed these HFD-induced pathological effects. HFD also altered the composition of cecum bacterial communities and associated metabolic pathways, which returned to control levels by ZD. *In vitro* assays showed that H_2_O_2_ significantly increased oxidative stress and enhanced expression of proinflammatory cytokines. Co-treatment with ZD significantly counteracted these changes. HPLC/MS identified 105 compound in water extracted ZD and most are polyphenols.

**Conclusion:**

Our data suggests that protection of ZD against AMD is possibly through mitigating cholesterol level, oxidative stress and inflammation, and modulating gut microbiota by polyphenols.

**Supplementary Information:**

The online version contains supplementary material available at 10.1007/s11095-025-03887-3.

## Introduction

Age-related macular degeneration (AMD) is the predominant retinal disorder in the elderly, causing loss of central vision and blindness. It has been estimated that AMD will have affected approximately 196 million individuals in 2020 and that this will increase to 288 million by 2040 [1]. Early AMD is characterized by accumulation of extracellular deposits (termed drusen) and abnormal pigmentary changes; late AMD is classified into two forms: dry AMD, characterized by geographic atrophy, and wet AMD, characterized by neovascularization [2]. AMD is a complex retinal disease, associated with multiple risk factors. Age is most the predominant risk factor for AMD: the prevalence is estimated to be 1.4% at 70 years old, increasing to 5.6% at 80 years old and 20% at 90 years old [3]. Smoking remains the major modifiable risk factor: current smokers have three-times higher risk to develop AMD than that of non-smokers [4]. Genome wide association studies (GWAS) have suggested multiple genes associated with AMD pathogenesis, in which both complement factor h and ARMS2 are major risk factors [5]. Drugs targeting vascular endothelial growth factor (VEGF) have shown therapeutic effectiveness and are widely employed in the treatment of wet AMD [2]. Last year two drugs targeting the complement system were approved by the United States Food and Drug Administration to treat dry AMD; these can slow the disease progression but do not enhance visual function [6]. Anti-VEGF drugs or drugs targeting complement require delivery to the retina via intravitreal injection, a treatment that involves repeat injections and which has adverse complications, including ocular inflammation, ocular haemorrhage, increased intraocular pressure and retinal detachment [7]. Consequently, new alternative treatments need to be developed.

Cholesterol is a multiple functional lipid that is involved in cellular regulation and signal transduction, and which is associated with a wide range of disorders, such as atherosclerosis, obesity, diabetes and cancer [8]. Dysregulation of cholesterol metabolism and trafficking plays an important role in the development and progression of AMD [9]. Cholesterol is abnormally accumulated in the drusen, a hallmark of AMD, and accounts for at least 40% volume of hard drusen [10]. There is high production of reactive oxygen species (ROS) in the retina due to high oxygen consumption and continuous light exposure, which results in cholesterol oxidation to form oxysterols, including the predominant one, 7-ketocholesterol. The latter is enriched in the aged retinal pigment epithelium (RPE) and drusen, and is believed to cause inflammation, defective cell adhesion and migration, oxidative stress, endoplasmic reticulum stress, and cell death, all of which contribute to AMD pathogenesis [11]. Some of cholesterol-homeostasis-related genes, including ATP-binding cassette subfamily member 1 (ABCA1), apolipoprotein E (APOE), cholesteryl estertransfer protein (CETP) and hepatic lipase C (LIPC), are involved in the pathogenesis of AMD [5]. Intake of high dietary cholesterol increases the risk of developing AMD [12, 13]. Animals fed with high cholesterol diet present AMD pathological features [15]. All these studies implicate cholesterol in the pathogenesis of AMD and indicate that lowering cholesterol will therapeutically benefit AMD patients.

Traditional Chinese medicine (TCM) has been widely applied in treating both dry and wet AMD [16]. 196 TCM prescriptions, containing 168 Chinese herbs, have been reported for treating AMD. One of these formulae is Ziyin-Mingmu decoction (ZD), which is composed of 12 medicinal herbs. ZD has been shown to have protective effects in treating AMD patients in China [17, 18]. Recent network pharmacology analysis identified 66 targeting genes associated with the therapeutic effects of ZD against AMD; these targeting genes are predicted to be predominantly involved in oxidative stress, inflammation and lipid metabolism [20]. However, the underlying therapeutic mechanisms of ZD against AMD remain elusive.

In this project, we investigated the ZD-associated protective mechanisms in high-fat-diet (HFD) fed mice, a model of AMD. We treated HFD-fed mice with or without ZD and measured cholesterol in the retina, the liver and the serum, examined expression of antioxidant and proinflammatory genes in the retina and the liver, and analysed gut microbiota. We used human retinal pigment epithelial cells to evaluate antioxidant and anti-inflammation capacity of ZD. We also identified compounds in water extracted ZD using high-performance liquid chromatography-mass spectrometry (HPLC–MS).

## Materials and Methods

### ZD Preparation

The formula of ZD contains Nuzhenzi (*Ligustrum lucidum* W.T.Aiton) (fruit) 15 g, Baizhu (*Atractylodes macrocephala* Koidz) (root) 15 g, Danshen (*Salvia miltiorrhiza* Bunge) (root) 15 g, Gandihuang (*Rehmannia glutinosa* (Gaertn.) DC) (root) 15 g, Danggui (*Angelica sinensis* (Oliv.) Diels) (root) 12 g, Goji (*Lycium chinense* Mill) (fruit) 12 g, Xiakucao (*Prunella vulgaris* L) 10 g, Tusizi (*Cuscuta chinensis* Lam) 10 g, Chusizi (*Broussonetia monoica* Hance) 10 g, Wuweizi (*Schisandra chinensis* (Turcz.) Baill) (fruit) 6 g, Cheqianzi (*Plantago asiatical subsp. argentea*) 6 g and Gancao (*Glycyrrhiza uralensis* Fisch) (root) 10 g. The Latin names of Chinese herbal plants have been checked with http://www.worldfloraonline.org (accession date: 04/04/2025). The mixture of these medicinal herbs was soaked in 75 ml water and boiled under low heat for 30 min with a low simmer. The first batch of decoction was strained out from the herb mixture; the remaining herbal materials were re-boiled in 75 ml water for 30 min with a low simmer and the second batch of decoction was strained out. Two batches of decoction were combined and concentrated to 1.33 g/ml, which was kept at 4°C for the animal experiments.

### Animal Study Groups

All animal studies were approved by the Ethics Committee of Hunan University of Chinese Medicine (license number: SYXK (Xiang) 2019–0009). Four-weeks-old C57BL/6 male mice were randomly divided into three groups (10 animals/group): Group 1 was fed with a control diet for 13 weeks; Groups 2 and 3 were fed with high- fat- diet (HFD) containing 78.75% control diet to which was added corn oil (10%), lard (10%), cholesterol (1%) and sodium cholate (0.25%) for 13 weeks. Animals of Groups 1 and 2 were then treated daily with physiological saline via intra-gastric administration; animals of group 3 were intra-gastrically treated daily with ZD at a dose of 17.68 g/kg body weight. During the treatment, animal bodyweight was monitored weekly. After treatment of 30 days, mice were sacrificed and tissue samples were collected.

### 16S rRNA Sequencing and Bioinformatics Analysis

Bacterial DNA was extracted from mouse cecum samples using the QIAamp DNA Stool Mini Kit (QIAGEN, UK), according to the manufacturer’s instructions. The extracted DNA from individual samples was sent for sequencing of 16S rRNA genes by an Illumina Nova-Seq with 2 × 300 base paired-end reads. Universal primers of the 16S rRNA genes were targeted to amplify the hypervariable regions V3-V4.

QIIME2 was used for processing the sequences with the modification of setting the truncation length to 232 bp in DADA2 [21]; Silvia database was used for taxonomic classifiers (https://www.arb-silva.de/download/archive/qiime). The raw data for each sample were normalized, and the significant difference of relative abundance of bacterial taxa between groups was evaluated using Wilcoxon tests. The bacterial taxa were visualized by Stacked bar plots and Cluster heatmap using the SRplot tool (https://www.bioinformatics.com.cn). Additionally, α- and β- diversities of the bacterial community were evaluated and performed by different methods available in MicrobiomeAnalyst (https://www.microbiomeanalyst.com). In addition, functional metagenomic predictions were performed using the PICRUSt2 [22]. The raw abundance data for each MetaCyc pathways were also normalized to obtain relative abundance values and then analyzed using Statistical Analysis of Metagenomic Profiles (STAMP, version 2.1.3) with ANOVA (Welch's t-test) between groups. Considering *p* < 0.05 was used to determine statistical significance across all analyses conducted.

### Preparation of ZD extract and identification of compounds

ZD prepared from Sect."ZD Preparation"was concentrated in a rotary evaporator under vacuum to approximately 1/8th of the original volume; the supernatants were then transferred to a glass evaporating dish to be desiccated in a vacuum oven. After complete drying, the extract was pulverized to a fine powder in a blender. The powder was used for *in vitro* experiments. For identification of compounds, 2 mg of extracted ZD was dissolved in 1 ml of 18MΩ water. The samples were diluted with mass spectrometer (MS) grade Acetonitrile (Optima™ liquid chromatography (LC)/MS Grade from Fisher scientific) to a final concentration of 2 µg/ml. Two injections of each sample were carried out in both positive and negative ionisation modes. For high performance liquid chromatography (HPLC), a HALO 90 A RP-AMIDE 2 UM 2.1 X 150 M column was used in a Dionex ultimate 3000 column compartment at 30°C with a flow rate of 0.2 ml/min using the Dionex ultimate RS 3000 Pump. A gradient elution scheme was selected with solution A (Acetonitrile) and solution B (10 mM Ammonium formate, pH3.5 adjusted with formic acid in 18MΩ water). The column was purged initially with 99% solution B and 1% solution A for 5 min, further reduced to 70% of solution B and 30% of solution A during 17 min, the concentration of solution B continued to reduce to 1% and the concentration of solution A continued to increase to 99% during the next 13 min, and finally the concentration of solution B was increased to 99%, solution of A was decreased to 1% over the last 10 min.

Samples were run in negative ionisation mode (−4.5 kV) then run again in positive ionisation mode (3.5 kV). 10 ul of the sample was injected each run using a Dionex ultimate 3000 RS autosampler. The software used to control the Thermo Scientific Q-exactive Orbitrap Mass spectrometer is Tracefinder + Xcalibur. In both positive and negative modes, the settings are as follows: full MS, 1 microscan, resolution of 17,500, AGC target 1e6, maximum IT 200 ms and one scan range 80–1200 m/z. For the MS2 run, the settings included 1 microscan at 17,500 resolution, AGC target 2e5, maximum IT 50 ms and isolation window 1 m/z with a scan range of 200–2000 m/z. The nitrogen gas sheath was run at 45 arbitrary gas units and auxiliary gas at 10 arbitrary gas units with an auxiliary gas temperature of 300°C. The raw data was analysed with MZMINE 4.5.0 software using mzwizard with HPLC and Orbitrap-DDA programme (Thermo Fisher Scientific LC–MS software). For annotation of individual compounds, MASSBANK and GNPS NIH Natural products and GNPS Prestwick phytochem libraries were used. Retention time was cropped to 40 min, FWHM of 3 s used and all other settings left as default.

### Cell Viability

Human retinal epithelial cell line ARPE-19 (ATCC CRL-2302™, passage 2), which has been widely used for AMD study, was cultured in a 96-well plate (5 × 10^4^ cells per well) with DMEM-F12 medium supplemented with 10% (v/v) fetal bovine serum (FBS), 0.26% sodium bicarbonate and penicillin (50 μg/ml)/streptomycin (50 IU/ml) for 24 h, then treated with ZD extract dissolved in PBS for 24 h at various concentrations. The cell viability was assessed using MTT reagent (Cat. No. M6494, Thermo Fisher Scientific) according to the manufacturer’s guidance.

### Biochemical Assays

ARPE-19 cells were seeded in 96-well plate (5 × 10^4^ cells per well) and incubated for 24 h. The cells were treated with/without H_2_O_2_ (750 µM, a dose chosen on the basis of our previous publications) [23], or H_2_O_2_ (750 µM) + ZD (20 µg/ml, a choice of dose based on cell viability of ZD, described in Sect. 2.9) for 24 h. Production of reactive oxygen species (ROS) was detected using DCFH-DA (6-Carboxy-20,70- Dichlorofluorescin diacetate, Cat. No. 21884, Sigma-Aldrich) based on the manufacturer’s guidance. Catalase activity and levels of malondialdehyde (MDA) and glutathione in untreated and treated ARPE-19 cells were measured with a catalase activity kit (Cat. No. STA-341, Cell Biolabs), a MDA detection kit (Cat. No. STA-330, Cell Biolabs) and a GSH assay kit (Cat. No. STA-312, Cell Biolabs), respectively, according to the manufacturer’s protocols. The levels of secreted proinflammatory cytokines from untreated and treated ARPE-19 cells were measured using the Mini ABTS ELISA Development Kits (Cat. No. 900-M95 for IL-1β, 900-M16 for IL-6, 900-M18 for Il-8 and 900-K25 for TNFα) from PeproTech, following the manufacturer’s guidance.

Total cholesterol in mouse tissue samples was measured with Amplex Red Cholesterol Assay kit (Cat. No. A12216, Thermo Fisher Scientific), based on the manufacturer’s protocol. The levels of IL-1β, IL-6 and TNFα in mouse tissue samples were measured with the Mini ABTS ELISA Development Kits (Cat. No. 900-K47 for Il-β, 900-K50 for IL-6 and 900-K54 for TNFα) from PeproTech following the manufacturer’s guidance.

### Measurement of Gene Expression

RNAs were extracted from mouse tissues or ARPE-91 cells using TRIzol™ reagent (Cat. No. 15596026) based on the manufacturer’s protocol. cDNAs were synthesized using a SuperScript **III** reverse transcription kit (Cat. No. 18080093, Thermo Fisher Scientific) following the manufacturers’ protocol. The mRNA level of individual genes was detected with SYBR™ Green universal master mix (Cat. No. 4312704, Thermo Fisher Scientific), guided by the manufacturer’s protocol. The primer sequences of targeted genes are listed in Table S1.

### Data Analysis

Data from biochemical assays and from measurement of gene expression were analysed using the prism graphpad 10 software (www.graphpad.com) with one-way ANOVA, followed by an appropriate post hoc test. Data is displayed as mean ± SE and a *p* value below 0.05 was set as significance.

## Results

### ZD Treatment Decreased Cholesterol Level in the Retinal Pigment Epithelium (RPE), Retina, Liver and Serum of High-fat Diet Fed Mice

The HFD-fed mouse model is widely used to understand lipid-associated pathological mechanisms in AMD [24, 25]. We monitored animals’ bodyweight during the experimental period and found that animals fed with HFD had significantly higher bodyweight than that of animals fed with normal diet from days 1 till days 81 (Figure S1A). After thirty days’ treatment, ZD-treated animals had body weight similar to that of the untreated HFD group (Figure S1B), suggesting ZD had no effect on body weight.

**Fig. 1:**
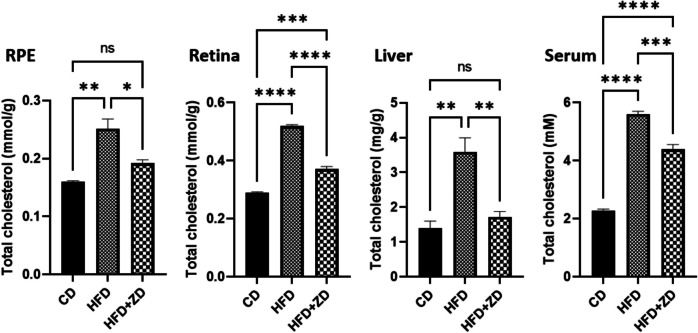
Effect of ZD treatment on cholesterol level in the RPE, retina, liver and serum. Data was analysed with one-way ANOVA, followed by Bonferroni multiple comparison test and is displayed as mean ± SE. CD, control diet; HFD, high-fat- diet; RPE, retinal pigment epithelial cells; ZD, Ziyin-Mingmu decoction. * *p* < 0.05, ** *p* < 0.01, *** *p* < 0.001, **** *p* < 0.0001; ns: no significance.

**Fig. 2:**
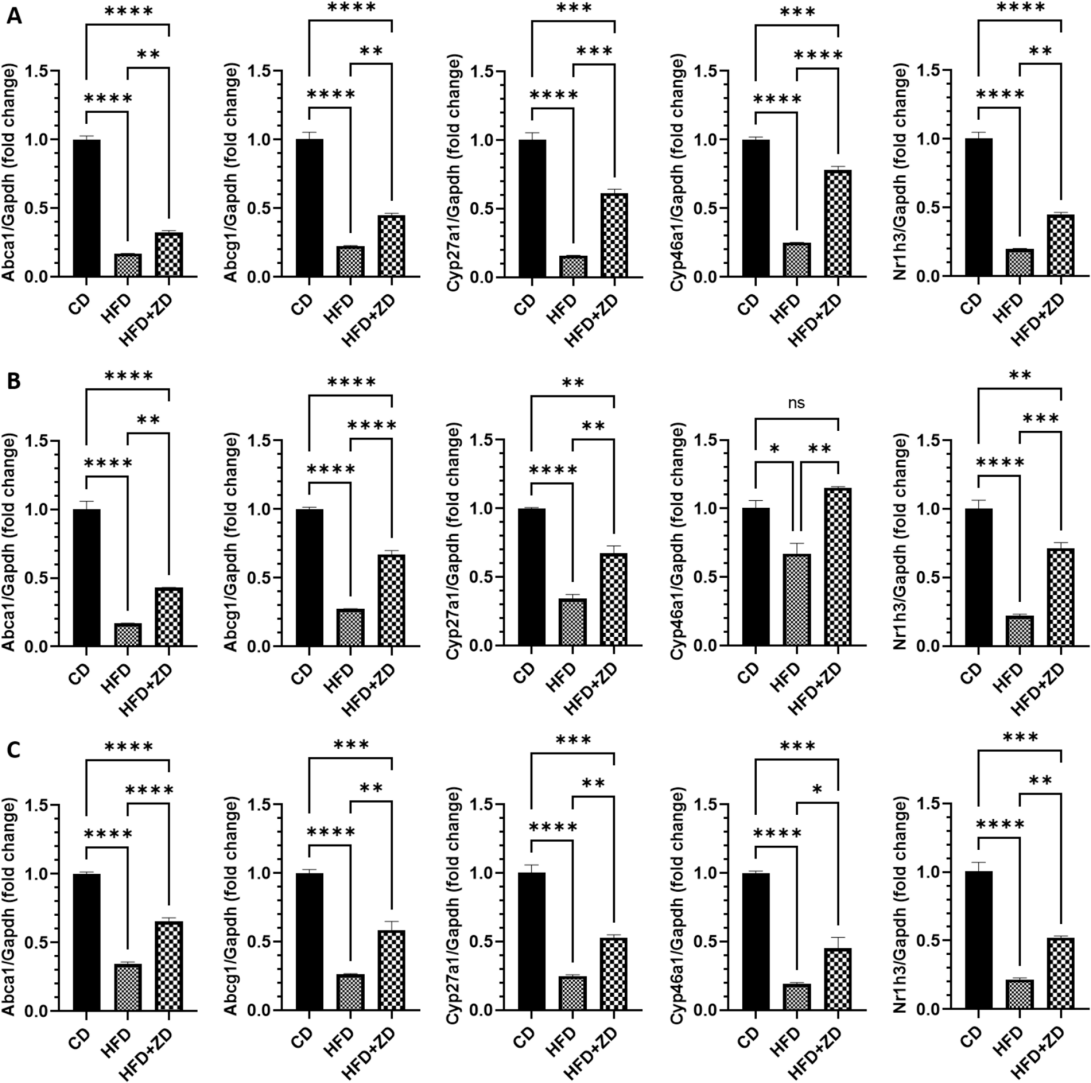
Effect of ZD treatment on expression of cholesterol trafficking and metabolism associated genes in the RPE (**A**), retina (**B**) and liver (**C**). Cycle threshold (CT) values of targeted genes were normalised to *Gapdh* gene then analyzed by 2^**−**ΔΔCt^ formula. The fold changes of individual genes were analysed with one-way ANOVA, followed by Bonferroni multiple comparison test and displayed as mean ± SE. CD, control diet; HFD, high-fat diet; RPE, retinal pigment epithelial cells; ZD, Ziyin-Mingmu decoction. * *p* < 0.05, ** *p* < 0.01, *** *p* < 0.001, **** *p* < 0.0001; ns: no significance.

When we measured the cholesterol level in the tissues of the three groups, we found that HFD-fed animals had a significant increase in cholesterol level in the RPE, retina, liver and serum, compared to those of animals fed with normal diet; ZD administration reversed HFD-induced effect on cholesterol level in the examined tissues (Fig. 1). We further examined expression of cholesterol metabolism and trafficking genes in the RPE, retina and liver. Expression of cholesterol transport genes (*Abca1* and *Abcg1*), cholesterol metabolism genes (*Cyp27a1*and *Cyp46a1*) and cholesterol metabolism regulating gene (*Nr1h3* encoding LXRα) was significantly decreased in HFD-fed mouse RPE, retina and liver, compared to those of animals fed with normal diet; ZD administrated mice had markedly increased expression of these genes, compared to that of the untreated HFD group (Fig. 2).

### ZD Treatment Regulated Expression of Antioxidant and Inflammation Genes

**Fig. 3:**
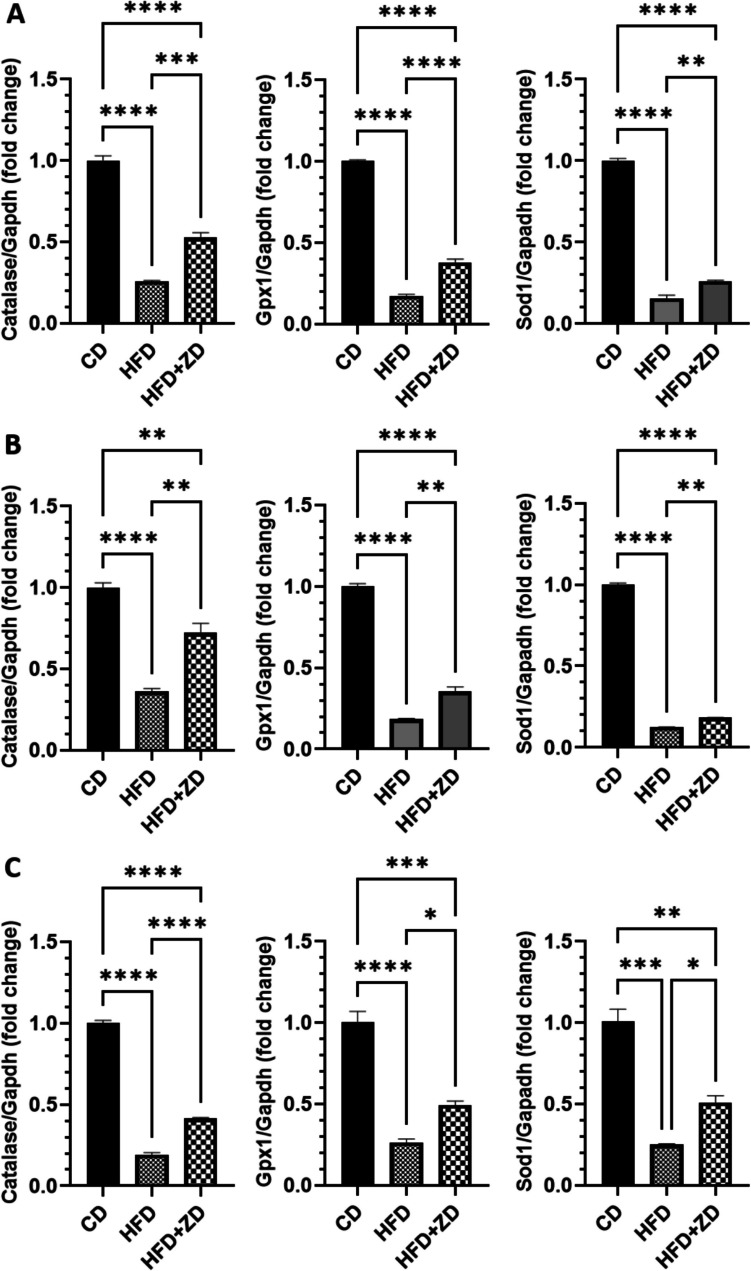
Effect of ZD treatment on expression of antioxidant genes, *catalase*, *glutathione peroxidase 1* (*Gpx1*) and *superoxide dismutase* (*Sod1*) in RPE (**A**), retinas (**B**) and liver (**C**). Cycle threshold (CT) values of targeted genes were normalised to *Gapdh* gene then analyzed by 2^**−**^.^ΔΔCt^ formula. The fold changes of individual genes were analysed with one-way ANOVA, followed by Bonferroni multiple comparison test and displayed as mean ± SE. CD, control diet; HFD, high-fat- diet; RPE, retinal pigment epithelial cells; ZD, Ziyin-Mingmu decoction. * *p* < 0.05, ** *p* < 0.01, *** *p* < 0.001, **** *p* < 0.0001.

HFD is held to induce oxidative stress and inflammation in mouse tissues [26]. Here we also confirmed that HFD feeding significantly downregulated expression of antioxidant genes, including catalase, *glutathione peroxidase 1* (*Gpx1*) and *superoxide dismutase* (*Sod1*), compared to that of animal fed with normal diet. However, ZD administration increased expression of the three genes, though the mRNA levels of the three genes were still lower than that of animals fed with normal diet (Fig. 3).

**Fig. 4:**
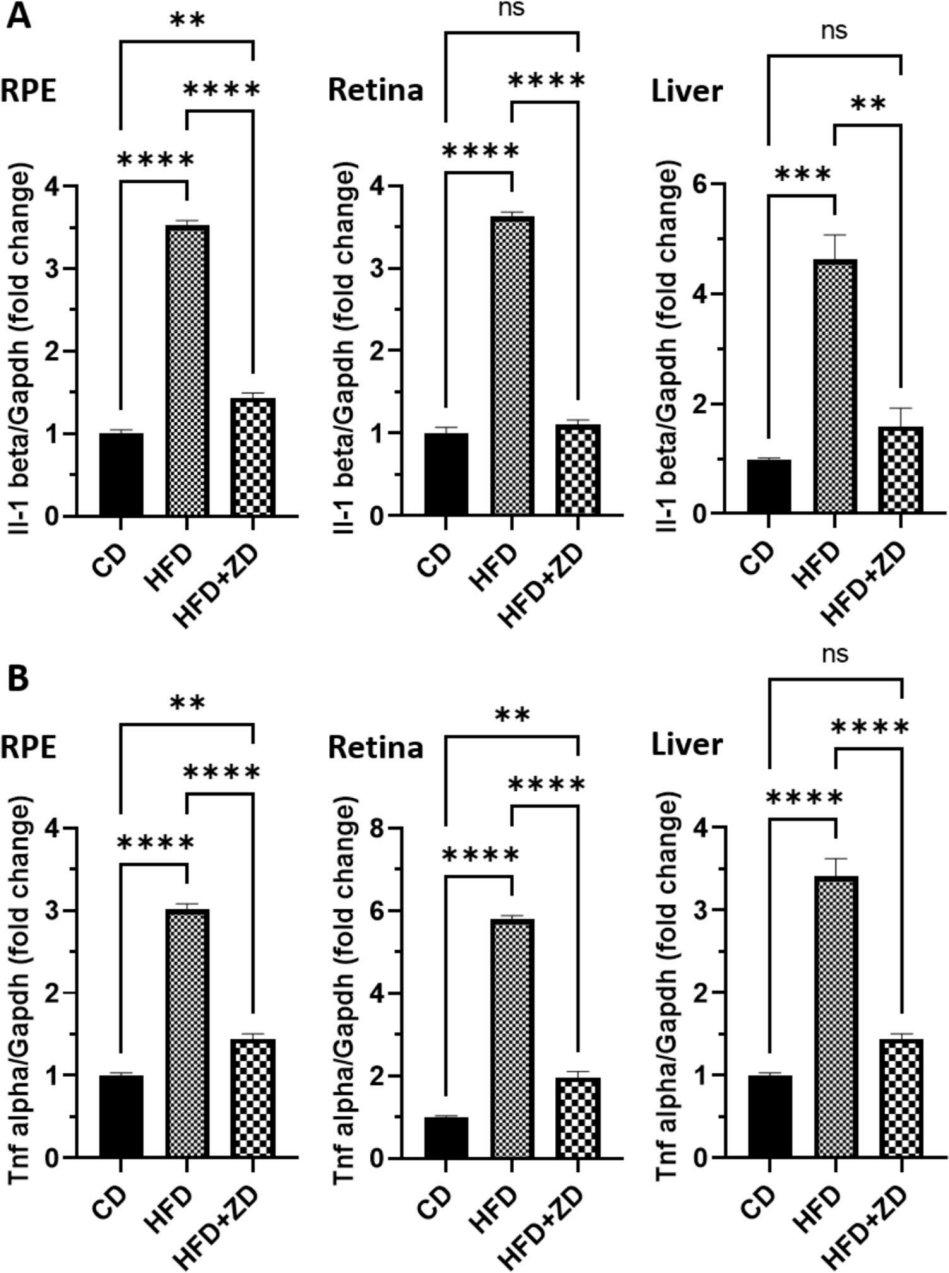
Effect of ZD treatment on proinflammatory genes, *Il-1β* (**A**) and *Tnfα* (**B**) in RPE, retina and liver. Cycle threshold (CT) values of targeted genes were normalised to *Gapdh* gene then analyzed by 2^**−**ΔΔCt^ formula. The fold changes of individual genes were analysed with one-way ANOVA, followed by Bonferroni multiple comparison test and displayed as mean ± SE. CD, control diet; HFD, high-fat diet; RPE, retinal pigment epithelial cells; ZD, Ziyin-Mingmu decoction. ** *p* < 0.01, *** *p* < 0.001, **** *p* < 0.0001; ns: no significance.

We also examined expression of proinflammatory cytokines in the three groups. HFD-fed animals had a marked increase in expression of *Il-1β* and *Tnfα* in RPE, retina and liver, compared to that of animals fed with normal diet. ZD administration resulted in a significant decrease in expression of *Il-1β* and *Tnfα* in those tissues, compared to HFD-fed animals (Fig. 4A, B). We further measured the levels of IL-1β, IL-6 and TNFα in the liver and serum of the three groups by ELISA. Both liver and serum samples of HFD-fed animals had significantly higher levels of those proinflammatory cytokines, compared to that of animals fed with normal diet (Fig. S2). ZD treatment significantly lowered the levels of IL-1β and TNFα in both liver and serum; the level of IL-6 in the liver was slightly decreased without significance, however serum IL-1β was significantly decreased, compared to that of HFD-fed animals (Fig. S2).

### ZD Treatment Altered the Composition, Abundance and Diversity of Gut Microbiota

Analysis of gut microbiome in the cecal samples from the three groups, six samples per group, was conducted using QIIME 2 Software. Initially, the total number of read sequences across all groups numbered 1,508,395. Quality control reads were overlapped and merged paired reads for each individual sample. Sequences not combined were filtered out and 1,190,963 non-chimeric merged reads were obtained (Table S2). These combined readings in each individual sample were performed for the final analysis.

**Fig. 5:**
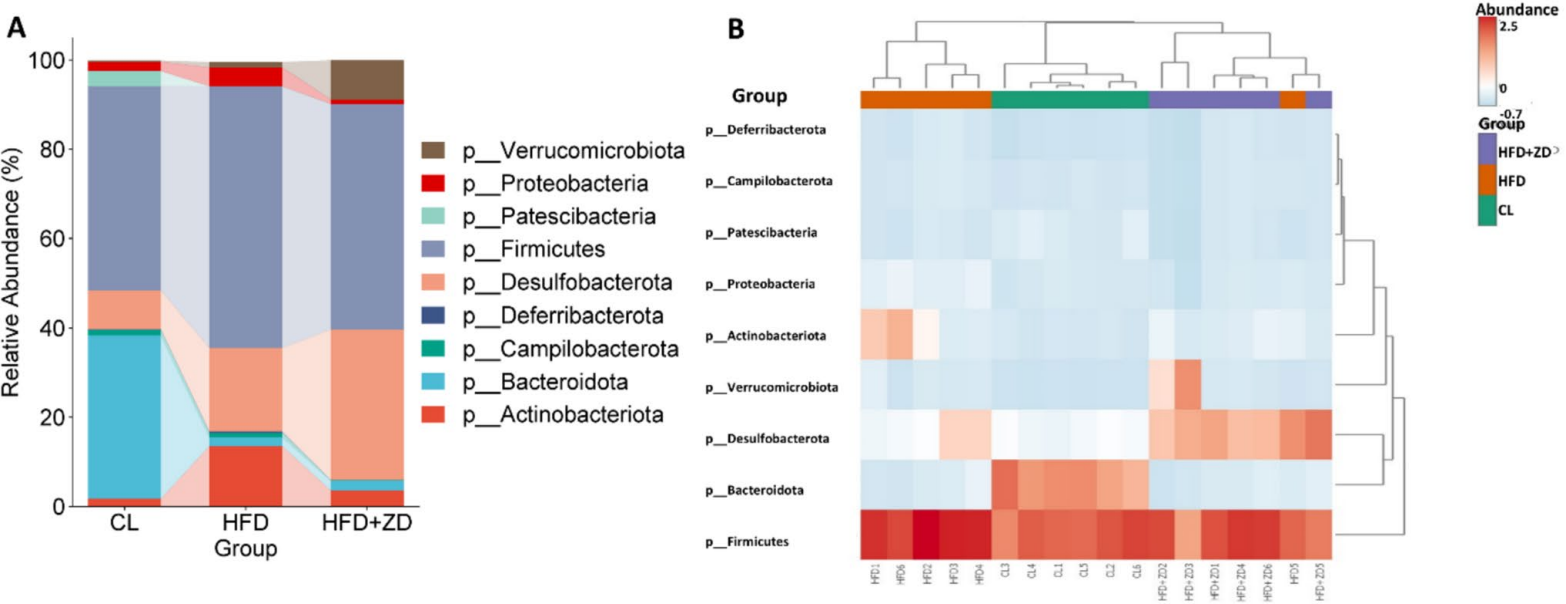
Difference of the phyla relative abundance difference (**A**) and Heatmap representation (**B**) in cecum samples of three experimental groups. The cecal content of control group (CL); High-fat- diet (HFD) group and High-fat- diet + ZD treatments (HFD + ZD) group.

A total of 373 taxa from nine phyla were identified, including *Actinobacteriota*, *Bacteroidota*, *Campilobacterota*, *Deferribacterota*, *Desulfobacterota*, *Firmicutes*, *Patescibacteria*, *Proteobacteria*, and *Verrucomicrobiota*, encompassing 128 families and 200 genera (Table S3). The composition and relative abundance of the gut microbiome varied significantly among the three animal groups. Figure 5 presents the gut microbiome at phylum level. *Firmicutes* was the predominant phylum across all three groups, followed by *Bacteroidota* in control group and *Desulfobacterota* in both HFD and HFD + ZD groups. The proportion of *Firmicutes* and *Actinobacteriota* were significantly higher in HFD group compared to control and HFD + ZD groups. In contrast, the abundance of *Desulfobacterota* was significantly elevated in both HFD and HFD + ZD compared to control group. In addition, the relative abundance of *Verrucomicrobiota* was significantly increased in ZD-treated group compared to both control and HFD groups (Fig. 5A). A heatmap analysis revealed distinct clustering patterns among the three groups, indicating a clear separation of their gut microbiome profiles (Fig. 5B). Notably, individual samples within HFD + ZD group clustered closely with those from control group, suggesting a shift in microbiome composition induced by ZD treatment.

**Fig. 6:**
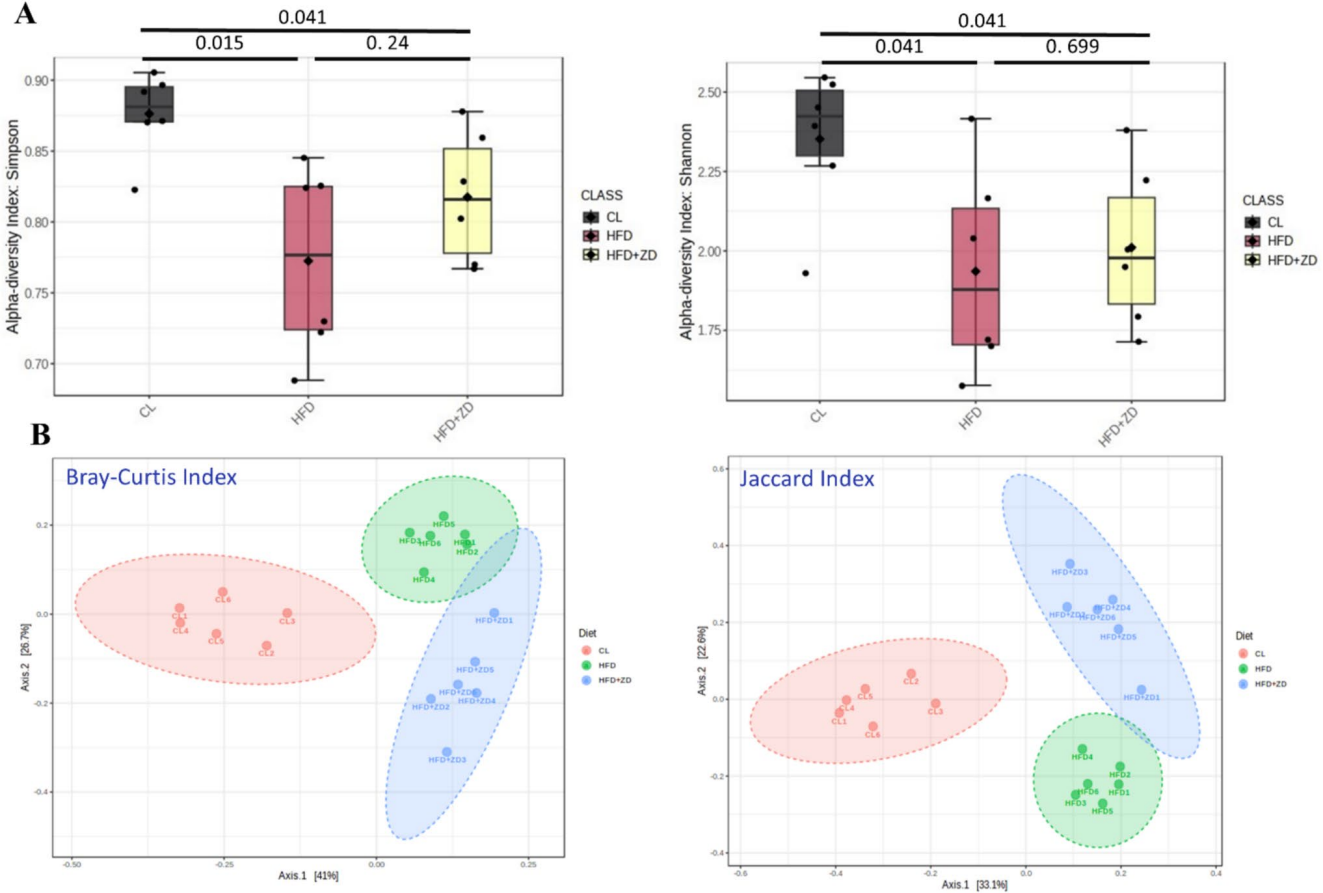
Bacterial community diversities, α-diversity (**A**) and β-diversity (**B**), among the three experimental groups. α -diversity: the Simpson and Shannon metrics revealed significant differences in the bacterial community diversity among three groups, with a p value < 0.05 considered statistically significant (a). β-diversity: Principal Coordinate Analysis (PCoA), generated using Bray–Curtis, and Jaccard Index, highlight distinct microbiome composition associated with each group. Control animals (red) display a distinct pattern, HFD animals (red) and HFD + ZD (green).

The diversity of the bacterial communities among the three groups was analyzed by metrics of α- and β-diversities. Indeed, α-diversity metrics such as the Simpson and Shannon metrics were significantly lower in HFD group (*p* < 0.05). Both Simpson and Shannon metrics of HFD + ZD group were significantly lower than that of control group (*p* < 0.05) and had no significant difference between HFD and HFD + ZD groups (*p* > 0.05), but there was a notable reversion of the two indices in the HFD + ZD group to resemble the levels of the control group (Fig. 6A). β-diversity analysis of the Bray–Curtis index and the Jaccard index showed clear clustering with distinct separation of gut microbial structures among the three groups and, to some extent, a shift of clustering in HFD + ZD closer to the control group (Fig. 6B).

### ZD Treatment Regulated Metabolic Pathways of Bacterial Communities

**Fig. 7:**
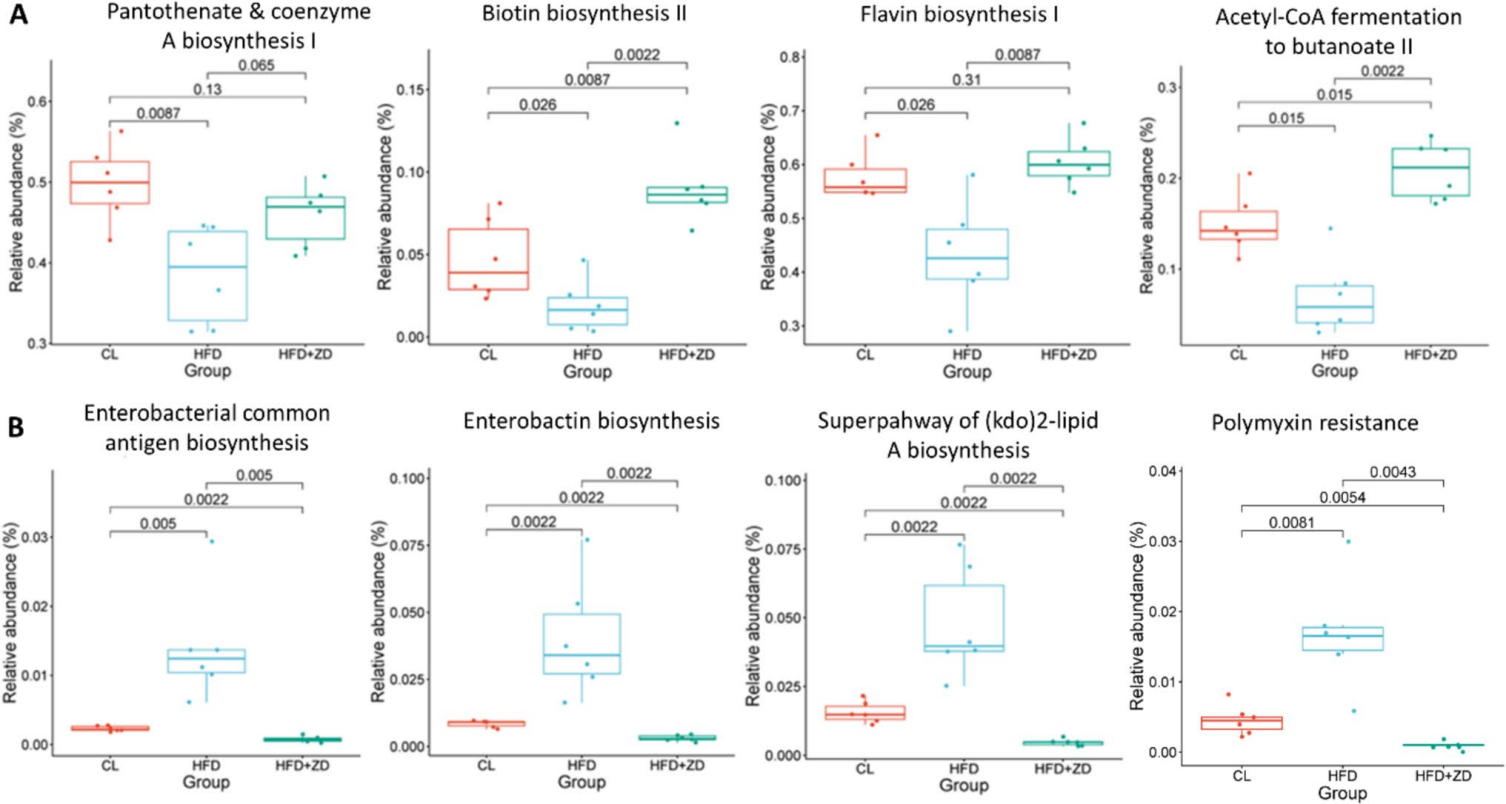
Significant differences in bacterial MetaCyc pathways of HFD compared with CL and HFD + ZD. (**A**) pathways that were significantly reduced and (**b**) pathways that were significantly increased in HFD. The relative abundance of MetaCyc pathways involved reducing cofactor-dependent and energy-support metabolisms (**A**) and enhancing the inflammation (**B**) in HFD compared with CL and HFD + ZD. A p value of < 0.05 was considered statistically significant.

The potential metabolic pathways of bacterial communities in the experimental groups were analysed using PICRUSt2. Pathways with functional relative abundance below 0.001 for all individual samples per group were excluded from the analysis and a total of 351 MetaCyc pathways were identified based on their relative abundance contributions. Among these pathways, 77 exhibited significant differences among the three groups (Table S4). When compared to that of control group, the abundance of 22 pathways was significantly increased while 11 pathways were significantly decreased in HFD group; compared to that of HFD group, the abundance of 36 pathways was significantly elevated in HFD + ZD group, while 40 pathways was significantly lowered in HFD + ZD group; compared to that of control group, the abundance of 35 pathways was significantly decreased, while 22 pathways were significantly increased in HFD + ZD group; notably 15 pathways were reversed to levels similar to those of the control group (Table S4). Further comparison of some pathways showed that there was a significant reduction of relative abundance of pantothenate and coenzyme A biosynthesis I, biotin biosynthesis II, flavin biosynthesis I (bacteria and plants) and acetyl-CoA fermentation to butanoate II in HFD; this was reversed by ZD treatment (Fig. 7A). HFD increased the abundance of enterobacterial common antigen biosynthesis, enterobactin biosynthesis, superpathway of (Kdo)2-lipid A biosynthesis and glyoxylate-bypass (glyoxylate cycle), while ZD treatment counteracted the effects (Fig. 7B).

### ZD Extract Inhibited Oxidative Stress and Inflammation in Retinal Pigment Epithelial Cells

**Fig. 8:**
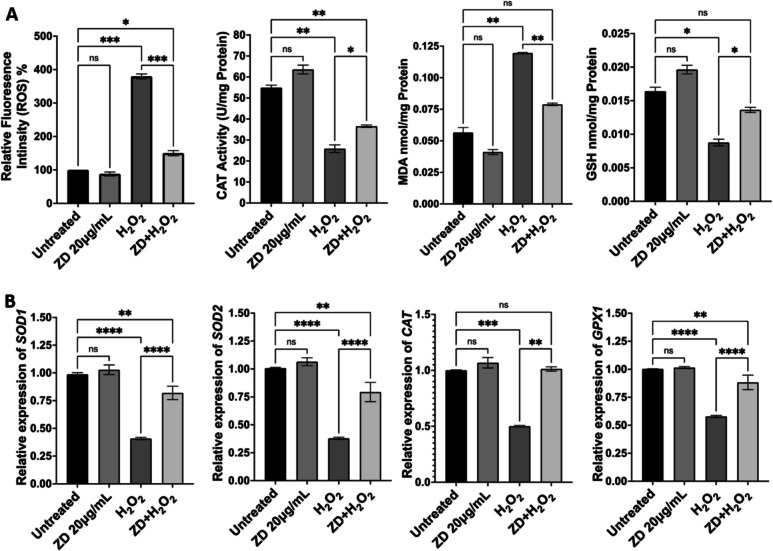
Effect of ZD treatment on antioxidant capacity in human retinal pigment epithelial cells. (**A**) Production of reactive oxygen species (ROS) and malondialdehyde (MDA), Catalase (CAT) activity, and glutathione level in untreated and treated cells were measured using commercial kits. (**B**) MRNA levels of antioxidant genes in untreated and treated cells measured by qRT-PCR. Data was analysed with one-way ANOVA, followed by Bonferroni multiple comparison test, and displayed as means ± SE. * *p* < 0.05, ** *p* < 0.01, *** *p* < 0.001, **** *p* < 0.0001; ns: no significance.

We also examined the capacity of ZD against oxidative stress and inflammation in human RPE (ARPE-19) cells. Initially, we assessed the potential toxicity of ZD to RPE cells using MTT assay and found that treatment of ZD at 5, 10, 20 or 40 µg/ml had similar cell viability to that of untreated cells. However, ZD at higher concentrations (80 and 100 µg/ml) showed toxic effect with a significant decrease in cell viability in treated cells compared to that of untreated cells (Figure S3). Subsequently, we used ZD at concentration of 20 µg/ml for further experiments. As we previously reported, H_2_O_2_ treatment resulted in significantly increased production of reactive oxygen species (ROS) and malondialdehyde (a biomarker for lipid peroxidation) and lowered catalase activity and glutathione level; ZD treatment reversed H_2_O_2_-induced effects (Fig. 8A). qRT-PCR data also showed that expression of antioxidant genes (superoxide dismutase1, superoxide dismutase 2, catalase and glutathione peroxidase 1) was markedly decreased in H_2_O_2_-treated cells compared to that of untreated cells, while co-treatment with ZD significantly increased expression of those genes, compared to that of cells treated H_2_O_2_ alone (Fig. 8B).

**Fig. 9:**
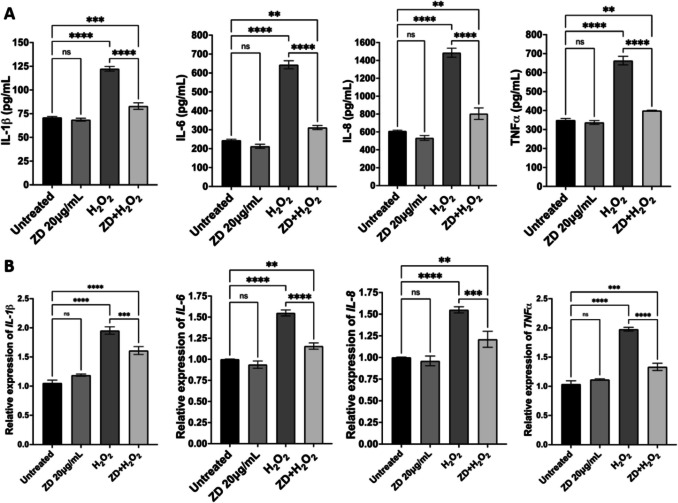
Effect of ZD treatment on expression of proinflammatory cytokines (**A**) Secreted IL-1β, IL-6, IL-8 and TNFα from untreated and treated cells measured by ELISA. (**B**) MRNA levels of *Il-1β*, *Il-6*, *Il-8* and *Tnfα* in untreated and treated cells examined by qRT-PCR. Data was analysed with one-way ANOVA, followed by Bonferroni multiple comparison test, and displayed as means ± SE. * *p* < 0.05, ** *p* < 0.01, *** *p* < 0.001, **** *p* < 0.0001; ns: no significance.

We also measured secreted proinflammatory cytokines in untreated and treated cells by ELISA. H_2_O_2_-treated cells had significantly higher levels of IL-1β, IL-6, IL-8 and TNFα, compared to that of untreated cells. ZD exposure resulted in a significant decrease in the levels of these cytokines, compared to that of cells only treated with H_2_O_2_ (Fig. 9A). Similarly, mRNA levels of proinflammatory cytokine genes in H_2_O_2_-treated cells were significantly higher than that of untreated cells; ZD treatment counteracted H_2_O_2_-induced effect on expression of proinflammatory cytokine genes (Fig. 9B).

### Identification of Main Compounds in Water-extracted ZD

**Fig. 10:**
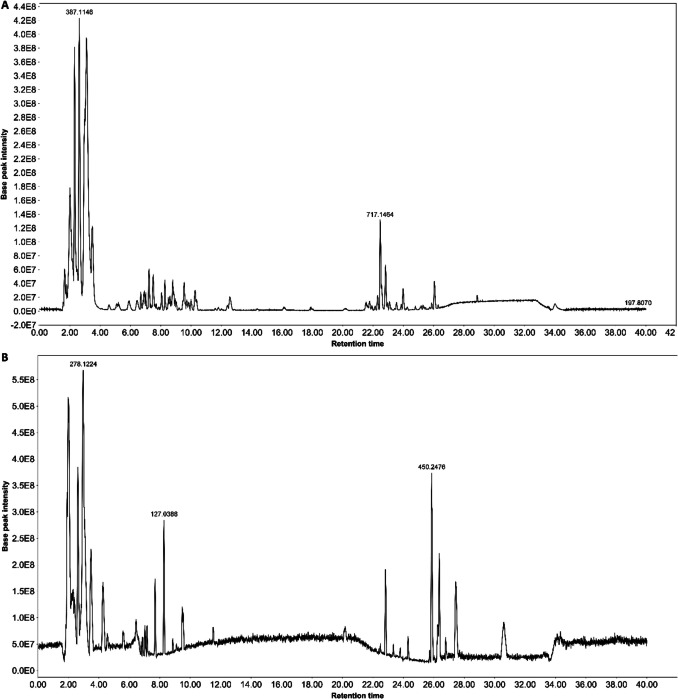
**(A)** Base peak chromatograms of ZD extract obtained in negative ionisation mode. (**B**) Base peak chromatograms of ZD extract obtained in positive ionisation mode.

We applied HPLC/MS to identify the main compounds in the water-extracted ZD and the total ion current chromatogram was analysed in both negative and positive ion modes (Fig. 10). Using the MZMINE software, 59 compounds were identified in negative ion mode and 60 compounds were identified in positive in positive ion mode, respectively, after alignment against the three databases (Tables I, II and S5). After excluding duplication in both negative and positive ion modes, 105 compounds were identified, including 22% flavonoids, 10% terprenes, 9% phenolics, 7% saccharides and carboxylic acids, 6% phospholipids, 5% coumarins, 4% fatty acids and pyridines, 3% glycosides and cinnamaldehydes, and 20% other compounds.

**Table I:** HPLC–MS Negative Ion Mode Analysis Identified Top 20 Most Abundant Compounds Based on Area Under Curve

m/z	RT	Area	Height	Ion identity	Spectral match	Chemical Classification
255.0663	23.98	4.50E + 07	4.80E + 08	[M-H]-	isoliquiritigenin	phenolic
133.0128	2.15	3.30E + 07	3.70E + 08		D-(+)-Malic acid	carboxylic acid
179.034	12.57	1.40E + 07	3.80E + 07	[M-H]-	3,4-dihydroxycinnamic acid	cinnamic acid
341.1086	2.65	9.40E + 06	8.40E + 07	[M-H]-	Sucrose	saccharide
267.0666	25.62	7.00E + 06	8.50E + 07		Formononetin	flavone
181.071	1.95	7.00E + 06	8.00E + 07		D-Sorbitol	sugar alcohol
329.233	25.74	6.20E + 06	3.60E + 07	[M-H]-	FA 18:1 + 3O	fatty acid
821.3979	26.3	5.10E + 06	5.60E + 07		Licoricesaponin H2	glycoside
353.0721	2.79	4.80E + 06	1.70E + 07		6:3 + 6O fatty acyl hexoside	Fatty acyl hexosides
503.1625	2.08	4.80E + 06	3.30E + 07	[M-H]-	Maltotriose	saccharide
163.0389	17.9	3.70E + 06	1.00E + 07		p-Coumaric acid	coumarin
209.0448	8.79	3.60E + 06	2.90E + 07		Hydroxyferulic acid	phenolic
359.0772	22.57	3.50E + 06	3.80E + 07	[M-H]-	Rosmarinic acid	coumarin
341.1085	2.1	3.50E + 06	3.30E + 07	[M-H]-	Palatinose	saccharide
595.1308	22.32	2.90E + 06	1.70E + 07	[M-H]-	Peltatoside	flavone
271.0615	24.65	2.70E + 06	3.20E + 07		Naringenin	flavone
417.1201	21.58	2.40E + 06	1.20E + 07	[M-H]-	Liquiritin	glycoside
463.0894	23.08	2.30E + 06	1.80E + 07	[M-H]-	Hyperoside	flavone
193.0499	19.99	1.90E + 06	3.40E + 06		trans-Ferulic acid	phenolic

**Table II:** HPLC–MS Positive Ion Mode Analysis Identified Top 20 Most Abundant Compounds Based on Area Under Curve

m/z	RT	Area	Height	Ion identity	Spectral match	Chemical Classification
433.2212	25.86	1.40E + 08	1.40E + 09	[M + H] +	Schizandrin	tannin
231.1378	27.22	6.20E + 07	8.90E + 08	[M + H] +	Dehydrocostus lactone	Sesquiterpene
279.2308	30.17	3.90E + 07	1.10E + 08		linolenic acid	fatty acid
760.5815	31.63	3.80E + 07	1.20E + 08		1-(9Z-octadecenoyl)−2-hexadecanoyl-sn-glycero-3-phosphocholine	phospholipid
257.0805	23.99	3.70E + 07	4.10E + 08	[M + H] +	isoliquiritigenin	phenolic
249.1484	26.32	2.80E + 07	3.00E + 08	[M + H] +	Atractylenolide III	Sesquiterpene
786.5971	32.25	2.20E + 07	6.50E + 07	[M + H] +	1,2-Dioleoyl-sn-glycero-3-phosphocholine	phospholipid
138.0548	2.12	1.50E + 07	8.90E + 07		Trigonelline	alkaloid
269.0803	25.63	1.40E + 07	1.70E + 08	[M + H] +	Formononetin	flavone
219.1741	27.63	1.20E + 07	1.60E + 08		Nootkatone	Sesquiterpene
179.0696	21.74	6.70E + 06	3.10E + 07		Coniferaldehyde	phenolic
823.4088	26.05	6.20E + 06	5.80E + 07	[M + H] +	Glycyrrhizic acid ammonium salt	terpene
586.5383	32.75	5.70E + 06	1.00E + 07	[M + NH4] +	1,2-Dipalmitoyl-rac-glycerol	lipid
193.0492	17.8	5.60E + 06	1.30E + 07	[M + H] +	Scopoletin	Coumarin
839.4034	25.88	5.60E + 06	5.70E + 07		Licoricesaponin G2	saponin
258.11	1.88	5.30E + 06	4.30E + 07		sn-Glycero-3-phosphocholine	phospholipid
782.564	31.48	3.60E + 06	1.20E + 07		1-hexadecanoyl-2-(9Z-octadecenoyl)-sn-glycero-3-phosphocholine	phospholipid
183.086	1.95	3.20E + 06	3.30E + 07		D-Sorbitol	sugar alcohol
126.0549	9	3.10E + 06	2.30E + 07		3-Hydroxypyridine-2-methanol	n/a

## Discussion

Although ZD has been shown to have therapeutic effects in AMD patients, the functional mechanisms remain elusive [16]. Here we demonstrated that treatment with ZD in HFD-fed mice, a model for AMD, significantly decreased cholesterol level in examined tissues, increased expression of cholesterol homeostasis-associated genes and antioxidant genes, downregulated expression of proinflammatory cytokines in the RPE, retina and liver, as well as regulated gut microbiota. We also showed the antioxidant and anti-inflammation capacity of ZD in human RPE cells.

HFD is strongly associated with higher prevalence of advanced AMD [27] and induces AMD-like pathology in mice [25]; cholesterol-enriched diet also induces AMD-like features in rabbits [15]. Cholesterol is a multiply functional lipid that accumulates in the drusen of AMD patients, possibly due to defects in cholesterol transport and metabolism [9]. Cholesterol in RPE cells can be metabolized by mitochondrial enzymes, CYP27A1 and CYP46A1 and form oxysterols, 27-hydroxycholesterol and 24-hydroxycholesterol, respectively, which upregulate expression of cholesterol trafficking-associated genes (mainly *NR1H3* encoding LXRα, *ABCA1* and *ABCG1*) and promote cholesterol reverse transport [9]. Loss of cholesterol metabolism enzyme (CYP27A1 or CYP46A1) caused dysregulation of retinal cholesterol homeostasis, build-up of cholesterol deposits underneath the RPE layer and abnormal retinal vascularization [28, 29]. RPE-specific double knockout of cholesterol transporters, *Abca1* and *Abcg1*, causes cholesteryl ester-enriched lipid accumulation in mouse RPE cells, abnormal morphology of RPE cells, increased inflammatory response and age-dependent degeneration of photoreceptors [30]. Here we also detected increased cholesterol level in the RPE and retina of HFD-fed mice, which was possibly caused by dysregulation of cholesterol metabolism and transport, as expression of both cholesterol metabolism and trafficking associated genes was significantly decreased in HFD-fed mouse RPE and retina. The effect of ZD on lowering cholesterol in the RPE and retina was possibly via enhancement of cholesterol metabolism and transport, as ZD upregulated expression of those associated genes. Excess cholesterol in peripheral tissues is removed by reverse transport to the liver, where cholesterol is stored as cholesteryl esters or converting into bile acids for excretion. Since HFD also caused increased cholesterol level in the liver and ZD reversed the HFD-induced effect, there was value in examining expression/activity of bile acid synthesis enzymes: expression of at least one of the enzymes, *Cyp27a1*, was downregulated in HFD-fed mouse liver and upregulated in ZD-treated HFD-fed mouse liver.

HFD is known to induce oxidative stress and inflammation and contribute to the pathogenesis of neurodegenerative disorders, including AMD [24, 31]. Previously we have shown that HFD significantly increased production of ROS and proinflammatory cytokines in mouse RPE. Here we further demonstrated that HFD downregulated expression of antioxidant genes and upregulated proinflammatory cytokines and showed that ZD treatment reversed the effect. There are complicated underlying mechanisms of HFD-causing oxidative stress and inflammation in the retina and RPE. One possible explanation here is that accumulated cholesterol is autoxidated to form 7-ketocholesterol and 7β-hydroxycholesterol, which can cause oxidative damage and inflammation [9]. Sterling *et al.* (2022) also demonstrated that HFD induced iron accumulation and oxidative stress via IL-1β-mediated regulation of iron importer/exporter expression [31]. Therefore, it would be worthwhile to examine whether counteraction of ZD against HFD-induced toxic effects is through decreasing formation of toxic oxysterols and/or modulating expression of iron importers/exporters. Additionally, our *in vitro* data showed that ZD protects human RPE cells from H_2_O_2_-induced oxidative stress and inflammation. The data provide solid evidence to support the benefit of ZD in treating AMD patients.

Human gut microbiota contains over 1000 bacterial species and serves multiple functions, for example food fermentation and uptake, synthesis of bioactive molecules, regulation of immunity and protection against pathogens. Dysbiosis of gut microbiota is associated with a wide range of diseases, such as metabolic and neurodegenerative disorders [32]. Germ free (GF) mice showed smaller size of laser-induced choroidal neovascularization (CNV) and decreased microglial activation around CNV, compared to that of specific pathogen-free (SPF) mice; SPF mice with CNV also had significantly higher expression of genes associated with inflammation and angiogenesis in RPE/choroid, compared to that of SF mice with CNV, suggesting that gut microbiota mediates AMD pathogenesis [33]. A few clinical studies showed that AMD patients had changes in bacterial composition at taxonomical levels [34, 35]. For example, Zinkernagel *et al.* reported wet AMD patients had elevated abundance of genera *Anaerotruncus* and *Oscillibacter*, with increased *Firmicutes*/*Bacteroidetes* ratio; metagenomic analysis demonstrated that pathways involved in arginine biosynthesis, glutamate degradation and L-alanine fermentation were enriched in wet AMD patients [34]. HFD is a risk factor for AMD and associated with development and progression of AMD and is also known to modulate gut microbiota, suggesting a link between diet and AMD [27]. Early studies showed that HFD-induced dysbiosis was associated with severe choroidal neovascularization in mice [36]. Here we also found significant changes in abundance of some phyla among the three animal groups: the Firmicutes/Bacteroidetes ratio was markedly increased in HFD mice and decreased in ZD-treated animals. We also found significant changes in bacterial metabolic pathways induced by HFD and partially reversed by ZD treatment. In term of individual decreased pathways in HFD mice, some are proposed to be associated with lipid metabolism, oxidative stress and inflammation. These pathways include biosynthesis of Coenzyme A (CoA), biotin and flavin, and acetyl-CoA fermentation to butanoate II, each of which was reversed by ZD treatment. Most gut bacteria can synthesize pantothenate (vitamin B5) from L-aspartate and a-ketoisovalerate and CoA is synthesized from pantothenate via five reactions [37]. CoA is a multiple functional molecule, involving in a wide range of metabolic and signalling pathways, such as fatty acid oxidation. Defects in CoA synthesis cause neurodegenerative disorders, including retinal degeneration, in humans and rodents [38]. Dysregulation of CoA-dependent metabolic pathways causes chronic disorders including obesity, diabetes, cardiovascular and inflammatory diseases, and cancer. CoA can modulate inflammation via regulating metabolic function of immune cells [39]. Biotin is a cofactor for carboxylases, which function in gluconeogenesis, fatty acid synthesis, and amino acid catabolism; biotin also plays an important role in regulation of gene expression. Biotin-deficiency has been linked to different types of metabolic and neurological disorders [40]. Similarly, flavins function as a cofactor for a wide range of enzymes, involving multiple metabolic pathways such as free radical scavenging and lipid metabolism. Flavin deficiency is associated with diabetic retinopathy, possibly via impairment of antioxidant capacity and fatty acid β-oxidation as flavins are used as cofactors by glutathione reductase and co-A dehydrogenases [41]. Butyrate (butanoate) is produced by bacteria and plays an important role in regulating metabolic function and suppressing inflammation in the host. Butyrate has been shown to increase retinal function, inhibit retinal inflammation, and alleviate retinal pathology in retinal degeneration models [32]. Conversely, the synthetic levels of enterobacterial common antigen, enterobactin and superpathway of (kdo)2-lipid A and the polymyxin resistance are significantly higher in HFD animals, compared to that of animals fed with normal diet, and the levels of those pathways were markedly decreased even lower than control levels after ZD treatment. These bacterial metabolites can induce host inflammation; for example, enterobacterial common antigen can link to lipopolysaccharide (LPS), which can induce inflammation in the host [42]. Both enterobactins and polymixins can induce inflammation, causing damage to the host [43, 44], so it would be useful to examine the levels of these beneficial and toxic metabolites in related organs, such as gut, liver, serum, retina and RPE.

ZD consists of 12 herbal medicines with multiple functional ingredients and is believed to have complex effects on the gut microbiota and host. Individual herbal medicines of ZD have been shown to alleviate pathological features in disease rodent models via modulation of host microbiota. For example, wolfberry polysaccharides mitigate high-fat and high-fructose induced cognitive deficiency and gut dysbiosis in mice [45]; *Atractylodes macrocephala* polysaccharides ameliorate dexran sulfate sodium induced intestinal injury by enhancing growth of beneficial bacteria and suppressing host inflammation in mice [46]. We identified 105 compounds in water extracted ZD, of which most are polyphenols, including flavonoids (22%), phenolics (9%) and coumarins (3%). Polyphenols have been shown against oxidative, inflammation and angiogenesis in *in vitro* and *in vivo* AMD models [47]. Recently we also showed flavonoids (quercetin, luteolin and naringenin, also identified from ZD extract in current study) suppressed H_2_O_2_-induced oxidative damage and inflammation in human RPE cells [48]. Polyphenols have also been shown regulation of cholesterol metabolism, modulation of gut microbiota and protection against cholesterol-associated disorders [49, 50]. Future investigation will validate the effects of individual identified polyphenols on AMD-associated pathology.

## Conclusion

**Fig. 11:**
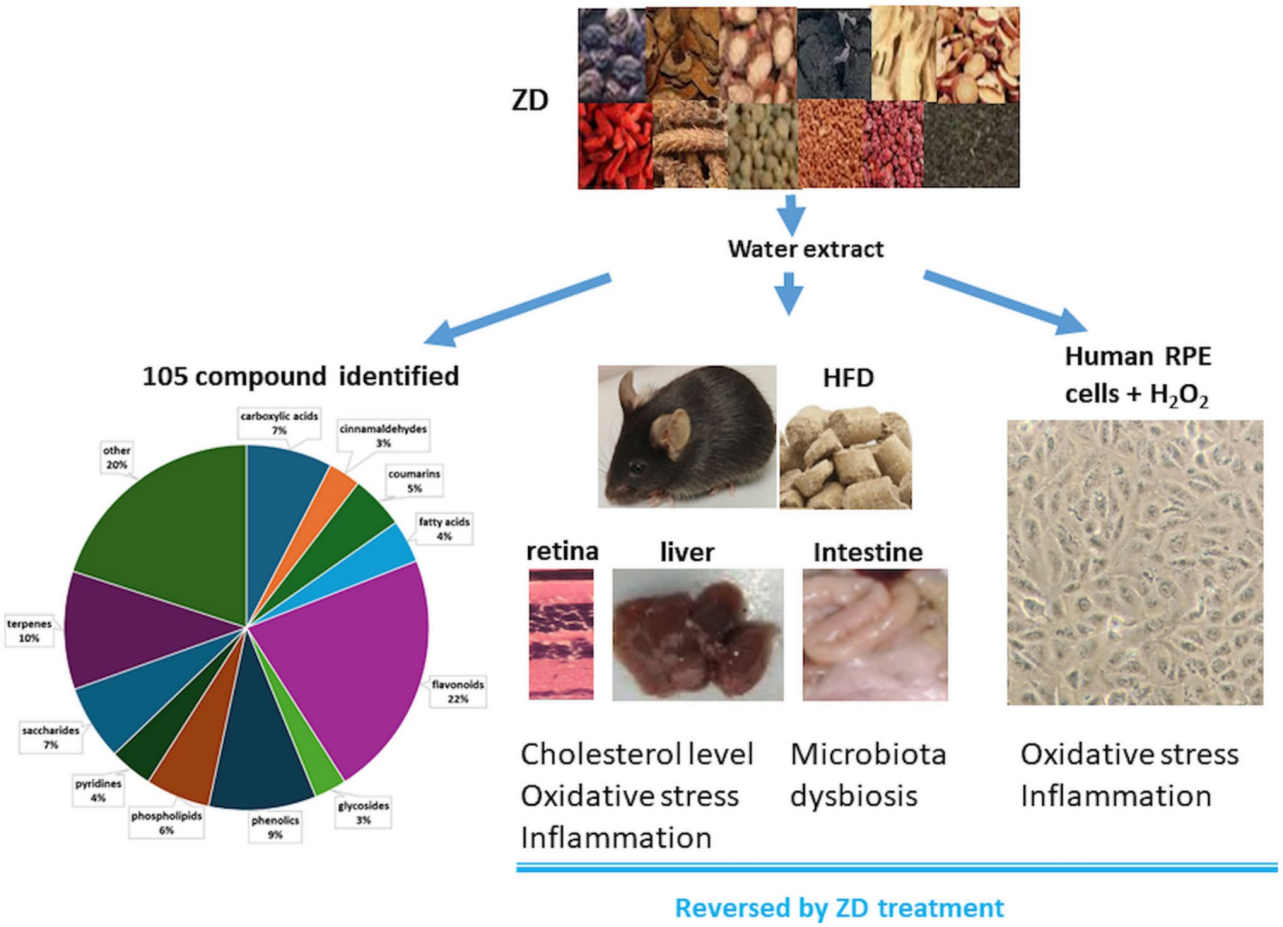
The underlying mechanism of Ziyin-Mingmu decoction (ZD) against age related macular degeneration (AMD) is possibly via regulating cholesterol metabolism, suppressing oxidative stress and inflammation in the retina and liver, and modulating gut microbiota by the functional compounds, mainly polyphenols, identified in the water extract of ZD.

The present study investigated the protective mechanisms of ZD against AMD in HFD-fed mice. HFD was shown to increased cholesterol accumulation in the RPE, retina, liver and serum, and was associated with oxidative stress and inflammation; HFD also exacerbated gut dysbiosis. ZD treatment counteracted HFD-induced effects. We propose that dysregulation of lipid metabolism, decreased antioxidant capacity and elevated inflammation in the retina, RPE and liver are possibly associated with changes occurring in the gut bacterial metabolic pathways. Polyphenols are predominant compounds identified in ZD and mitigate HFD-induced toxic effects via regulating cholesterol metabolism, inhibiting oxidative stress and inflammation, and modulating gut microbiota (Fig. 11).

## Supplementary Information

Below is the link to the electronic supplementary material.

Supplementary file1 (DOCX 686 KB)

Supplementary file2 (XLSX 35 KB)
