# Supplementary material for: Traditional Chinese Medicine, Ziyin-Mingmu Decoction, Regulates Cholesterol Metabolism, Oxidative Stress, Inflammation and Gut Microbiota in Age-related Macular Degeneration Models
Source: Pharm Res. 2025 Jun 26;42(7):1101–18. doi: 10.1007/s11095-025-03887-3 (PMC12304070; doi:10.1007/s11095-025-03887-3)
Supplement: Supplementary file 1 — Supplementary file1 (DOCX 686 KB) [file 11095_2025_3887_MOESM1_ESM.docx]

**Supplementary data**

**
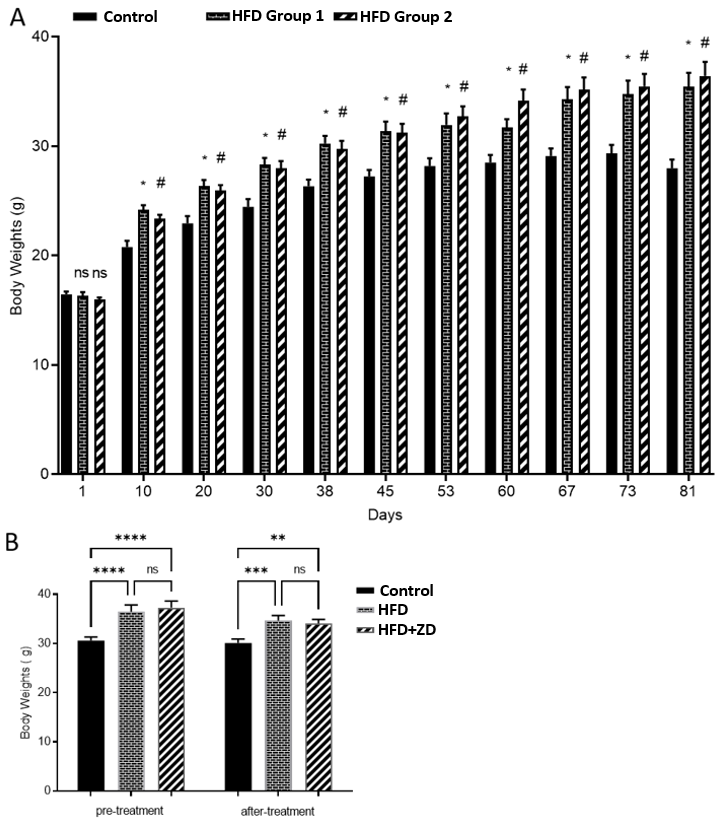
**

**Figure S1 B**odyweight of three groups. (A) Body weight of animals during feeding with normal diet or high-fat diet (HFD). * HFD group 1 compare to control, *p*<0.001; # HFD group 2 compared to control, *p*<0.001. (B) Bodyweight of animas pre-treatment and after-treatment. ** *p*<0.01, *** *p*<0.001, **** *p*<0.0001; ns: no significance.

**
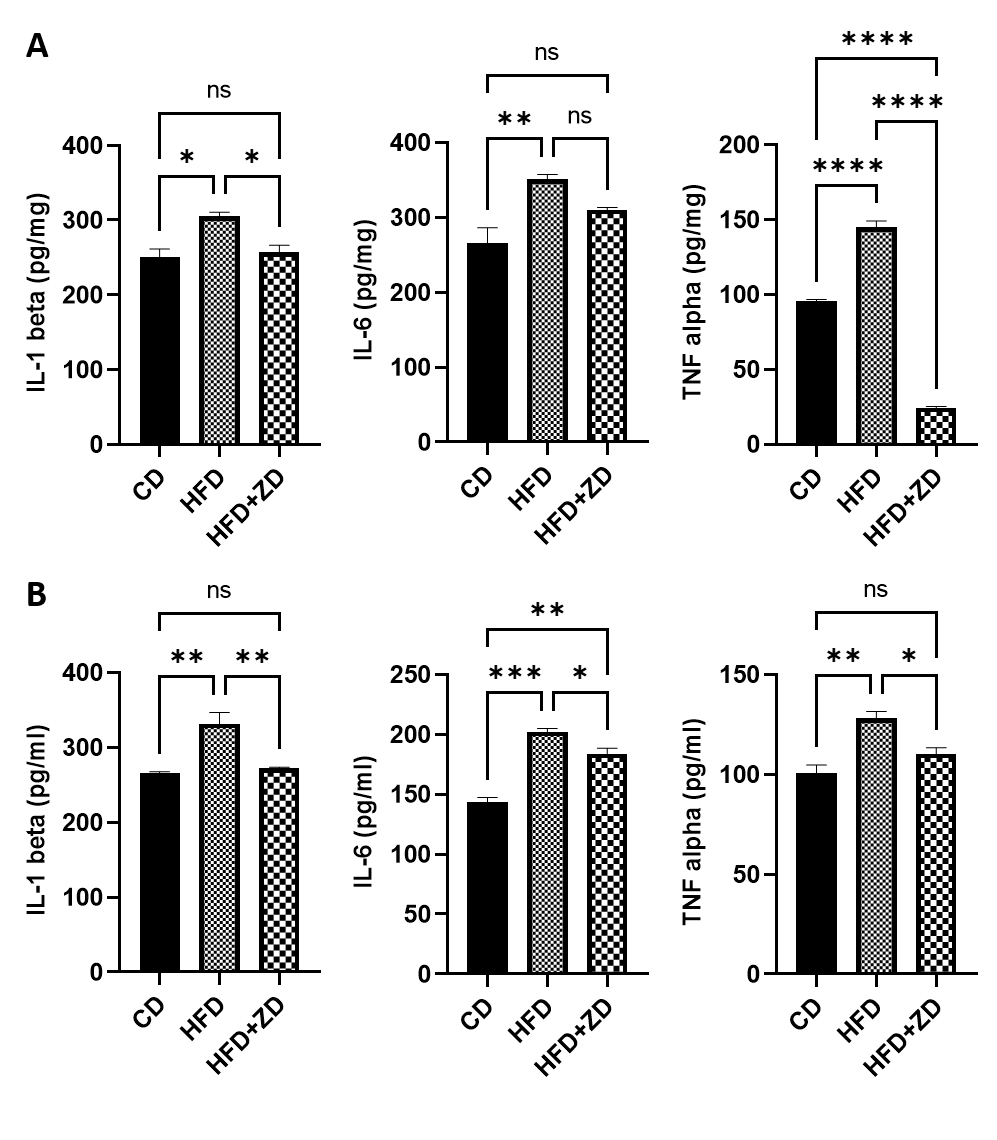
**

**Figure S2** Proinflammatory cytokines in mouse liver (A) and serum (B) samples, detected by ELISA.


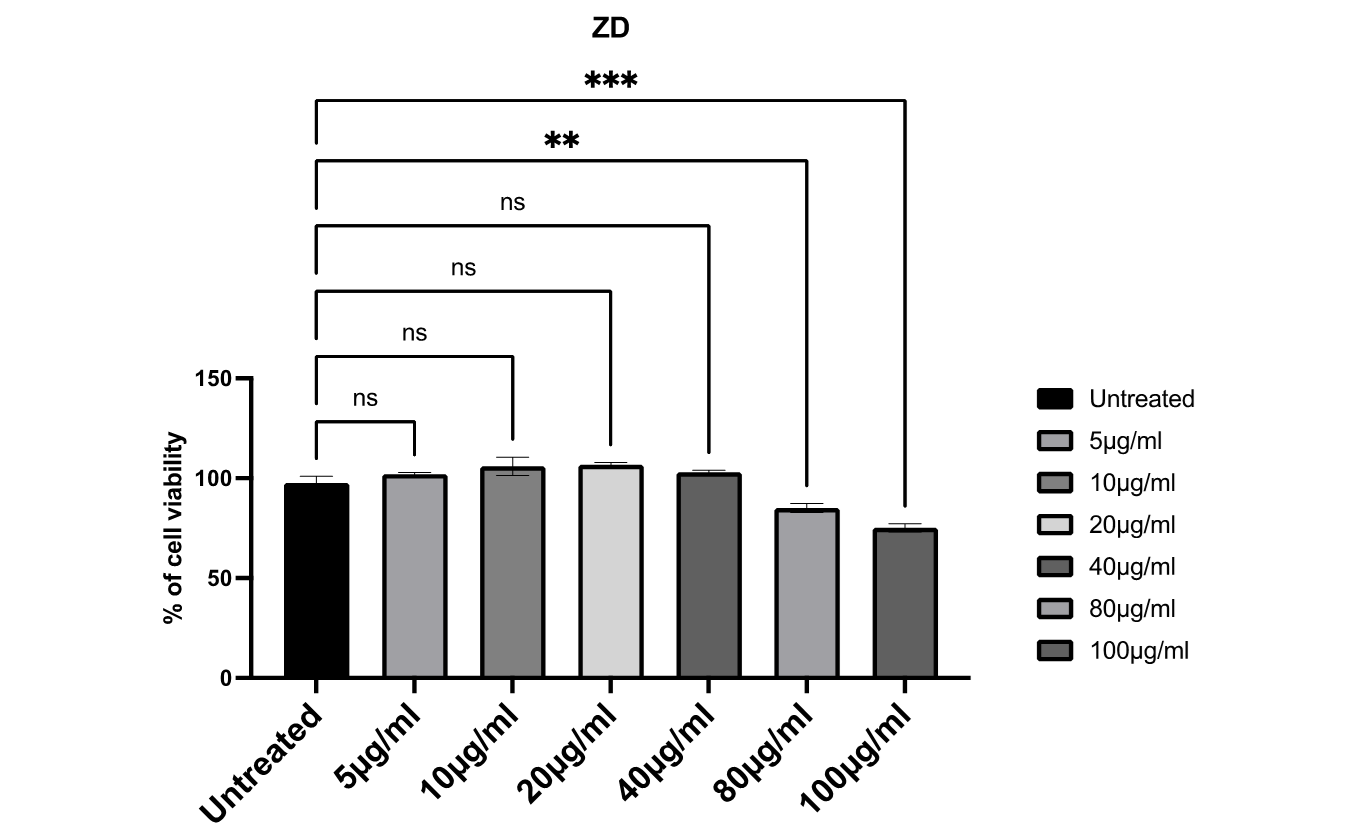


**Figure S3** The effect of Ziyin-Mingmu decoction (ZD) extract on cell viability. ARPE-19 cells were treated with ZD at different concentrations, cell viability was measured by MTT assay.

**Table S1** Primers for qRT-PCR

| Gene (human) | Forward Primer (5’-3’) | Reverse Primer (5’-3’) |
| --- | --- | --- |
| *Catalase* | ATCTCGTTGGAAATAACACC | AGAAACCTGATGCAGAGACT |
| *SOD1* | AGGGCATCATCAATTTCGAG | CATTGCCCAAGTCTCCAAC |
| *SOD2* | GCCTCCCTGACCTGCCTTAC | GTGATTGATATGGCCCCCG |
| *GPX1* | AGTCCACCGTGTATGCCTTC | CTCCTGGTGTCCGAACTGAT |
| *IL-1β* | GCTGAGGAAGATGCTGGTTC | TCCATATCCTGTCCCTGGAG |
| *IL-6* | ATGTAGCCGCCCCACACAGA | CATCCATCTTTTTCAGCCAT |
| *IL-8* | GTGCAGTTTTGCCAAGGAGT | ACTTCTCCACAACCCTCTGC |
| *TNFα* | CCCTGAAAACAACCCTCAGA | CCACGATCAGGAAGGAGAAG |
| *GAPDH* | GAGTCAACGGATTTGGTCGT | TTGATTTTGGAGGGATCTCG |
| Gene (mouse) | Forward Primer (5’-3’) | Reverse Primer (5’-3’) |
| *Abca1* | AGTTTCGGTATGGCGGGTTT | AGCATGCCAGCCCTTGTTAT |
| *Abcg1* | ACCTACCACAACCCAGCAGACTTT | GGTGCCAAAGAAACGGGTTCACAT |
| *Cyp27a1* | GCCTTGCACAAGGAAGTGACT | CGCAGGGTCTCCTTAATCACA |
| *Cyp46a1* | CCCTAGCCTTTCCCCAAATTGC | CGGAAGAATCCCTTGCAACC |
| *Nr1h3*  *(coding* LXRα*)* | CCCTAGCCTTTCCCCAAATTGC | CGGAAGAATCCCTTGCAACC |
| *Catalase* | GCGGACATTCTACACAAAGGT | CAGTGAAATTCTTGACCGCTT |
| *Gpx1* | CGGAATGCCTTGCCAACACC | CAAAGTTCCAGGCAATGTCGTT |
| *Sod1* | GAACCATCCACTTCGAGCAG | CAACATGCCTCTCTTCATCCG |
| *Il-1 β* | CAGGCAGGCAGTATCACTCA | AGCTCATATGGGTCCGACAG |
| *Tnf-α* | ACGTGGAACTGGCAGAAGAG | AGGGTCTGGGCCATAGAACT |
| *Gapdh* | CCCACTAACATCAAATGGGG | CCTTCCACAATGCCAAAGTT |

**Table S2** Basic Statistics of Sequence Reads in Stool Samples in three groups

| Sample-id | Input-reads | Filtered | % of input passed filter | Denoised | Merged | % of input merged | Non-chimeric | % of input non-chimeric |
| --- | --- | --- | --- | --- | --- | --- | --- | --- |
| CL1 | 80107 | 79975 | 99.84 | 77949 | 69151 | 86.3 | 52012 | 64.9 |
| CL2 | 80401 | 80243 | 99.8 | 78581 | 71774 | 89.3 | 59714 | 74.3 |
| CL3 | 80372 | 80223 | 99.81 | 78725 | 71824 | 89.4 | 62216 | 77.4 |
| CL4 | 80160 | 80008 | 99.81 | 78030 | 68839 | 85.9 | 52326 | 65.3 |
| CL5 | 79868 | 79735 | 99.83 | 77591 | 69530 | 87.1 | 54403 | 68.1 |
| CL6 | 79796 | 79639 | 99.8 | 77492 | 67412 | 84.5 | 54307 | 68.1 |
| HFD1 | 79700 | 79526 | 99.78 | 77433 | 70530 | 88.5 | 62450 | 78.4 |
| HFD2 | 114311 | 114049 | 99.77 | 111125 | 101533 | 88.8 | 87845 | 76.9 |
| HFD3 | 80277 | 80171 | 99.87 | 78801 | 76200 | 94.9 | 74165 | 92.4 |
| HFD4 | 80084 | 79945 | 99.83 | 78605 | 75676 | 94.5 | 70913 | 88.6 |
| HFD5 | 79676 | 79519 | 99.8 | 77343 | 70630 | 88.7 | 58551 | 73.5 |
| HFD6 | 79990 | 79823 | 99.79 | 78418 | 75051 | 93.8 | 68764 | 86.0 |
| HFD+ZD1 | 113791 | 113499 | 99.74 | 112027 | 106431 | 93.5 | 91581 | 80.5 |
| HFD+ZD2 | 79962 | 79792 | 99.79 | 78719 | 75018 | 93.8 | 67249 | 84.1 |
| HFD+ZD3 | 79815 | 79639 | 99.78 | 78757 | 75613 | 94.7 | 69267 | 86.8 |
| HFD+ZD4 | 79865 | 79677 | 99.76 | 78704 | 75681 | 94.8 | 66750 | 83.6 |
| HFD+ZD5 | 80063 | 79882 | 99.77 | 78829 | 75487 | 94.3 | 64779 | 80.9 |
| HFD+ZD6 | 80157 | 80004 | 99.81 | 79155 | 76850 | 95.9 | 73671 | 91.9 |
| Total | 1508395 | 1505349 | 1796.38 | 1476284 | 1373230 | 1638.58 | 1190963 | 1421.4 |

**Table S3** Taxonomic profile of individual samples in the three experimental groups: control (CL), high-fat diet (HFD) and high-fat diet fed animals treated with ZD (HFD+ZD)

| Group | CL | CL | CL | CL | CL | CL | HFD | HFD | HFD | HFD | HFD | HFD | HFD+ZD | HFD+ZD | HFD+ZD | HFD+ZD | HFD+ZD | HFD+ZD |
| --- | --- | --- | --- | --- | --- | --- | --- | --- | --- | --- | --- | --- | --- | --- | --- | --- | --- | --- |
| p__Acidobacteriota;c__Acidobacteriae;o__Acidobacteriales | 0.000 | 0.000 | 0.000 | 0.000 | 0.000 | 0.000 | 0.000 | 0.000 | 0.000 | 0.000 | 0.000 | 0.000 | 0.000 | 0.000 | 0.000 | 0.003 | 0.005 | 0.000 |
| p__Acidobacteriota;c__Acidobacteriae;o__Acidobacteriales;f__Koribacteraceae;g__Candidatus_Koribacter | 0.000 | 0.000 | 0.000 | 0.000 | 0.000 | 0.000 | 0.000 | 0.000 | 0.000 | 0.000 | 0.000 | 0.000 | 0.000 | 0.000 | 0.009 | 0.000 | 0.000 | 0.015 |
| p__Acidobacteriota;c__Blastocatellia | 0.000 | 0.000 | 0.000 | 0.000 | 0.000 | 0.000 | 0.009 | 0.000 | 0.006 | 0.000 | 0.000 | 0.000 | 0.000 | 0.000 | 0.000 | 0.000 | 0.000 | 0.000 |
| p__Acidobacteriota;c__Holophagae;o__Subgroup_7;f__Subgroup_7;g__Subgroup_7 | 0.008 | 0.007 | 0.000 | 0.004 | 0.011 | 0.000 | 0.000 | 0.000 | 0.000 | 0.000 | 0.000 | 0.000 | 0.000 | 0.000 | 0.000 | 0.000 | 0.000 | 0.000 |
| p__Acidobacteriota;c__Vicinamibacteria;o__Vicinamibacterales;f__Vicinamibacteraceae | 0.022 | 0.017 | 0.013 | 0.017 | 0.022 | 0.011 | 0.000 | 0.000 | 0.000 | 0.000 | 0.017 | 0.000 | 0.000 | 0.000 | 0.000 | 0.000 | 0.000 | 0.000 |
| p__Acidobacteriota;c__Vicinamibacteria;o__Vicinamibacterales;f__Vicinamibacteraceae;g__Luteitalea | 0.010 | 0.016 | 0.013 | 0.012 | 0.017 | 0.011 | 0.004 | 0.000 | 0.000 | 0.018 | 0.015 | 0.000 | 0.000 | 0.000 | 0.000 | 0.000 | 0.000 | 0.000 |
| p__Acidobacteriota;c__Vicinamibacteria;o__Vicinamibacterales;f__Vicinamibacteraceae;g__Vicinamibacteraceae | 0.000 | 0.000 | 0.000 | 0.000 | 0.000 | 0.000 | 0.000 | 0.023 | 0.000 | 0.000 | 0.000 | 0.000 | 0.010 | 0.000 | 0.000 | 0.000 | 0.000 | 0.000 |
| p__Actinobacteriota;c__Acidimicrobiia | 0.000 | 0.000 | 0.000 | 0.000 | 0.000 | 0.000 | 0.000 | 0.110 | 0.000 | 0.151 | 0.000 | 0.000 | 0.000 | 0.000 | 0.003 | 0.000 | 0.003 | 0.000 |
| p__Actinobacteriota;c__Acidimicrobiia;o__Actinomarinales;f__Actinomarinaceae;g__Candidatus_Actinomarina | 0.000 | 0.000 | 0.000 | 0.000 | 0.000 | 0.000 | 0.000 | 0.021 | 0.000 | 0.007 | 0.000 | 0.000 | 0.000 | 0.000 | 0.000 | 0.000 | 0.000 | 0.000 |
| p__Actinobacteriota;c__Acidimicrobiia;o__Actinomarinales;f__uncultured;g__uncultured | 0.012 | 0.009 | 0.000 | 0.000 | 0.000 | 0.000 | 0.000 | 0.000 | 0.000 | 0.000 | 0.000 | 0.008 | 0.000 | 0.003 | 0.000 | 0.000 | 0.000 | 0.000 |
| p__Actinobacteriota;c__Acidimicrobiia;o__IMCC26256;f__IMCC26256;g__IMCC26256 | 0.037 | 0.000 | 0.000 | 0.012 | 0.019 | 0.000 | 0.000 | 0.000 | 0.000 | 0.000 | 0.059 | 0.000 | 0.007 | 0.000 | 0.000 | 0.000 | 0.000 | 0.000 |
| p__Actinobacteriota;c__Acidimicrobiia;o__Microtrichales;f__Iamiaceae;g__Iamia | 0.010 | 0.000 | 0.000 | 0.000 | 0.011 | 0.009 | 0.000 | 0.000 | 0.000 | 0.000 | 0.000 | 0.000 | 0.009 | 0.000 | 0.000 | 0.000 | 0.000 | 0.000 |
| p__Actinobacteriota;c__Acidimicrobiia;o__Microtrichales;f__Ilumatobacteraceae | 0.000 | 0.000 | 0.000 | 0.000 | 0.000 | 0.000 | 0.000 | 0.000 | 0.000 | 0.000 | 0.043 | 0.000 | 0.012 | 0.000 | 0.000 | 0.000 | 0.000 | 0.000 |
| p__Actinobacteriota;c__Acidimicrobiia;o__Microtrichales;f__uncultured;g__uncultured | 0.000 | 0.000 | 0.000 | 0.000 | 0.000 | 0.000 | 0.003 | 0.000 | 0.000 | 0.005 | 0.000 | 0.000 | 0.000 | 0.000 | 0.000 | 0.000 | 0.000 | 0.000 |
| p__Actinobacteriota;c__Actinobacteria;o__Bifidobacteriales;f__Bifidobacteriaceae;g__Bifidobacterium | 0.059 | 0.019 | 0.039 | 0.093 | 0.009 | 0.034 | 0.226 | 0.051 | 0.031 | 0.023 | 0.042 | 0.153 | 0.035 | 0.036 | 0.020 | 0.048 | 0.051 | 0.127 |
| p__Actinobacteriota;c__Actinobacteria;o__Corynebacteriales;f__Corynebacteriaceae;g__Corynebacterium | 0.000 | 0.000 | 0.000 | 0.000 | 0.000 | 0.000 | 0.011 | 0.027 | 0.000 | 0.017 | 0.006 | 0.012 | 0.000 | 0.000 | 0.000 | 0.000 | 0.000 | 0.000 |
| p__Actinobacteriota;c__Actinobacteria;o__Corynebacteriales;f__Dietziaceae;g__Dietzia | 0.000 | 0.000 | 0.000 | 0.000 | 0.000 | 0.000 | 0.000 | 0.019 | 0.000 | 0.000 | 0.000 | 0.011 | 0.014 | 0.000 | 0.000 | 0.003 | 0.006 | 0.005 |
| p__Actinobacteriota;c__Actinobacteria;o__Frankiales;f__uncultured;g__uncultured | 0.000 | 0.000 | 0.000 | 0.000 | 0.000 | 0.000 | 0.000 | 0.012 | 0.000 | 0.000 | 0.017 | 0.000 | 0.000 | 0.000 | 0.000 | 0.000 | 0.000 | 0.000 |
| p__Actinobacteriota;c__Actinobacteria;o__Micrococcales;f__Brevibacteriaceae;g__Brevibacterium | 0.000 | 0.000 | 0.000 | 0.000 | 0.000 | 0.000 | 0.000 | 0.000 | 0.000 | 0.000 | 0.000 | 0.004 | 0.000 | 0.000 | 0.000 | 0.000 | 0.000 | 0.007 |
| p__Actinobacteriota;c__Actinobacteria;o__Micrococcales;f__Microbacteriaceae | 0.000 | 0.000 | 0.000 | 0.000 | 0.000 | 0.000 | 0.018 | 0.014 | 0.013 | 0.000 | 0.000 | 0.019 | 0.000 | 0.000 | 0.000 | 0.000 | 0.000 | 0.000 |
| p__Actinobacteriota;c__Actinobacteria;o__Micrococcales;f__Micrococcaceae | 0.000 | 0.010 | 0.010 | 0.019 | 0.015 | 0.000 | 0.013 | 0.019 | 0.000 | 0.000 | 0.049 | 0.000 | 0.014 | 0.000 | 0.000 | 0.000 | 0.000 | 0.000 |
| p__Actinobacteriota;c__Actinobacteria;o__Propionibacteriales;f__Nocardioidaceae | 0.000 | 0.000 | 0.000 | 0.000 | 0.000 | 0.000 | 0.000 | 0.000 | 0.000 | 0.000 | 0.109 | 0.000 | 0.090 | 0.000 | 0.000 | 0.000 | 0.000 | 0.000 |
| p__Actinobacteriota;c__Actinobacteria;o__Propionibacteriales;f__Nocardioidaceae;g__Aeromicrobium | 0.000 | 0.000 | 0.000 | 0.000 | 0.000 | 0.000 | 0.000 | 0.000 | 0.000 | 0.000 | 0.015 | 0.000 | 0.022 | 0.000 | 0.000 | 0.000 | 0.000 | 0.000 |
| p__Actinobacteriota;c__Actinobacteria;o__Pseudonocardiales;f__Pseudonocardiaceae;g__Lechevalieria | 0.000 | 0.000 | 0.000 | 0.000 | 0.000 | 0.000 | 0.000 | 0.000 | 0.000 | 0.000 | 0.036 | 0.000 | 0.022 | 0.000 | 0.000 | 0.000 | 0.000 | 0.000 |
| p__Actinobacteriota;c__Actinobacteria;o__Streptomycetales;f__Streptomycetaceae;g__Streptomyces | 0.029 | 0.024 | 0.000 | 0.000 | 0.000 | 0.000 | 0.000 | 0.000 | 0.000 | 0.000 | 0.044 | 0.000 | 0.027 | 0.000 | 0.000 | 0.000 | 0.000 | 0.000 |
| p__Actinobacteriota;c__Coriobacteriia;o__Coriobacteriales;f__Atopobiaceae | 0.177 | 0.038 | 0.182 | 0.201 | 0.052 | 0.090 | 21.850 | 7.225 | 0.097 | 0.361 | 3.250 | 27.740 | 0.147 | 3.194 | 2.241 | 0.113 | 0.387 | 2.652 |
| p__Actinobacteriota;c__Coriobacteriia;o__Coriobacteriales;f__Atopobiaceae;g__Coriobacteriaceae_UCG-002 | 0.000 | 0.000 | 0.000 | 0.006 | 0.000 | 0.000 | 0.000 | 0.000 | 0.000 | 0.003 | 0.000 | 0.014 | 0.000 | 0.000 | 0.000 | 0.000 | 0.000 | 0.000 |
| p__Actinobacteriota;c__Coriobacteriia;o__Coriobacteriales;f__Coriobacteriales_Incertae_Sedis;g__uncultured | 0.000 | 0.007 | 0.010 | 0.000 | 0.007 | 0.000 | 0.000 | 0.000 | 0.000 | 0.000 | 0.000 | 0.000 | 0.000 | 0.000 | 0.000 | 0.000 | 0.000 | 0.000 |
| p__Actinobacteriota;c__Coriobacteriia;o__Coriobacteriales;f__Eggerthellaceae | 0.954 | 1.679 | 2.111 | 0.758 | 0.859 | 0.497 | 0.013 | 0.975 | 0.010 | 0.524 | 0.206 | 0.075 | 0.183 | 1.000 | 0.075 | 0.065 | 0.036 | 0.336 |
| p__Actinobacteriota;c__Coriobacteriia;o__Coriobacteriales;f__Eggerthellaceae;g__DNF00809 | 0.092 | 0.055 | 0.326 | 0.029 | 0.017 | 0.047 | 0.000 | 0.000 | 0.000 | 0.000 | 0.000 | 0.000 | 0.000 | 0.000 | 0.000 | 0.000 | 0.000 | 0.000 |
| p__Actinobacteriota;c__Coriobacteriia;o__Coriobacteriales;f__Eggerthellaceae;g__Enterorhabdus | 0.153 | 0.292 | 0.357 | 0.179 | 0.313 | 0.126 | 3.896 | 5.928 | 1.387 | 2.079 | 1.158 | 2.676 | 0.816 | 2.821 | 1.647 | 1.645 | 1.251 | 2.131 |
| p__Actinobacteriota;c__Coriobacteriia;o__Coriobacteriales;f__Eggerthellaceae;g__Parvibacter | 0.000 | 0.010 | 0.222 | 0.000 | 0.000 | 0.000 | 0.000 | 0.000 | 0.000 | 0.000 | 0.000 | 0.026 | 0.000 | 0.027 | 0.000 | 0.000 | 0.000 | 0.000 |
| p__Actinobacteriota;c__Coriobacteriia;o__Coriobacteriales;f__Eggerthellaceae;g__uncultured | 0.000 | 0.012 | 0.000 | 0.000 | 0.019 | 0.011 | 0.000 | 0.000 | 0.000 | 0.000 | 0.000 | 0.000 | 0.000 | 0.000 | 0.000 | 0.000 | 0.000 | 0.000 |
| p__Actinobacteriota;c__MB-A2-108;o__MB-A2-108;f__MB-A2-108;g__MB-A2-108 | 0.000 | 0.000 | 0.000 | 0.000 | 0.000 | 0.000 | 0.000 | 0.000 | 0.000 | 0.000 | 0.225 | 0.000 | 0.075 | 0.000 | 0.000 | 0.000 | 0.000 | 0.000 |
| p__Armatimonadota;c__uncultured;o__uncultured;f__uncultured;g__uncultured | 0.000 | 0.000 | 0.000 | 0.012 | 0.000 | 0.000 | 0.000 | 0.000 | 0.000 | 0.000 | 0.005 | 0.000 | 0.000 | 0.000 | 0.000 | 0.000 | 0.000 | 0.000 |
| p__Bacteroidota;c__Bacteroidia | 0.381 | 0.268 | 0.334 | 0.318 | 0.250 | 0.378 | 0.030 | 0.028 | 0.283 | 0.030 | 0.164 | 0.081 | 0.045 | 0.161 | 0.010 | 0.006 | 0.142 | 0.108 |
| p__Bacteroidota;c__Bacteroidia;o__Bacteroidales | 0.000 | 0.010 | 0.005 | 0.000 | 0.000 | 0.000 | 0.000 | 0.000 | 0.000 | 0.000 | 0.000 | 0.000 | 0.000 | 0.000 | 0.000 | 0.000 | 0.000 | 0.000 |
| p__Bacteroidota;c__Bacteroidia;o__Bacteroidales;f__Bacteroidaceae;g__Bacteroides | 0.995 | 1.002 | 0.359 | 1.578 | 1.684 | 1.993 | 0.089 | 0.113 | 0.682 | 2.064 | 0.652 | 0.281 | 0.633 | 0.393 | 1.275 | 0.203 | 2.494 | 1.842 |
| p__Bacteroidota;c__Bacteroidia;o__Bacteroidales;f__Barnesiellaceae;g__Coprobacter | 0.000 | 0.009 | 0.006 | 0.000 | 0.000 | 0.000 | 0.006 | 0.012 | 0.000 | 0.012 | 0.004 | 0.000 | 0.000 | 0.000 | 0.000 | 0.000 | 0.000 | 0.000 |
| p__Bacteroidota;c__Bacteroidia;o__Bacteroidales;f__Marinifilaceae;g__Butyricimonas | 0.010 | 0.016 | 0.024 | 0.019 | 0.000 | 0.026 | 0.000 | 0.000 | 0.009 | 0.037 | 0.020 | 0.000 | 0.039 | 0.012 | 0.019 | 0.000 | 0.015 | 0.019 |
| p__Bacteroidota;c__Bacteroidia;o__Bacteroidales;f__Marinifilaceae;g__Odoribacter | 0.000 | 0.000 | 0.005 | 0.000 | 0.000 | 0.000 | 0.000 | 0.000 | 0.000 | 0.007 | 0.010 | 0.000 | 0.000 | 0.000 | 0.000 | 0.000 | 0.000 | 0.000 |
| p__Bacteroidota;c__Bacteroidia;o__Bacteroidales;f__Muribaculaceae | 1.661 | 3.968 | 3.168 | 2.008 | 2.935 | 3.916 | 0.000 | 0.000 | 0.000 | 0.022 | 0.020 | 0.000 | 0.000 | 0.000 | 0.000 | 0.000 | 0.000 | 0.000 |
| p__Bacteroidota;c__Bacteroidia;o__Bacteroidales;f__Muribaculaceae;g__Muribaculaceae | 15.734 | 15.736 | 25.731 | 11.046 | 13.453 | 12.084 | 0.033 | 0.062 | 0.032 | 0.428 | 0.308 | 0.148 | 0.151 | 0.227 | 0.200 | 0.081 | 0.107 | 0.720 |
| p__Bacteroidota;c__Bacteroidia;o__Bacteroidales;f__Muribaculaceae;g__Muribaculum | 5.205 | 6.454 | 5.004 | 8.724 | 7.971 | 4.685 | 0.000 | 0.018 | 0.000 | 0.045 | 0.075 | 0.038 | 0.000 | 0.000 | 0.000 | 0.000 | 0.005 | 0.000 |
| p__Bacteroidota;c__Bacteroidia;o__Bacteroidales;f__Prevotellaceae | 1.199 | 0.119 | 0.445 | 0.204 | 0.514 | 0.647 | 0.000 | 0.007 | 0.004 | 0.005 | 0.008 | 0.000 | 0.000 | 0.004 | 0.000 | 0.000 | 0.000 | 0.000 |
| p__Bacteroidota;c__Bacteroidia;o__Bacteroidales;f__Prevotellaceae;g__Alloprevotella | 9.035 | 4.758 | 2.586 | 4.405 | 2.661 | 3.400 | 0.048 | 0.032 | 0.158 | 0.863 | 0.408 | 0.148 | 0.015 | 0.084 | 0.778 | 0.012 | 0.025 | 0.315 |
| p__Bacteroidota;c__Bacteroidia;o__Bacteroidales;f__Prevotellaceae;g__Paraprevotella | 0.006 | 0.009 | 0.010 | 0.000 | 0.009 | 0.000 | 0.000 | 0.000 | 0.000 | 0.000 | 0.000 | 0.000 | 0.002 | 0.012 | 0.000 | 0.000 | 0.008 | 0.000 |
| p__Bacteroidota;c__Bacteroidia;o__Bacteroidales;f__Prevotellaceae;g__Prevotella | 0.002 | 0.009 | 0.000 | 0.017 | 0.011 | 0.058 | 0.004 | 0.000 | 0.000 | 0.000 | 0.006 | 0.000 | 0.000 | 0.000 | 0.000 | 0.000 | 0.000 | 0.000 |
| p__Bacteroidota;c__Bacteroidia;o__Bacteroidales;f__Prevotellaceae;g__Prevotellaceae_UCG-001 | 0.000 | 0.000 | 0.000 | 0.000 | 0.000 | 0.000 | 0.000 | 0.000 | 0.000 | 0.000 | 0.006 | 0.000 | 0.006 | 0.000 | 0.000 | 0.000 | 0.000 | 0.000 |
| p__Bacteroidota;c__Bacteroidia;o__Bacteroidales;f__Rikenellaceae;g__Alistipes | 4.348 | 1.430 | 3.457 | 6.698 | 8.935 | 2.397 | 0.000 | 0.019 | 0.016 | 0.156 | 0.111 | 0.139 | 0.054 | 0.069 | 0.014 | 0.020 | 0.011 | 0.000 |
| p__Bacteroidota;c__Bacteroidia;o__Bacteroidales;f__Rikenellaceae;g__Rikenellaceae | 0.000 | 0.000 | 0.000 | 0.006 | 0.000 | 0.000 | 0.000 | 0.000 | 0.000 | 0.000 | 0.000 | 0.000 | 0.003 | 0.000 | 0.000 | 0.000 | 0.000 | 0.000 |
| p__Bacteroidota;c__Bacteroidia;o__Bacteroidales;f__Rikenellaceae;g__Rikenellaceae_RC9_gut_group | 0.020 | 0.038 | 0.018 | 0.060 | 0.009 | 0.058 | 0.010 | 0.000 | 0.016 | 0.000 | 0.015 | 0.005 | 0.004 | 0.018 | 0.012 | 0.000 | 0.048 | 0.029 |
| p__Bacteroidota;c__Bacteroidia;o__Bacteroidales;f__Tannerellaceae | 0.027 | 0.010 | 0.000 | 0.025 | 0.048 | 0.024 | 0.000 | 0.000 | 0.015 | 0.037 | 0.030 | 0.000 | 0.014 | 0.028 | 0.046 | 0.000 | 0.204 | 0.000 |
| p__Bacteroidota;c__Bacteroidia;o__Bacteroidales;f__Tannerellaceae;g__Parabacteroides | 0.249 | 0.347 | 0.099 | 0.332 | 0.173 | 0.657 | 0.033 | 0.039 | 0.297 | 1.661 | 1.030 | 0.060 | 0.129 | 0.328 | 0.481 | 0.075 | 1.015 | 0.235 |
| p__Bacteroidota;c__Bacteroidia;o__Chitinophagales;f__Chitinophagaceae | 0.010 | 0.000 | 0.010 | 0.000 | 0.000 | 0.000 | 0.000 | 0.000 | 0.000 | 0.000 | 0.011 | 0.000 | 0.000 | 0.000 | 0.000 | 0.000 | 0.000 | 0.000 |
| p__Bacteroidota;c__Bacteroidia;o__Chitinophagales;f__Chitinophagaceae;g__Flavihumibacter | 0.010 | 0.000 | 0.000 | 0.006 | 0.000 | 0.011 | 0.000 | 0.000 | 0.000 | 0.010 | 0.000 | 0.000 | 0.000 | 0.000 | 0.000 | 0.000 | 0.000 | 0.000 |
| p__Bacteroidota;c__Bacteroidia;o__Chitinophagales;f__Chitinophagaceae;g__Flavisolibacter | 0.000 | 0.000 | 0.011 | 0.012 | 0.013 | 0.000 | 0.000 | 0.000 | 0.000 | 0.000 | 0.012 | 0.000 | 0.000 | 0.000 | 0.000 | 0.000 | 0.000 | 0.000 |
| p__Bacteroidota;c__Bacteroidia;o__Chitinophagales;f__Chitinophagaceae;g__Niabella | 0.008 | 0.000 | 0.000 | 0.000 | 0.000 | 0.008 | 0.000 | 0.000 | 0.000 | 0.007 | 0.000 | 0.000 | 0.000 | 0.000 | 0.000 | 0.000 | 0.000 | 0.000 |
| p__Bacteroidota;c__Bacteroidia;o__Chitinophagales;f__Chitinophagaceae;g__Parasegetibacter | 0.016 | 0.009 | 0.000 | 0.000 | 0.000 | 0.000 | 0.003 | 0.000 | 0.003 | 0.000 | 0.013 | 0.000 | 0.000 | 0.000 | 0.000 | 0.000 | 0.000 | 0.000 |
| p__Bacteroidota;c__Bacteroidia;o__Cytophagales;f__Cyclobacteriaceae;g__Reichenbachiella | 0.000 | 0.000 | 0.000 | 0.000 | 0.000 | 0.000 | 0.000 | 0.009 | 0.000 | 0.025 | 0.000 | 0.000 | 0.000 | 0.000 | 0.000 | 0.000 | 0.000 | 0.000 |
| p__Bacteroidota;c__Bacteroidia;o__Cytophagales;f__Hymenobacteraceae;g__Adhaeribacter | 0.020 | 0.000 | 0.000 | 0.000 | 0.000 | 0.000 | 0.000 | 0.000 | 0.000 | 0.007 | 0.007 | 0.000 | 0.000 | 0.000 | 0.000 | 0.000 | 0.000 | 0.000 |
| p__Bacteroidota;c__Bacteroidia;o__Cytophagales;f__Microscillaceae;g__uncultured | 0.004 | 0.005 | 0.000 | 0.015 | 0.019 | 0.000 | 0.000 | 0.000 | 0.000 | 0.012 | 0.017 | 0.000 | 0.000 | 0.000 | 0.000 | 0.000 | 0.000 | 0.000 |
| p__Bacteroidota;c__Bacteroidia;o__Flavobacteriales | 0.008 | 0.000 | 0.000 | 0.000 | 0.000 | 0.000 | 0.000 | 0.000 | 0.000 | 0.000 | 0.007 | 0.000 | 0.000 | 0.000 | 0.000 | 0.000 | 0.000 | 0.000 |
| p__Bacteroidota;c__Bacteroidia;o__Flavobacteriales;f__Flavobacteriaceae | 0.000 | 0.000 | 0.000 | 0.000 | 0.000 | 0.000 | 0.000 | 0.007 | 0.000 | 0.054 | 0.000 | 0.000 | 0.000 | 0.000 | 0.000 | 0.000 | 0.000 | 0.003 |
| p__Bacteroidota;c__Bacteroidia;o__Flavobacteriales;f__Flavobacteriaceae;g__Flavobacterium | 0.031 | 0.031 | 0.015 | 0.019 | 0.013 | 0.013 | 0.000 | 0.000 | 0.000 | 0.012 | 0.015 | 0.000 | 0.000 | 0.000 | 0.000 | 0.000 | 0.000 | 0.000 |
| p__Bacteroidota;c__Bacteroidia;o__Flavobacteriales;f__Flavobacteriaceae;g__Formosa | 0.065 | 0.041 | 0.036 | 0.068 | 0.024 | 0.028 | 0.027 | 0.060 | 0.038 | 0.017 | 0.045 | 0.027 | 0.023 | 0.016 | 0.006 | 0.021 | 0.020 | 0.008 |
| p__Bacteroidota;c__Bacteroidia;o__Flavobacteriales;f__Flavobacteriaceae;g__Hoppeia | 0.000 | 0.000 | 0.000 | 0.006 | 0.007 | 0.000 | 0.000 | 0.000 | 0.000 | 0.000 | 0.000 | 0.000 | 0.000 | 0.000 | 0.000 | 0.000 | 0.000 | 0.000 |
| p__Bacteroidota;c__Bacteroidia;o__Flavobacteriales;f__Flavobacteriaceae;g__Lutimonas | 0.004 | 0.000 | 0.010 | 0.000 | 0.007 | 0.000 | 0.000 | 0.002 | 0.003 | 0.000 | 0.000 | 0.000 | 0.000 | 0.000 | 0.000 | 0.000 | 0.000 | 0.000 |
| p__Bacteroidota;c__Bacteroidia;o__Flavobacteriales;f__Flavobacteriaceae;g__Meridianimaribacter | 0.000 | 0.005 | 0.015 | 0.000 | 0.000 | 0.000 | 0.000 | 0.000 | 0.000 | 0.000 | 0.000 | 0.000 | 0.000 | 0.000 | 0.000 | 0.000 | 0.000 | 0.000 |
| p__Bacteroidota;c__Bacteroidia;o__Flavobacteriales;f__Flavobacteriaceae;g__Sungkyunkwania | 0.004 | 0.010 | 0.000 | 0.000 | 0.000 | 0.006 | 0.000 | 0.000 | 0.000 | 0.000 | 0.008 | 0.000 | 0.000 | 0.000 | 0.000 | 0.000 | 0.000 | 0.000 |
| p__Bacteroidota;c__Bacteroidia;o__Flavobacteriales;f__Flavobacteriaceae;g__uncultured | 0.000 | 0.012 | 0.000 | 0.000 | 0.000 | 0.000 | 0.000 | 0.000 | 0.000 | 0.010 | 0.000 | 0.000 | 0.000 | 0.004 | 0.007 | 0.000 | 0.000 | 0.031 |
| p__Bacteroidota;c__Bacteroidia;o__Flavobacteriales;f__Flavobacteriales;g__Flavobacteriales | 0.000 | 0.000 | 0.015 | 0.008 | 0.000 | 0.008 | 0.009 | 0.018 | 0.004 | 0.008 | 0.007 | 0.000 | 0.000 | 0.000 | 0.000 | 0.005 | 0.000 | 0.000 |
| p__Bacteroidota;c__Bacteroidia;o__Flavobacteriales;f__Weeksellaceae;g__Empedobacter | 0.000 | 0.009 | 0.005 | 0.000 | 0.000 | 0.000 | 0.000 | 0.000 | 0.000 | 0.007 | 0.005 | 0.000 | 0.000 | 0.000 | 0.000 | 0.000 | 0.000 | 0.000 |
| p__Bacteroidota;c__Bacteroidia;o__Sphingobacteriales;f__Sphingobacteriaceae;g__Anseongella | 0.000 | 0.005 | 0.000 | 0.000 | 0.000 | 0.000 | 0.000 | 0.000 | 0.000 | 0.000 | 0.004 | 0.000 | 0.000 | 0.000 | 0.000 | 0.000 | 0.000 | 0.000 |
| p__Bacteroidota;c__Bacteroidia;o__Sphingobacteriales;f__Sphingobacteriaceae;g__Sphingobacterium | 0.008 | 0.010 | 0.000 | 0.010 | 0.011 | 0.008 | 0.000 | 0.000 | 0.000 | 0.005 | 0.012 | 0.000 | 0.000 | 0.000 | 0.000 | 0.000 | 0.000 | 0.000 |
| p__Campilobacterota;c__Campylobacteria;o__Campylobacterales;f__Helicobacteraceae;g__Helicobacter | 0.579 | 0.781 | 1.583 | 1.342 | 2.255 | 1.074 | 0.180 | 0.071 | 1.198 | 1.344 | 1.849 | 1.197 | 0.071 | 0.143 | 0.236 | 0.000 | 0.050 | 0.557 |
| p__Chloroflexi;c__Anaerolineae;o__SBR1031;f__A4b;g__OLB13 | 0.000 | 0.003 | 0.000 | 0.000 | 0.000 | 0.000 | 0.000 | 0.000 | 0.000 | 0.000 | 0.004 | 0.000 | 0.000 | 0.000 | 0.000 | 0.000 | 0.000 | 0.000 |
| p__Chloroflexi;c__Anaerolineae;o__SBR1031;f__SBR1031;g__SBR1031 | 0.000 | 0.000 | 0.000 | 0.000 | 0.000 | 0.000 | 0.003 | 0.000 | 0.000 | 0.000 | 0.000 | 0.003 | 0.000 | 0.000 | 0.000 | 0.000 | 0.000 | 0.000 |
| p__Chloroflexi;c__Chloroflexia;o__Chloroflexales;f__Herpetosiphonaceae;g__Herpetosiphon | 0.006 | 0.000 | 0.000 | 0.008 | 0.009 | 0.008 | 0.000 | 0.000 | 0.000 | 0.000 | 0.005 | 0.000 | 0.000 | 0.000 | 0.000 | 0.000 | 0.000 | 0.000 |
| p__Chloroflexi;c__Chloroflexia;o__Thermomicrobiales;f__JG30-KF-CM45;g__JG30-KF-CM45 | 0.006 | 0.000 | 0.000 | 0.006 | 0.000 | 0.000 | 0.000 | 0.000 | 0.000 | 0.000 | 0.000 | 0.000 | 0.000 | 0.000 | 0.000 | 0.000 | 0.000 | 0.000 |
| p__Chloroflexi;c__Gitt-GS-136;o__Gitt-GS-136;f__Gitt-GS-136;g__Gitt-GS-136 | 0.000 | 0.000 | 0.013 | 0.000 | 0.000 | 0.006 | 0.000 | 0.000 | 0.000 | 0.000 | 0.000 | 0.000 | 0.000 | 0.000 | 0.000 | 0.000 | 0.000 | 0.000 |
| p__Chloroflexi;c__KD4-96;o__KD4-96;f__KD4-96;g__KD4-96 | 0.000 | 0.000 | 0.000 | 0.000 | 0.000 | 0.000 | 0.000 | 0.000 | 0.000 | 0.000 | 0.000 | 0.000 | 0.000 | 0.000 | 0.000 | 0.009 | 0.009 | 0.000 |
| p__Cyanobacteria;c__Cyanobacteriia;o__Chloroplast;f__Chloroplast;g__Chloroplast | 0.037 | 0.019 | 0.031 | 0.087 | 0.086 | 0.077 | 0.040 | 0.073 | 0.058 | 1.567 | 0.074 | 0.041 | 0.038 | 0.007 | 0.012 | 0.021 | 0.040 | 0.027 |
| p__Cyanobacteria;c__Cyanobacteriia;o__Cyanobacteriales;f__Xenococcaceae;g__Pleurocapsa_PCC-7319 | 0.000 | 0.000 | 0.000 | 0.000 | 0.000 | 0.000 | 0.000 | 0.009 | 0.000 | 0.112 | 0.000 | 0.000 | 0.000 | 0.000 | 0.000 | 0.000 | 0.005 | 0.011 |
| p__Cyanobacteria;c__Vampirivibrionia;o__Gastranaerophilales;f__Gastranaerophilales;g__Gastranaerophilales | 0.004 | 0.000 | 0.008 | 0.000 | 0.000 | 0.000 | 0.000 | 0.000 | 0.000 | 0.000 | 0.000 | 0.000 | 0.000 | 0.003 | 0.000 | 0.000 | 0.000 | 0.000 |
| p__Deferribacterota;c__Deferribacteres;o__Deferribacterales;f__Deferribacteraceae;g__Mucispirillum | 0.039 | 0.022 | 0.153 | 0.366 | 0.078 | 0.183 | 0.028 | 0.012 | 1.350 | 0.265 | 0.644 | 0.093 | 0.004 | 0.009 | 0.006 | 0.000 | 0.025 | 0.020 |
| p__Desulfobacterota;c__Desulfovibrionia;o__Desulfovibrionales;f__Desulfovibrionaceae | 5.180 | 0.599 | 1.520 | 4.355 | 2.120 | 7.783 | 6.907 | 7.773 | 22.966 | 17.147 | 36.873 | 8.184 | 27.871 | 16.381 | 17.813 | 11.077 | 31.917 | 17.925 |
| p__Desulfobacterota;c__Desulfovibrionia;o__Desulfovibrionales;f__Desulfovibrionaceae;g__Bilophila | 0.000 | 0.000 | 0.000 | 0.006 | 0.000 | 0.000 | 0.000 | 0.000 | 0.000 | 0.000 | 0.006 | 0.000 | 0.000 | 0.000 | 0.000 | 0.000 | 0.000 | 0.000 |
| p__Desulfobacterota;c__Desulfovibrionia;o__Desulfovibrionales;f__Desulfovibrionaceae;g__Desulfovibrio | 1.350 | 9.548 | 8.487 | 3.020 | 6.158 | 1.016 | 0.148 | 1.817 | 1.446 | 4.053 | 1.128 | 0.255 | 10.531 | 7.834 | 10.056 | 20.842 | 14.362 | 12.557 |
| p__Desulfobacterota;c__Desulfovibrionia;o__Desulfovibrionales;f__Desulfovibrionaceae;g__uncultured | 0.110 | 0.021 | 0.032 | 0.123 | 0.006 | 0.207 | 0.260 | 0.424 | 0.508 | 1.408 | 0.513 | 0.387 | 0.596 | 0.064 | 0.759 | 0.437 | 0.740 | 0.554 |
| p__Firmicutes | 0.000 | 0.000 | 0.000 | 0.000 | 0.000 | 0.000 | 0.006 | 0.000 | 0.012 | 0.032 | 0.011 | 0.000 | 0.000 | 0.000 | 0.000 | 0.000 | 0.000 | 0.000 |
| p__Firmicutes;c__Bacilli;o__Bacillales;f__Bacillaceae;g__Bacillus | 0.000 | 0.000 | 0.000 | 0.000 | 0.000 | 0.000 | 0.013 | 0.019 | 0.003 | 0.000 | 0.004 | 0.007 | 0.013 | 0.000 | 0.000 | 0.000 | 0.000 | 0.000 |
| p__Firmicutes;c__Bacilli;o__Bacillales;f__Planococcaceae | 0.000 | 0.000 | 0.000 | 0.000 | 0.000 | 0.000 | 0.000 | 0.000 | 0.013 | 0.015 | 0.008 | 0.005 | 0.000 | 0.000 | 0.000 | 0.000 | 0.000 | 0.000 |
| p__Firmicutes;c__Bacilli;o__Bacillales;f__Planococcaceae;g__Sporosarcina | 0.000 | 0.000 | 0.000 | 0.000 | 0.000 | 0.000 | 0.024 | 0.000 | 0.018 | 0.035 | 0.012 | 0.019 | 0.000 | 0.000 | 0.000 | 0.000 | 0.000 | 0.000 |
| p__Firmicutes;c__Bacilli;o__Entomoplasmatales;f__Entomoplasmataceae;g__Entomoplasma | 0.000 | 0.000 | 0.000 | 0.000 | 0.000 | 0.000 | 0.007 | 0.011 | 0.009 | 0.000 | 0.000 | 0.008 | 0.000 | 0.000 | 0.000 | 0.000 | 0.000 | 0.000 |
| p__Firmicutes;c__Bacilli;o__Erysipelotrichales;f__Erysipelatoclostridiaceae | 0.027 | 0.000 | 0.019 | 0.000 | 0.006 | 0.008 | 0.000 | 0.000 | 0.000 | 0.000 | 0.000 | 0.000 | 0.000 | 0.000 | 0.000 | 0.000 | 0.000 | 0.000 |
| p__Firmicutes;c__Bacilli;o__Erysipelotrichales;f__Erysipelatoclostridiaceae;g__Candidatus_Stoquefichus | 0.000 | 0.000 | 0.000 | 0.000 | 0.000 | 0.000 | 0.028 | 0.000 | 0.000 | 0.000 | 0.000 | 0.000 | 0.000 | 0.025 | 0.000 | 0.000 | 0.000 | 0.000 |
| p__Firmicutes;c__Bacilli;o__Erysipelotrichales;f__Erysipelatoclostridiaceae;g__Erysipelatoclostridium | 0.061 | 0.021 | 0.042 | 0.019 | 0.060 | 0.053 | 1.563 | 0.028 | 0.452 | 0.997 | 0.371 | 0.257 | 0.474 | 3.259 | 1.749 | 0.793 | 0.486 | 1.533 |
| p__Firmicutes;c__Bacilli;o__Erysipelotrichales;f__Erysipelatoclostridiaceae;g__Erysipelotrichaceae_UCG-003 | 0.000 | 0.000 | 0.010 | 0.000 | 0.006 | 0.000 | 0.000 | 0.005 | 0.004 | 0.000 | 0.000 | 0.000 | 0.000 | 0.000 | 0.000 | 0.000 | 0.000 | 0.000 |
| p__Firmicutes;c__Bacilli;o__Erysipelotrichales;f__Erysipelotrichaceae | 0.020 | 0.045 | 0.011 | 0.035 | 0.000 | 0.000 | 0.196 | 0.016 | 0.003 | 0.213 | 0.000 | 0.311 | 0.097 | 0.546 | 0.009 | 2.628 | 0.217 | 0.000 |
| p__Firmicutes;c__Bacilli;o__Erysipelotrichales;f__Erysipelotrichaceae;g__[Clostridium]_innocuum_group | 0.010 | 0.000 | 0.000 | 0.000 | 0.000 | 0.000 | 0.000 | 0.000 | 0.000 | 0.000 | 0.004 | 0.000 | 0.000 | 0.000 | 0.000 | 0.000 | 0.000 | 0.000 |
| p__Firmicutes;c__Bacilli;o__Erysipelotrichales;f__Erysipelotrichaceae;g__Allobaculum | 0.049 | 0.000 | 0.214 | 0.066 | 0.000 | 0.000 | 2.491 | 0.062 | 0.010 | 0.027 | 0.334 | 6.125 | 0.000 | 0.000 | 0.000 | 0.000 | 0.000 | 0.000 |
| p__Firmicutes;c__Bacilli;o__Erysipelotrichales;f__Erysipelotrichaceae;g__Dubosiella | 5.111 | 0.052 | 2.611 | 14.359 | 0.073 | 8.296 | 0.010 | 0.028 | 0.018 | 0.070 | 0.099 | 0.137 | 0.050 | 0.019 | 0.000 | 0.000 | 0.012 | 0.019 |
| p__Firmicutes;c__Bacilli;o__Erysipelotrichales;f__Erysipelotrichaceae;g__Erysipelothrix | 0.020 | 0.000 | 0.000 | 0.000 | 0.000 | 0.000 | 0.000 | 0.000 | 0.000 | 0.000 | 0.000 | 0.007 | 0.000 | 0.000 | 0.000 | 0.000 | 0.000 | 0.000 |
| p__Firmicutes;c__Bacilli;o__Erysipelotrichales;f__Erysipelotrichaceae;g__Erysipelotrichaceae | 0.004 | 0.021 | 0.000 | 0.000 | 0.006 | 0.000 | 0.348 | 0.000 | 0.102 | 0.112 | 0.038 | 0.060 | 0.076 | 0.106 | 0.091 | 0.203 | 0.054 | 0.147 |
| p__Firmicutes;c__Bacilli;o__Erysipelotrichales;f__Erysipelotrichaceae;g__Faecalibaculum | 0.029 | 0.178 | 0.050 | 0.237 | 0.052 | 0.058 | 3.280 | 0.140 | 0.066 | 0.109 | 0.574 | 4.196 | 0.738 | 7.277 | 0.614 | 1.122 | 2.775 | 9.067 |
| p__Firmicutes;c__Bacilli;o__Erysipelotrichales;f__Erysipelotrichaceae;g__Ileibacterium | 0.000 | 0.017 | 0.018 | 0.000 | 0.024 | 0.023 | 0.013 | 0.023 | 0.022 | 0.012 | 0.011 | 0.005 | 0.004 | 0.007 | 0.000 | 0.000 | 0.006 | 0.019 |
| p__Firmicutes;c__Bacilli;o__Erysipelotrichales;f__Erysipelotrichaceae;g__Turicibacter | 0.037 | 0.000 | 0.143 | 0.027 | 0.047 | 0.008 | 0.000 | 0.000 | 0.000 | 0.000 | 0.002 | 0.008 | 0.000 | 0.000 | 0.000 | 0.000 | 0.000 | 0.015 |
| p__Firmicutes;c__Bacilli;o__Erysipelotrichales;f__Erysipelotrichaceae;g__uncultured | 0.004 | 0.000 | 0.000 | 0.048 | 0.000 | 0.015 | 0.473 | 0.000 | 0.632 | 0.879 | 0.046 | 0.154 | 0.048 | 0.093 | 0.091 | 0.009 | 0.000 | 0.030 |
| p__Firmicutes;c__Bacilli;o__Lactobacillales | 0.000 | 0.000 | 0.000 | 0.000 | 0.000 | 0.000 | 0.072 | 0.000 | 0.000 | 0.015 | 0.000 | 0.014 | 0.010 | 0.010 | 0.007 | 0.015 | 0.023 | 0.020 |
| p__Firmicutes;c__Bacilli;o__Lactobacillales;f__Aerococcaceae;g__Aerococcus | 0.016 | 0.005 | 0.000 | 0.000 | 0.000 | 0.000 | 0.000 | 0.000 | 0.000 | 0.000 | 0.000 | 0.008 | 0.000 | 0.000 | 0.000 | 0.000 | 0.000 | 0.000 |
| p__Firmicutes;c__Bacilli;o__Lactobacillales;f__Carnobacteriaceae;g__Atopostipes | 0.000 | 0.000 | 0.000 | 0.000 | 0.000 | 0.000 | 0.018 | 0.011 | 0.021 | 0.018 | 0.008 | 0.010 | 0.000 | 0.000 | 0.000 | 0.000 | 0.000 | 0.000 |
| p__Firmicutes;c__Bacilli;o__Lactobacillales;f__Enterococcaceae;g__Enterococcus | 0.000 | 0.000 | 0.000 | 0.000 | 0.000 | 0.000 | 0.067 | 0.014 | 0.000 | 0.007 | 0.000 | 0.000 | 0.000 | 0.009 | 0.000 | 0.024 | 0.000 | 0.000 |
| p__Firmicutes;c__Bacilli;o__Lactobacillales;f__Lactobacillaceae;g__Lactobacillus | 10.052 | 20.226 | 2.202 | 3.435 | 14.823 | 7.634 | 3.463 | 1.310 | 7.559 | 4.170 | 1.136 | 10.838 | 0.422 | 6.738 | 0.698 | 0.613 | 0.568 | 0.299 |
| p__Firmicutes;c__Bacilli;o__Lactobacillales;f__Leuconostocaceae;g__Leuconostoc | 0.000 | 0.000 | 0.000 | 0.000 | 0.000 | 0.000 | 0.155 | 0.000 | 0.010 | 0.012 | 0.000 | 0.037 | 0.023 | 0.025 | 0.016 | 0.047 | 0.020 | 0.042 |
| p__Firmicutes;c__Bacilli;o__Lactobacillales;f__Leuconostocaceae;g__Weissella | 0.008 | 0.009 | 0.015 | 0.006 | 0.000 | 0.008 | 0.000 | 0.000 | 0.003 | 0.007 | 0.000 | 0.000 | 0.000 | 0.000 | 0.000 | 0.000 | 0.000 | 0.000 |
| p__Firmicutes;c__Bacilli;o__Lactobacillales;f__Streptococcaceae;g__Lactococcus | 0.000 | 0.005 | 0.000 | 0.000 | 0.000 | 0.006 | 0.305 | 0.009 | 0.025 | 0.091 | 0.017 | 0.059 | 0.028 | 0.033 | 0.029 | 0.111 | 0.056 | 0.172 |
| p__Firmicutes;c__Bacilli;o__Lactobacillales;f__Streptococcaceae;g__Streptococcus | 0.020 | 0.022 | 0.036 | 0.033 | 0.011 | 0.015 | 0.554 | 0.037 | 0.034 | 0.134 | 0.036 | 0.126 | 0.055 | 0.088 | 0.048 | 0.211 | 0.099 | 0.181 |
| p__Firmicutes;c__Bacilli;o__Mycoplasmatales;f__Mycoplasmataceae;g__uncultured | 0.000 | 0.000 | 0.039 | 0.000 | 0.004 | 0.000 | 0.000 | 0.039 | 0.000 | 0.000 | 0.006 | 0.000 | 0.004 | 0.000 | 0.000 | 0.018 | 0.000 | 0.000 |
| p__Firmicutes;c__Bacilli;o__RF39;f__RF39;g__RF39 | 0.086 | 0.000 | 0.201 | 0.233 | 0.291 | 0.166 | 0.000 | 0.000 | 0.000 | 0.029 | 0.000 | 0.020 | 0.000 | 0.000 | 0.000 | 0.000 | 0.000 | 0.000 |
| p__Firmicutes;c__Bacilli;o__Staphylococcales;f__Staphylococcaceae;g__Jeotgalicoccus | 0.000 | 0.000 | 0.000 | 0.000 | 0.000 | 0.000 | 0.004 | 0.005 | 0.000 | 0.015 | 0.000 | 0.007 | 0.000 | 0.000 | 0.000 | 0.000 | 0.000 | 0.000 |
| p__Firmicutes;c__Bacilli;o__Staphylococcales;f__Staphylococcaceae;g__Staphylococcus | 0.016 | 0.000 | 0.000 | 0.014 | 0.000 | 0.028 | 0.108 | 0.000 | 0.058 | 0.435 | 0.046 | 0.157 | 0.011 | 0.004 | 0.000 | 0.000 | 0.000 | 0.000 |
| p__Firmicutes;c__Clostridia | 0.065 | 0.532 | 0.240 | 0.048 | 0.220 | 0.282 | 0.000 | 0.000 | 0.000 | 0.003 | 0.004 | 0.000 | 0.000 | 0.000 | 0.010 | 0.000 | 0.000 | 0.000 |
| p__Firmicutes;c__Clostridia;o__Christensenellales;f__Christensenellaceae | 0.069 | 0.000 | 0.034 | 0.014 | 0.020 | 0.009 | 0.035 | 0.011 | 0.006 | 0.069 | 0.010 | 0.016 | 0.011 | 0.012 | 0.104 | 0.021 | 0.005 | 0.019 |
| p__Firmicutes;c__Clostridia;o__Christensenellales;f__Christensenellaceae;g__Christensenellaceae_R-7_group | 0.049 | 0.021 | 0.073 | 0.014 | 0.013 | 0.041 | 0.845 | 0.060 | 0.136 | 0.534 | 0.349 | 0.064 | 0.117 | 0.043 | 0.117 | 0.078 | 0.084 | 0.145 |
| p__Firmicutes;c__Clostridia;o__Clostridia | 0.000 | 0.000 | 0.003 | 0.008 | 0.000 | 0.000 | 0.000 | 0.004 | 0.006 | 0.013 | 0.012 | 0.014 | 0.014 | 0.000 | 0.000 | 0.011 | 0.000 | 0.000 |
| p__Firmicutes;c__Clostridia;o__Clostridia;f__Hungateiclostridiaceae | 0.000 | 0.000 | 0.000 | 0.000 | 0.013 | 0.000 | 0.000 | 0.000 | 0.000 | 0.000 | 0.000 | 0.000 | 0.002 | 0.000 | 0.000 | 0.000 | 0.000 | 0.000 |
| p__Firmicutes;c__Clostridia;o__Clostridia_UCG-014;f__Clostridia_UCG-014;g__Clostridia_UCG-014 | 0.724 | 0.231 | 2.895 | 1.533 | 0.660 | 1.383 | 0.000 | 0.021 | 0.006 | 0.013 | 0.017 | 0.027 | 0.000 | 0.000 | 0.000 | 0.000 | 0.000 | 0.011 |
| p__Firmicutes;c__Clostridia;o__Clostridia_vadinBB60_group | 0.000 | 0.000 | 0.006 | 0.035 | 0.009 | 0.043 | 0.000 | 0.000 | 0.000 | 0.000 | 0.000 | 0.000 | 0.000 | 0.000 | 0.000 | 0.000 | 0.000 | 0.000 |
| p__Firmicutes;c__Clostridia;o__Clostridiales;f__Clostridiaceae | 0.000 | 0.000 | 0.000 | 0.000 | 0.000 | 0.000 | 0.000 | 0.000 | 0.000 | 0.010 | 0.000 | 0.008 | 0.000 | 0.000 | 0.000 | 0.006 | 0.000 | 0.000 |
| p__Firmicutes;c__Clostridia;o__Clostridiales;f__Clostridiaceae;g__Candidatus_Arthromitus | 0.000 | 0.000 | 0.000 | 0.000 | 0.000 | 0.000 | 0.000 | 0.048 | 0.000 | 0.000 | 0.000 | 0.000 | 0.002 | 0.000 | 0.000 | 0.000 | 0.000 | 0.000 |
| p__Firmicutes;c__Clostridia;o__Clostridiales;f__Clostridiaceae;g__Clostridium_sensu_stricto_1 | 0.000 | 0.000 | 0.000 | 0.000 | 0.000 | 0.008 | 0.014 | 0.000 | 0.012 | 0.000 | 0.012 | 0.000 | 0.000 | 0.004 | 0.000 | 0.000 | 0.015 | 0.004 |
| p__Firmicutes;c__Clostridia;o__Lachnospirales;f__Lachnospiraceae | 9.724 | 16.673 | 13.036 | 7.616 | 12.855 | 10.995 | 26.334 | 39.263 | 34.047 | 21.987 | 23.237 | 17.875 | 26.753 | 10.739 | 7.868 | 23.591 | 9.562 | 17.067 |
| p__Firmicutes;c__Clostridia;o__Lachnospirales;f__Lachnospiraceae;g__[Eubacterium]_hallii_group | 0.169 | 0.000 | 1.042 | 0.149 | 0.095 | 0.335 | 0.000 | 0.000 | 0.000 | 0.000 | 0.000 | 0.000 | 0.000 | 0.000 | 0.000 | 0.000 | 0.000 | 0.000 |
| p__Firmicutes;c__Clostridia;o__Lachnospirales;f__Lachnospiraceae;g__[Eubacterium]_ventriosum_group | 0.000 | 0.000 | 0.000 | 0.000 | 0.000 | 0.008 | 0.000 | 0.012 | 0.034 | 0.027 | 0.049 | 0.010 | 0.000 | 0.013 | 0.000 | 0.000 | 0.023 | 0.000 |
| p__Firmicutes;c__Clostridia;o__Lachnospirales;f__Lachnospiraceae;g__[Eubacterium]_xylanophilum_group | 0.597 | 0.000 | 0.690 | 0.303 | 0.550 | 0.578 | 0.000 | 0.000 | 0.000 | 0.000 | 0.000 | 0.000 | 0.000 | 0.000 | 0.000 | 0.000 | 0.000 | 0.000 |
| p__Firmicutes;c__Clostridia;o__Lachnospirales;f__Lachnospiraceae;g__[Ruminococcus]_gnavus_group | 0.014 | 0.014 | 0.018 | 0.027 | 0.019 | 0.030 | 0.000 | 0.000 | 0.000 | 0.000 | 0.026 | 0.000 | 0.000 | 0.000 | 0.000 | 0.000 | 0.000 | 0.000 |
| p__Firmicutes;c__Clostridia;o__Lachnospirales;f__Lachnospiraceae;g__A2 | 0.000 | 0.000 | 0.010 | 0.035 | 0.000 | 0.000 | 0.067 | 0.475 | 2.961 | 1.536 | 2.895 | 0.231 | 0.184 | 0.187 | 0.765 | 0.033 | 0.000 | 0.572 |
| p__Firmicutes;c__Clostridia;o__Lachnospirales;f__Lachnospiraceae;g__Acetatifactor | 0.132 | 0.033 | 0.047 | 0.000 | 0.054 | 0.030 | 0.000 | 0.034 | 0.038 | 0.000 | 0.074 | 0.011 | 0.008 | 0.000 | 0.000 | 0.000 | 0.000 | 0.000 |
| p__Firmicutes;c__Clostridia;o__Lachnospirales;f__Lachnospiraceae;g__Agathobacter | 0.016 | 0.000 | 0.000 | 0.031 | 0.022 | 0.043 | 0.000 | 0.027 | 0.022 | 0.027 | 0.037 | 0.000 | 0.029 | 0.012 | 0.000 | 0.000 | 0.000 | 0.000 |
| p__Firmicutes;c__Clostridia;o__Lachnospirales;f__Lachnospiraceae;g__ASF356 | 0.000 | 0.000 | 0.073 | 0.000 | 0.000 | 0.019 | 0.000 | 0.000 | 0.000 | 0.000 | 0.000 | 0.000 | 0.000 | 0.000 | 0.000 | 0.000 | 0.000 | 0.000 |
| p__Firmicutes;c__Clostridia;o__Lachnospirales;f__Lachnospiraceae;g__Blautia | 0.373 | 0.218 | 1.276 | 4.720 | 1.088 | 0.395 | 5.178 | 16.593 | 3.713 | 4.107 | 4.273 | 1.624 | 5.936 | 2.687 | 0.345 | 9.854 | 9.091 | 0.659 |
| p__Firmicutes;c__Clostridia;o__Lachnospirales;f__Lachnospiraceae;g__Coprococcus | 0.000 | 0.000 | 0.000 | 0.000 | 0.000 | 0.000 | 0.050 | 0.055 | 0.093 | 0.064 | 0.052 | 0.016 | 0.019 | 0.006 | 0.000 | 0.021 | 0.012 | 0.026 |
| p__Firmicutes;c__Clostridia;o__Lachnospirales;f__Lachnospiraceae;g__Eisenbergiella | 0.000 | 0.000 | 0.000 | 0.000 | 0.000 | 0.000 | 0.000 | 0.000 | 0.000 | 0.000 | 0.018 | 0.000 | 0.017 | 0.000 | 0.000 | 0.000 | 0.000 | 0.000 |
| p__Firmicutes;c__Clostridia;o__Lachnospirales;f__Lachnospiraceae;g__GCA-900066575 | 0.022 | 0.047 | 0.029 | 0.000 | 0.035 | 0.036 | 0.075 | 0.170 | 0.484 | 0.247 | 0.277 | 0.018 | 0.153 | 0.042 | 0.025 | 0.089 | 0.015 | 0.000 |
| p__Firmicutes;c__Clostridia;o__Lachnospirales;f__Lachnospiraceae;g__Lachnoclostridium | 0.622 | 0.432 | 0.526 | 0.191 | 0.684 | 0.805 | 4.694 | 2.837 | 1.296 | 3.083 | 0.531 | 0.385 | 1.256 | 0.430 | 1.764 | 2.629 | 2.752 | 2.063 |
| p__Firmicutes;c__Clostridia;o__Lachnospirales;f__Lachnospiraceae;g__Lachnospira | 0.000 | 0.005 | 0.000 | 0.000 | 0.004 | 0.000 | 0.000 | 0.000 | 0.000 | 0.000 | 0.000 | 0.000 | 0.000 | 0.000 | 0.000 | 0.000 | 0.000 | 0.000 |
| p__Firmicutes;c__Clostridia;o__Lachnospirales;f__Lachnospiraceae;g__Lachnospiraceae_FCS020_group | 0.029 | 1.724 | 0.149 | 0.287 | 0.097 | 0.021 | 0.304 | 3.539 | 0.881 | 0.000 | 0.499 | 1.300 | 6.623 | 1.978 | 2.206 | 3.444 | 7.108 | 3.288 |
| p__Firmicutes;c__Clostridia;o__Lachnospirales;f__Lachnospiraceae;g__Lachnospiraceae_NK4A136_group | 11.712 | 3.992 | 1.619 | 6.579 | 5.914 | 6.966 | 2.315 | 1.643 | 3.793 | 1.418 | 0.518 | 0.090 | 0.274 | 0.034 | 0.381 | 0.377 | 0.217 | 1.926 |
| p__Firmicutes;c__Clostridia;o__Lachnospirales;f__Lachnospiraceae;g__Lachnospiraceae_UCG-004 | 0.340 | 0.482 | 0.292 | 0.208 | 0.307 | 0.329 | 0.054 | 0.110 | 3.263 | 2.000 | 1.104 | 3.299 | 6.096 | 4.272 | 4.366 | 7.007 | 7.505 | 11.711 |
| p__Firmicutes;c__Clostridia;o__Lachnospirales;f__Lachnospiraceae;g__Lachnospiraceae_UCG-006 | 0.157 | 0.187 | 0.562 | 0.098 | 0.110 | 0.621 | 0.713 | 0.548 | 0.215 | 2.173 | 0.223 | 0.138 | 2.283 | 2.545 | 0.842 | 3.285 | 1.124 | 3.210 |
| p__Firmicutes;c__Clostridia;o__Lachnospirales;f__Lachnospiraceae;g__Marvinbryantia | 0.037 | 0.041 | 0.028 | 0.017 | 0.009 | 0.036 | 0.200 | 0.021 | 0.022 | 0.072 | 0.046 | 0.063 | 0.143 | 1.002 | 0.019 | 0.226 | 0.176 | 0.580 |
| p__Firmicutes;c__Clostridia;o__Lachnospirales;f__Lachnospiraceae;g__Roseburia | 0.100 | 0.041 | 0.193 | 0.079 | 0.149 | 0.169 | 0.285 | 1.072 | 0.031 | 1.269 | 0.732 | 0.052 | 0.000 | 0.004 | 0.009 | 0.000 | 0.223 | 0.025 |
| p__Firmicutes;c__Clostridia;o__Lachnospirales;f__Lachnospiraceae;g__Tuzzerella | 0.010 | 0.000 | 0.005 | 0.012 | 0.022 | 0.019 | 0.020 | 0.012 | 0.019 | 0.022 | 0.023 | 0.044 | 0.010 | 0.000 | 0.020 | 0.009 | 0.022 | 0.068 |
| p__Firmicutes;c__Clostridia;o__Lachnospirales;f__Lachnospiraceae;g__Tyzzerella | 0.010 | 0.318 | 0.593 | 0.000 | 0.061 | 0.034 | 0.000 | 0.000 | 0.000 | 0.000 | 0.004 | 0.000 | 0.000 | 0.000 | 0.000 | 0.000 | 0.000 | 0.000 |
| p__Firmicutes;c__Clostridia;o__Lachnospirales;f__Lachnospiraceae;g__UC5-1-2E3 | 0.273 | 0.024 | 0.377 | 0.087 | 0.099 | 0.333 | 0.027 | 0.076 | 0.074 | 0.023 | 0.015 | 0.138 | 0.024 | 0.049 | 0.000 | 0.032 | 0.040 | 0.050 |
| p__Firmicutes;c__Clostridia;o__Monoglobales;f__Monoglobaceae;g__Monoglobus | 0.153 | 0.489 | 0.127 | 0.046 | 0.380 | 0.292 | 0.065 | 0.225 | 0.043 | 0.159 | 0.059 | 0.254 | 0.195 | 0.054 | 0.056 | 0.797 | 0.096 | 0.116 |
| p__Firmicutes;c__Clostridia;o__Oscillospirales | 0.026 | 0.057 | 0.091 | 0.087 | 0.000 | 0.173 | 0.000 | 0.105 | 0.062 | 0.790 | 0.531 | 0.005 | 0.024 | 0.009 | 0.042 | 0.011 | 0.034 | 0.008 |
| p__Firmicutes;c__Clostridia;o__Oscillospirales;f__[Eubacterium]_coprostanoligenes_group | 0.161 | 0.119 | 0.179 | 0.114 | 0.069 | 0.143 | 0.014 | 0.030 | 0.019 | 0.128 | 0.588 | 0.012 | 0.326 | 0.000 | 0.000 | 0.015 | 0.011 | 0.003 |
| p__Firmicutes;c__Clostridia;o__Oscillospirales;f__Butyricicoccaceae;g__Butyricicoccus | 0.302 | 0.530 | 0.175 | 0.154 | 0.373 | 1.121 | 0.024 | 0.080 | 0.000 | 0.198 | 0.120 | 0.015 | 0.179 | 0.009 | 0.013 | 0.282 | 0.062 | 0.050 |
| p__Firmicutes;c__Clostridia;o__Oscillospirales;f__Butyricicoccaceae;g__UCG-009 | 0.000 | 0.010 | 0.000 | 0.006 | 0.007 | 0.000 | 0.000 | 0.000 | 0.000 | 0.000 | 0.000 | 0.000 | 0.000 | 0.000 | 0.000 | 0.000 | 0.000 | 0.000 |
| p__Firmicutes;c__Clostridia;o__Oscillospirales;f__Ethanoligenenaceae;g__Acetanaerobacterium | 0.000 | 0.000 | 0.000 | 0.000 | 0.000 | 0.000 | 0.000 | 0.007 | 0.000 | 0.035 | 0.000 | 0.000 | 0.000 | 0.000 | 0.000 | 0.000 | 0.000 | 0.000 |
| p__Firmicutes;c__Clostridia;o__Oscillospirales;f__Oscillospiraceae | 0.612 | 0.062 | 0.580 | 1.026 | 0.643 | 1.650 | 0.009 | 0.035 | 0.109 | 0.950 | 0.690 | 0.210 | 0.111 | 0.007 | 0.080 | 0.045 | 0.096 | 0.014 |
| p__Firmicutes;c__Clostridia;o__Oscillospirales;f__Oscillospiraceae;g__Colidextribacter | 0.848 | 0.166 | 0.682 | 0.700 | 0.443 | 1.342 | 0.011 | 0.890 | 2.590 | 3.348 | 3.349 | 0.363 | 0.181 | 0.306 | 0.627 | 0.000 | 0.193 | 0.019 |
| p__Firmicutes;c__Clostridia;o__Oscillospirales;f__Oscillospiraceae;g__Flavonifractor | 0.000 | 0.000 | 0.000 | 0.000 | 0.000 | 0.000 | 0.034 | 0.000 | 0.000 | 0.151 | 0.013 | 0.000 | 0.000 | 0.000 | 0.013 | 0.000 | 0.000 | 0.000 |
| p__Firmicutes;c__Clostridia;o__Oscillospirales;f__Oscillospiraceae;g__Intestinimonas | 0.186 | 0.155 | 0.187 | 0.183 | 0.151 | 0.162 | 0.034 | 0.027 | 0.038 | 0.094 | 0.008 | 0.098 | 0.426 | 0.096 | 0.051 | 0.538 | 0.254 | 0.479 |
| p__Firmicutes;c__Clostridia;o__Oscillospirales;f__Oscillospiraceae;g__NK4A214_group | 0.075 | 0.086 | 0.196 | 0.031 | 0.078 | 0.043 | 0.000 | 0.000 | 0.000 | 0.000 | 0.000 | 0.000 | 0.000 | 0.000 | 0.000 | 0.000 | 0.000 | 0.000 |
| p__Firmicutes;c__Clostridia;o__Oscillospirales;f__Oscillospiraceae;g__Oscillibacter | 0.379 | 0.086 | 0.492 | 1.132 | 0.781 | 1.321 | 0.009 | 0.211 | 0.239 | 0.123 | 0.059 | 0.014 | 0.068 | 0.009 | 0.088 | 0.018 | 0.045 | 0.040 |
| p__Firmicutes;c__Clostridia;o__Oscillospirales;f__Oscillospiraceae;g__UCG-005 | 0.000 | 0.010 | 0.013 | 0.000 | 0.000 | 0.030 | 0.000 | 0.000 | 0.000 | 0.000 | 0.000 | 0.000 | 0.000 | 0.000 | 0.000 | 0.000 | 0.000 | 0.000 |
| p__Firmicutes;c__Clostridia;o__Oscillospirales;f__Oscillospiraceae;g__uncultured | 0.385 | 0.143 | 0.784 | 0.318 | 0.797 | 0.958 | 0.000 | 0.000 | 0.016 | 0.183 | 0.037 | 0.000 | 0.000 | 0.000 | 0.081 | 0.000 | 0.000 | 0.000 |
| p__Firmicutes;c__Clostridia;o__Oscillospirales;f__Oscillospirales;g__Hydrogenoanaerobacterium | 0.008 | 0.000 | 0.000 | 0.000 | 0.006 | 0.000 | 0.000 | 0.000 | 0.000 | 0.000 | 0.000 | 0.000 | 0.000 | 0.000 | 0.000 | 0.000 | 0.000 | 0.000 |
| p__Firmicutes;c__Clostridia;o__Oscillospirales;f__Ruminococcaceae | 0.022 | 0.041 | 0.006 | 0.075 | 0.073 | 0.132 | 1.424 | 0.307 | 0.592 | 1.564 | 0.274 | 0.654 | 0.516 | 0.809 | 0.459 | 0.910 | 0.683 | 0.764 |
| p__Firmicutes;c__Clostridia;o__Oscillospirales;f__Ruminococcaceae;g__[Eubacterium]_siraeum_group | 0.442 | 0.269 | 0.815 | 0.457 | 0.596 | 0.822 | 0.000 | 0.011 | 0.000 | 0.000 | 0.000 | 0.000 | 0.004 | 0.000 | 0.000 | 0.006 | 0.003 | 0.000 |
| p__Firmicutes;c__Clostridia;o__Oscillospirales;f__Ruminococcaceae;g__Anaerotruncus | 0.139 | 0.190 | 0.484 | 0.125 | 0.639 | 1.174 | 0.301 | 0.610 | 0.456 | 0.906 | 0.552 | 0.384 | 1.973 | 0.142 | 4.156 | 4.556 | 1.132 | 1.697 |
| p__Firmicutes;c__Clostridia;o__Oscillospirales;f__Ruminococcaceae;g__Angelakisella | 0.008 | 0.009 | 0.031 | 0.014 | 0.007 | 0.024 | 0.000 | 0.000 | 0.000 | 0.000 | 0.578 | 0.022 | 0.000 | 0.012 | 0.029 | 0.006 | 0.000 | 0.000 |
| p__Firmicutes;c__Clostridia;o__Oscillospirales;f__Ruminococcaceae;g__Candidatus_Soleaferrea | 0.016 | 0.000 | 0.003 | 0.000 | 0.007 | 0.006 | 0.000 | 0.009 | 0.000 | 0.020 | 0.000 | 0.000 | 0.006 | 0.015 | 0.000 | 0.021 | 0.009 | 0.026 |
| p__Firmicutes;c__Clostridia;o__Oscillospirales;f__Ruminococcaceae;g__Faecalibacterium | 0.024 | 0.043 | 0.058 | 0.046 | 0.058 | 0.055 | 0.068 | 0.067 | 0.050 | 0.081 | 0.043 | 0.033 | 0.030 | 0.000 | 0.000 | 0.024 | 0.023 | 0.040 |
| p__Firmicutes;c__Clostridia;o__Oscillospirales;f__Ruminococcaceae;g__Harryflintia | 0.000 | 0.000 | 0.000 | 0.000 | 0.000 | 0.000 | 0.000 | 0.058 | 0.000 | 0.000 | 0.023 | 0.000 | 0.000 | 0.000 | 0.000 | 0.000 | 0.000 | 0.000 |
| p__Firmicutes;c__Clostridia;o__Oscillospirales;f__Ruminococcaceae;g__Incertae_Sedis | 0.677 | 0.216 | 0.627 | 0.191 | 0.212 | 1.082 | 0.014 | 0.441 | 0.353 | 1.999 | 0.829 | 0.025 | 0.226 | 0.015 | 0.900 | 0.000 | 0.068 | 0.015 |
| p__Firmicutes;c__Clostridia;o__Oscillospirales;f__Ruminococcaceae;g__Negativibacillus | 0.000 | 0.000 | 0.010 | 0.000 | 0.039 | 0.070 | 0.000 | 0.021 | 0.035 | 0.086 | 0.026 | 0.010 | 0.000 | 0.000 | 0.014 | 0.000 | 0.023 | 0.011 |
| p__Firmicutes;c__Clostridia;o__Oscillospirales;f__Ruminococcaceae;g__Ruminococcus | 0.039 | 0.054 | 0.106 | 0.021 | 0.065 | 0.000 | 0.000 | 0.000 | 0.000 | 0.000 | 0.000 | 0.000 | 0.000 | 0.000 | 0.000 | 0.000 | 0.000 | 0.000 |
| p__Firmicutes;c__Clostridia;o__Oscillospirales;f__Ruminococcaceae;g__Subdoligranulum | 0.000 | 0.000 | 0.000 | 0.000 | 0.006 | 0.000 | 0.000 | 0.000 | 0.000 | 0.000 | 0.000 | 0.000 | 0.007 | 0.000 | 0.000 | 0.000 | 0.000 | 0.000 |
| p__Firmicutes;c__Clostridia;o__Oscillospirales;f__Ruminococcaceae;g__UBA1819 | 0.000 | 0.000 | 0.000 | 0.000 | 0.000 | 0.000 | 0.000 | 0.000 | 0.000 | 0.000 | 0.000 | 0.000 | 0.015 | 0.069 | 0.510 | 0.000 | 0.000 | 0.000 |
| p__Firmicutes;c__Clostridia;o__Oscillospirales;f__Ruminococcaceae;g__uncultured | 0.014 | 0.000 | 0.013 | 0.000 | 0.000 | 0.009 | 0.000 | 0.000 | 0.000 | 0.000 | 0.000 | 0.000 | 0.000 | 0.000 | 0.000 | 0.000 | 0.000 | 0.000 |
| p__Firmicutes;c__Clostridia;o__Oscillospirales;f__UCG-010;g__UCG-010 | 0.029 | 0.003 | 0.015 | 0.023 | 0.022 | 0.004 | 0.000 | 0.000 | 0.000 | 0.000 | 0.000 | 0.000 | 0.003 | 0.000 | 0.000 | 0.000 | 0.000 | 0.000 |
| p__Firmicutes;c__Clostridia;o__Peptococcales;f__Peptococcaceae;g__uncultured | 0.608 | 0.375 | 1.114 | 0.731 | 0.639 | 1.061 | 0.202 | 0.126 | 0.596 | 0.592 | 0.499 | 0.781 | 0.287 | 0.711 | 0.252 | 0.318 | 0.488 | 0.065 |
| p__Firmicutes;c__Clostridia;o__Peptostreptococcales-Tissierellales | 0.000 | 0.000 | 0.003 | 0.000 | 0.000 | 0.000 | 0.013 | 0.000 | 0.022 | 0.000 | 0.014 | 0.004 | 0.011 | 0.015 | 0.023 | 0.014 | 0.008 | 0.000 |
| p__Firmicutes;c__Clostridia;o__Peptostreptococcales-Tissierellales;f__Anaerovoracaceae | 0.110 | 0.083 | 0.123 | 0.068 | 0.076 | 0.088 | 0.040 | 0.028 | 0.021 | 0.128 | 0.027 | 0.000 | 0.006 | 0.000 | 0.083 | 0.000 | 0.000 | 0.014 |
| p__Firmicutes;c__Clostridia;o__Peptostreptococcales-Tissierellales;f__Anaerovoracaceae;g__[Eubacterium]_brachy_group | 0.027 | 0.000 | 0.016 | 0.021 | 0.013 | 0.011 | 0.000 | 0.000 | 0.000 | 0.000 | 0.000 | 0.014 | 0.000 | 0.016 | 0.000 | 0.030 | 0.000 | 0.000 |
| p__Firmicutes;c__Clostridia;o__Peptostreptococcales-Tissierellales;f__Anaerovoracaceae;g__[Eubacterium]_nodatum_group | 0.094 | 0.019 | 0.127 | 0.033 | 0.080 | 0.070 | 0.000 | 0.000 | 0.000 | 0.000 | 0.000 | 0.000 | 0.000 | 0.000 | 0.000 | 0.014 | 0.000 | 0.000 |
| p__Firmicutes;c__Clostridia;o__Peptostreptococcales-Tissierellales;f__Anaerovoracaceae;g__Family_XIII_AD3011_group | 0.094 | 0.083 | 0.140 | 0.027 | 0.097 | 0.107 | 0.028 | 0.037 | 0.022 | 0.119 | 0.037 | 0.070 | 0.175 | 0.073 | 0.201 | 0.244 | 0.059 | 0.097 |
| p__Firmicutes;c__Clostridia;o__Peptostreptococcales-Tissierellales;f__Peptostreptococcaceae | 0.033 | 0.016 | 0.041 | 0.025 | 0.000 | 0.000 | 0.461 | 0.115 | 0.704 | 0.170 | 0.190 | 0.026 | 0.546 | 1.282 | 0.293 | 0.292 | 0.211 | 1.428 |
| p__Firmicutes;c__Clostridia;o__Peptostreptococcales-Tissierellales;f__Peptostreptococcaceae;g__Clostridioides | 0.000 | 0.000 | 0.000 | 0.000 | 0.000 | 0.000 | 0.000 | 0.007 | 0.000 | 0.000 | 0.007 | 0.007 | 0.000 | 0.000 | 0.000 | 0.000 | 0.000 | 0.000 |
| p__Firmicutes;c__Clostridia;o__Peptostreptococcales-Tissierellales;f__Peptostreptococcaceae;g__Proteocatella | 0.000 | 0.000 | 0.000 | 0.000 | 0.000 | 0.000 | 0.000 | 0.019 | 0.000 | 0.005 | 0.000 | 0.000 | 0.000 | 0.000 | 0.000 | 0.000 | 0.000 | 0.000 |
| p__Firmicutes;c__Clostridia;o__Peptostreptococcales-Tissierellales;f__Peptostreptococcaceae;g__uncultured | 0.000 | 0.000 | 0.000 | 0.000 | 0.022 | 0.009 | 0.186 | 0.000 | 0.187 | 0.065 | 0.054 | 0.000 | 0.157 | 0.399 | 0.080 | 0.142 | 0.000 | 0.412 |
| p__Firmicutes;c__Negativicutes;o__Veillonellales-Selenomonadales;f__Veillonellaceae;g__Dialister | 0.000 | 0.000 | 0.000 | 0.000 | 0.000 | 0.008 | 0.000 | 0.000 | 0.007 | 0.000 | 0.007 | 0.000 | 0.000 | 0.000 | 0.000 | 0.000 | 0.000 | 0.005 |
| p__Firmicutes;c__Negativicutes;o__Veillonellales-Selenomonadales;f__Veillonellaceae;g__Megasphaera | 0.000 | 0.000 | 0.000 | 0.000 | 0.000 | 0.011 | 0.000 | 0.000 | 0.007 | 0.000 | 0.000 | 0.005 | 0.000 | 0.000 | 0.000 | 0.000 | 0.005 | 0.000 |
| p__Fusobacteriota;c__Fusobacteriia;o__Fusobacteriales;f__Fusobacteriaceae;g__Cetobacterium | 0.045 | 0.000 | 0.018 | 0.000 | 0.000 | 0.032 | 0.000 | 0.108 | 0.007 | 0.060 | 0.067 | 0.040 | 0.033 | 0.024 | 0.000 | 0.012 | 0.011 | 0.027 |
| p__GAL15;c__GAL15;o__GAL15;f__GAL15;g__GAL15 | 0.000 | 0.000 | 0.000 | 0.000 | 0.000 | 0.000 | 0.000 | 0.000 | 0.000 | 0.000 | 0.450 | 0.000 | 0.114 | 0.000 | 0.000 | 0.000 | 0.000 | 0.000 |
| p__Gemmatimonadota;c__Gemmatimonadetes;o__Gemmatimonadales;f__Gemmatimonadaceae;g__uncultured | 0.010 | 0.000 | 0.000 | 0.000 | 0.013 | 0.006 | 0.000 | 0.000 | 0.000 | 0.000 | 0.000 | 0.000 | 0.000 | 0.000 | 0.000 | 0.000 | 0.000 | 0.000 |
| p__Myxococcota;c__Polyangia;o__Haliangiales;f__Haliangiaceae;g__Haliangium | 0.000 | 0.000 | 0.002 | 0.000 | 0.004 | 0.000 | 0.000 | 0.000 | 0.000 | 0.000 | 0.000 | 0.000 | 0.000 | 0.000 | 0.000 | 0.000 | 0.000 | 0.000 |
| p__Myxococcota;c__Polyangia;o__Polyangiales;f__Phaselicystidaceae;g__Phaselicystis | 0.000 | 0.007 | 0.000 | 0.008 | 0.000 | 0.000 | 0.000 | 0.000 | 0.000 | 0.007 | 0.006 | 0.000 | 0.000 | 0.000 | 0.000 | 0.000 | 0.000 | 0.000 |
| p__Nitrospirota;c__Nitrospiria;o__Nitrospirales;f__Nitrospiraceae;g__Nitrospira | 0.010 | 0.012 | 0.000 | 0.012 | 0.019 | 0.011 | 0.010 | 0.007 | 0.000 | 0.017 | 0.027 | 0.014 | 0.000 | 0.000 | 0.000 | 0.000 | 0.000 | 0.000 |
| p__Patescibacteria;c__Saccharimonadia;o__Saccharimonadales | 0.000 | 0.000 | 0.000 | 0.000 | 0.000 | 0.000 | 0.000 | 0.004 | 0.006 | 0.000 | 0.000 | 0.000 | 0.000 | 0.000 | 0.000 | 0.000 | 0.000 | 0.000 |
| p__Patescibacteria;c__Saccharimonadia;o__Saccharimonadales;f__Saccharimonadaceae;g__Candidatus_Saccharimonas | 2.948 | 1.557 | 4.007 | 5.113 | 1.917 | 4.633 | 0.009 | 0.000 | 0.009 | 0.054 | 0.039 | 0.083 | 0.006 | 0.004 | 0.000 | 0.014 | 0.008 | 0.007 |
| p__Patescibacteria;c__Saccharimonadia;o__Saccharimonadales;f__Saccharimonadaceae;g__TM7a | 0.000 | 0.000 | 0.000 | 0.000 | 0.000 | 0.000 | 0.000 | 0.007 | 0.006 | 0.000 | 0.000 | 0.005 | 0.000 | 0.000 | 0.000 | 0.000 | 0.000 | 0.000 |
| p__Planctomycetota;c__Brocadiae;o__Brocadiales;f__Scalinduaceae;g__Candidatus_Scalindua | 0.010 | 0.003 | 0.000 | 0.000 | 0.000 | 0.000 | 0.003 | 0.000 | 0.000 | 0.000 | 0.000 | 0.007 | 0.000 | 0.003 | 0.000 | 0.000 | 0.000 | 0.000 |
| p__Proteobacteria;c__Alphaproteobacteria;o__Acetobacterales;f__Acetobacteraceae;g__uncultured | 0.000 | 0.000 | 0.000 | 0.000 | 0.000 | 0.000 | 0.000 | 0.000 | 0.000 | 0.003 | 0.000 | 0.000 | 0.000 | 0.003 | 0.000 | 0.000 | 0.000 | 0.000 |
| p__Proteobacteria;c__Alphaproteobacteria;o__Azospirillales;f__uncultured;g__uncultured | 0.006 | 0.000 | 0.000 | 0.000 | 0.006 | 0.000 | 0.000 | 0.027 | 0.000 | 0.000 | 0.000 | 0.000 | 0.000 | 0.000 | 0.000 | 0.000 | 0.000 | 0.000 |
| p__Proteobacteria;c__Alphaproteobacteria;o__Caulobacterales;f__Hyphomonadaceae;g__Hellea | 0.000 | 0.000 | 0.000 | 0.000 | 0.000 | 0.000 | 0.000 | 0.018 | 0.000 | 0.029 | 0.000 | 0.000 | 0.000 | 0.000 | 0.000 | 0.000 | 0.000 | 0.000 |
| p__Proteobacteria;c__Alphaproteobacteria;o__Caulobacterales;f__Hyphomonadaceae;g__Litorimonas | 0.000 | 0.000 | 0.000 | 0.000 | 0.000 | 0.000 | 0.000 | 0.014 | 0.000 | 0.013 | 0.000 | 0.000 | 0.000 | 0.000 | 0.000 | 0.000 | 0.000 | 0.000 |
| p__Proteobacteria;c__Alphaproteobacteria;o__Rhizobiales;f__D05-2;g__D05-2 | 0.000 | 0.007 | 0.000 | 0.000 | 0.007 | 0.000 | 0.000 | 0.000 | 0.000 | 0.007 | 0.004 | 0.000 | 0.000 | 0.000 | 0.000 | 0.000 | 0.000 | 0.000 |
| p__Proteobacteria;c__Alphaproteobacteria;o__Rhizobiales;f__Rhizobiaceae | 0.020 | 0.017 | 0.010 | 0.012 | 0.000 | 0.000 | 0.020 | 0.035 | 0.000 | 0.012 | 0.011 | 0.011 | 0.044 | 0.000 | 0.000 | 0.000 | 0.005 | 0.016 |
| p__Proteobacteria;c__Alphaproteobacteria;o__Rhizobiales;f__Rhizobiaceae;g__Aliihoeflea | 0.000 | 0.000 | 0.000 | 0.000 | 0.000 | 0.000 | 0.000 | 0.016 | 0.000 | 0.000 | 0.000 | 0.000 | 0.013 | 0.000 | 0.000 | 0.000 | 0.000 | 0.000 |
| p__Proteobacteria;c__Alphaproteobacteria;o__Rhizobiales;f__Rhizobiales_Incertae_Sedis;g__uncultured | 0.012 | 0.007 | 0.000 | 0.000 | 0.000 | 0.000 | 0.000 | 0.000 | 0.000 | 0.000 | 0.000 | 0.000 | 0.000 | 0.000 | 0.000 | 0.000 | 0.000 | 0.000 |
| p__Proteobacteria;c__Alphaproteobacteria;o__Rhizobiales;f__Xanthobacteraceae | 0.000 | 0.000 | 0.000 | 0.000 | 0.000 | 0.000 | 0.030 | 0.021 | 0.025 | 0.013 | 0.000 | 0.000 | 0.000 | 0.000 | 0.000 | 0.000 | 0.000 | 0.000 |
| p__Proteobacteria;c__Alphaproteobacteria;o__Rhodobacterales;f__Rhodobacteraceae | 0.059 | 0.041 | 0.052 | 0.029 | 0.017 | 0.045 | 0.033 | 0.385 | 0.043 | 0.282 | 0.039 | 0.019 | 0.012 | 0.018 | 0.000 | 0.041 | 0.009 | 0.010 |
| p__Proteobacteria;c__Alphaproteobacteria;o__Rhodobacterales;f__Rhodobacteraceae;g__Halocynthiibacter | 0.079 | 0.024 | 0.039 | 0.029 | 0.035 | 0.030 | 0.014 | 0.037 | 0.027 | 0.012 | 0.037 | 0.037 | 0.021 | 0.024 | 0.012 | 0.023 | 0.015 | 0.012 |
| p__Proteobacteria;c__Alphaproteobacteria;o__Rhodobacterales;f__Rhodobacteraceae;g__Pseudoruegeria | 0.000 | 0.010 | 0.000 | 0.015 | 0.000 | 0.000 | 0.000 | 0.000 | 0.000 | 0.000 | 0.015 | 0.010 | 0.000 | 0.000 | 0.012 | 0.000 | 0.000 | 0.000 |
| p__Proteobacteria;c__Alphaproteobacteria;o__Rhodobacterales;f__Rhodobacteraceae;g__Shimia | 0.000 | 0.000 | 0.008 | 0.000 | 0.000 | 0.000 | 0.000 | 0.018 | 0.000 | 0.012 | 0.000 | 0.000 | 0.000 | 0.000 | 0.000 | 0.000 | 0.000 | 0.000 |
| p__Proteobacteria;c__Alphaproteobacteria;o__Rhodobacterales;f__Rhodobacteraceae;g__Yoonia-Loktanella | 0.000 | 0.000 | 0.000 | 0.000 | 0.000 | 0.000 | 0.000 | 0.035 | 0.000 | 0.055 | 0.000 | 0.000 | 0.000 | 0.000 | 0.000 | 0.000 | 0.000 | 0.000 |
| p__Proteobacteria;c__Alphaproteobacteria;o__Rhodospirillales;f__uncultured;g__uncultured | 0.000 | 0.000 | 0.016 | 0.033 | 0.000 | 0.000 | 0.000 | 0.007 | 0.009 | 0.000 | 0.011 | 0.000 | 0.000 | 0.000 | 0.000 | 0.000 | 0.000 | 0.000 |
| p__Proteobacteria;c__Alphaproteobacteria;o__Rickettsiales;f__Mitochondria;g__Mitochondria | 0.033 | 0.019 | 0.016 | 0.052 | 0.047 | 0.038 | 0.013 | 0.019 | 0.032 | 0.035 | 0.035 | 0.004 | 0.011 | 0.000 | 0.000 | 0.000 | 0.017 | 0.014 |
| p__Proteobacteria;c__Alphaproteobacteria;o__Sphingomonadales;f__Sphingomonadaceae | 0.000 | 0.021 | 0.023 | 0.033 | 0.000 | 0.024 | 0.000 | 0.016 | 0.000 | 0.032 | 0.015 | 0.000 | 0.000 | 0.000 | 0.000 | 0.000 | 0.000 | 0.000 |
| p__Proteobacteria;c__Alphaproteobacteria;o__Sphingomonadales;f__Sphingomonadaceae;g__Sphingomonas | 0.079 | 0.038 | 0.041 | 0.025 | 0.065 | 0.064 | 0.065 | 0.025 | 0.041 | 0.060 | 0.055 | 0.020 | 0.000 | 0.000 | 0.000 | 0.030 | 0.039 | 0.016 |
| p__Proteobacteria;c__Alphaproteobacteria;o__Tistrellales;f__Geminicoccaceae | 0.000 | 0.000 | 0.000 | 0.000 | 0.000 | 0.000 | 0.000 | 0.009 | 0.000 | 0.044 | 0.000 | 0.000 | 0.000 | 0.000 | 0.000 | 0.000 | 0.000 | 0.000 |
| p__Proteobacteria;c__Gammaproteobacteria | 0.069 | 0.045 | 0.000 | 0.041 | 0.000 | 0.036 | 0.034 | 0.019 | 0.022 | 0.044 | 0.017 | 0.027 | 0.010 | 0.018 | 0.014 | 0.005 | 0.000 | 0.000 |
| p__Proteobacteria;c__Gammaproteobacteria;o__Alteromonadales;f__Alteromonadaceae | 0.000 | 0.000 | 0.000 | 0.000 | 0.000 | 0.000 | 0.000 | 0.039 | 0.000 | 0.112 | 0.000 | 0.000 | 0.000 | 0.000 | 0.000 | 0.000 | 0.000 | 0.000 |
| p__Proteobacteria;c__Gammaproteobacteria;o__Alteromonadales;f__Pseudoalteromonadaceae;g__Pseudoalteromonas | 0.020 | 0.016 | 0.016 | 0.014 | 0.020 | 0.000 | 0.006 | 0.083 | 0.009 | 0.106 | 0.019 | 0.014 | 0.007 | 0.000 | 0.000 | 0.003 | 0.008 | 0.000 |
| p__Proteobacteria;c__Gammaproteobacteria;o__Alteromonadales;f__Psychromonadaceae;g__Psychromonadaceae | 0.000 | 0.007 | 0.000 | 0.000 | 0.000 | 0.000 | 0.000 | 0.000 | 0.004 | 0.000 | 0.000 | 0.000 | 0.000 | 0.000 | 0.000 | 0.000 | 0.000 | 0.000 |
| p__Proteobacteria;c__Gammaproteobacteria;o__Alteromonadales;f__Shewanellaceae;g__Shewanella | 0.018 | 0.000 | 0.000 | 0.000 | 0.000 | 0.006 | 0.000 | 0.000 | 0.009 | 0.000 | 0.000 | 0.015 | 0.000 | 0.000 | 0.000 | 0.000 | 0.000 | 0.000 |
| p__Proteobacteria;c__Gammaproteobacteria;o__Burkholderiales | 0.041 | 0.059 | 0.029 | 0.044 | 0.117 | 0.011 | 1.509 | 0.062 | 0.906 | 0.915 | 0.252 | 0.255 | 0.003 | 0.099 | 0.000 | 0.145 | 0.133 | 1.056 |
| p__Proteobacteria;c__Gammaproteobacteria;o__Burkholderiales;f__Alcaligenaceae | 0.014 | 0.007 | 0.000 | 0.002 | 0.000 | 0.006 | 0.000 | 0.000 | 0.000 | 0.003 | 0.000 | 0.000 | 0.000 | 0.000 | 0.000 | 0.000 | 0.000 | 0.000 |
| p__Proteobacteria;c__Gammaproteobacteria;o__Burkholderiales;f__Burkholderiaceae;g__Burkholderia-Caballeronia-Paraburkholderia | 0.000 | 0.000 | 0.000 | 0.000 | 0.000 | 0.000 | 0.000 | 0.000 | 0.006 | 0.000 | 0.000 | 0.007 | 0.000 | 0.000 | 0.000 | 0.000 | 0.000 | 0.000 |
| p__Proteobacteria;c__Gammaproteobacteria;o__Burkholderiales;f__Burkholderiaceae;g__Ralstonia | 0.000 | 0.000 | 0.000 | 0.000 | 0.000 | 0.000 | 0.021 | 0.000 | 0.016 | 0.000 | 0.010 | 0.010 | 0.000 | 0.000 | 0.000 | 0.000 | 0.000 | 0.000 |
| p__Proteobacteria;c__Gammaproteobacteria;o__Burkholderiales;f__Comamonadaceae | 0.010 | 0.012 | 0.000 | 0.010 | 0.000 | 0.000 | 0.013 | 0.000 | 0.000 | 0.000 | 0.011 | 0.008 | 0.000 | 0.000 | 0.000 | 0.000 | 0.000 | 0.000 |
| p__Proteobacteria;c__Gammaproteobacteria;o__Burkholderiales;f__Comamonadaceae;g__Comamonas | 0.016 | 0.016 | 0.016 | 0.012 | 0.011 | 0.006 | 0.010 | 0.000 | 0.013 | 0.007 | 0.000 | 0.000 | 0.000 | 0.000 | 0.000 | 0.000 | 0.000 | 0.000 |
| p__Proteobacteria;c__Gammaproteobacteria;o__Burkholderiales;f__Comamonadaceae;g__Hydrogenophaga | 0.000 | 0.000 | 0.000 | 0.000 | 0.000 | 0.000 | 0.000 | 0.000 | 0.000 | 0.000 | 0.007 | 0.000 | 0.008 | 0.000 | 0.000 | 0.000 | 0.000 | 0.000 |
| p__Proteobacteria;c__Gammaproteobacteria;o__Burkholderiales;f__Methylophilaceae;g__Methylotenera | 0.000 | 0.007 | 0.000 | 0.000 | 0.000 | 0.000 | 0.000 | 0.000 | 0.000 | 0.000 | 0.006 | 0.000 | 0.000 | 0.000 | 0.000 | 0.000 | 0.000 | 0.000 |
| p__Proteobacteria;c__Gammaproteobacteria;o__Burkholderiales;f__Nitrosomonadaceae;g__Ellin6067 | 0.002 | 0.009 | 0.000 | 0.000 | 0.007 | 0.000 | 0.000 | 0.000 | 0.000 | 0.005 | 0.000 | 0.000 | 0.000 | 0.000 | 0.000 | 0.000 | 0.000 | 0.000 |
| p__Proteobacteria;c__Gammaproteobacteria;o__Burkholderiales;f__Oxalobacteraceae | 0.039 | 0.033 | 0.015 | 0.041 | 0.028 | 0.019 | 0.006 | 0.004 | 0.009 | 0.025 | 0.023 | 0.000 | 0.000 | 0.000 | 0.000 | 0.000 | 0.000 | 0.000 |
| p__Proteobacteria;c__Gammaproteobacteria;o__Burkholderiales;f__Oxalobacteraceae;g__Massilia | 0.000 | 0.000 | 0.005 | 0.000 | 0.000 | 0.000 | 0.000 | 0.000 | 0.004 | 0.000 | 0.010 | 0.000 | 0.000 | 0.000 | 0.000 | 0.000 | 0.000 | 0.000 |
| p__Proteobacteria;c__Gammaproteobacteria;o__Burkholderiales;f__Oxalobacteraceae;g__Noviherbaspirillum | 0.024 | 0.009 | 0.013 | 0.019 | 0.013 | 0.026 | 0.000 | 0.000 | 0.000 | 0.017 | 0.013 | 0.000 | 0.000 | 0.000 | 0.000 | 0.000 | 0.000 | 0.000 |
| p__Proteobacteria;c__Gammaproteobacteria;o__Burkholderiales;f__Oxalobacteraceae;g__Undibacterium | 0.000 | 0.000 | 0.000 | 0.000 | 0.000 | 0.000 | 0.004 | 0.007 | 0.000 | 0.000 | 0.000 | 0.000 | 0.000 | 0.000 | 0.000 | 0.000 | 0.000 | 0.000 |
| p__Proteobacteria;c__Gammaproteobacteria;o__Burkholderiales;f__Sutterellaceae | 1.301 | 0.916 | 0.826 | 1.300 | 1.269 | 0.894 | 0.030 | 0.016 | 0.285 | 0.020 | 0.148 | 0.307 | 0.051 | 1.984 | 0.190 | 0.012 | 0.015 | 0.012 |
| p__Proteobacteria;c__Gammaproteobacteria;o__Burkholderiales;f__Sutterellaceae;g__Parasutterella | 0.043 | 0.002 | 0.013 | 0.010 | 0.028 | 0.034 | 0.043 | 0.025 | 0.040 | 0.148 | 0.121 | 0.191 | 0.038 | 0.006 | 0.071 | 0.005 | 0.751 | 0.060 |
| p__Proteobacteria;c__Gammaproteobacteria;o__Burkholderiales;f__Sutterellaceae;g__uncultured | 0.000 | 0.000 | 0.000 | 0.000 | 0.000 | 0.000 | 0.000 | 0.000 | 0.000 | 0.008 | 0.010 | 0.000 | 0.000 | 0.000 | 0.000 | 0.000 | 0.000 | 0.000 |
| p__Proteobacteria;c__Gammaproteobacteria;o__CCD24;f__CCD24;g__CCD24 | 0.000 | 0.000 | 0.000 | 0.010 | 0.000 | 0.006 | 0.000 | 0.000 | 0.000 | 0.000 | 0.000 | 0.000 | 0.000 | 0.000 | 0.000 | 0.000 | 0.000 | 0.000 |
| p__Proteobacteria;c__Gammaproteobacteria;o__Cellvibrionales;f__Cellvibrionaceae | 0.006 | 0.000 | 0.010 | 0.000 | 0.000 | 0.000 | 0.000 | 0.000 | 0.000 | 0.000 | 0.007 | 0.000 | 0.000 | 0.000 | 0.000 | 0.000 | 0.000 | 0.000 |
| p__Proteobacteria;c__Gammaproteobacteria;o__Cellvibrionales;f__Halieaceae | 0.000 | 0.000 | 0.000 | 0.000 | 0.000 | 0.000 | 0.004 | 0.000 | 0.000 | 0.000 | 0.000 | 0.000 | 0.000 | 0.003 | 0.000 | 0.000 | 0.000 | 0.000 |
| p__Proteobacteria;c__Gammaproteobacteria;o__Cellvibrionales;f__Halieaceae;g__Halioglobus | 0.000 | 0.000 | 0.010 | 0.008 | 0.007 | 0.000 | 0.000 | 0.000 | 0.000 | 0.000 | 0.008 | 0.000 | 0.000 | 0.000 | 0.000 | 0.000 | 0.000 | 0.000 |
| p__Proteobacteria;c__Gammaproteobacteria;o__Enterobacterales | 0.345 | 0.254 | 0.192 | 0.262 | 0.281 | 0.237 | 2.490 | 0.714 | 1.224 | 2.027 | 1.972 | 5.774 | 0.061 | 0.070 | 0.035 | 0.089 | 0.087 | 0.094 |
| p__Proteobacteria;c__Gammaproteobacteria;o__Enterobacterales;f__Morganellaceae;g__Proteus | 0.000 | 0.000 | 0.000 | 0.000 | 0.000 | 0.000 | 0.000 | 0.000 | 0.000 | 0.000 | 0.020 | 0.000 | 0.008 | 0.000 | 0.000 | 0.000 | 0.000 | 0.000 |
| p__Proteobacteria;c__Gammaproteobacteria;o__Enterobacterales;f__Pectobacteriaceae;g__Dickeya | 0.000 | 0.000 | 0.000 | 0.000 | 0.011 | 0.000 | 0.023 | 0.023 | 0.000 | 0.000 | 0.012 | 0.007 | 0.000 | 0.000 | 0.000 | 0.000 | 0.000 | 0.011 |
| p__Proteobacteria;c__Gammaproteobacteria;o__Gammaproteobacteria_Incertae_Sedis;f__Unknown_Family;g__uncultured | 0.000 | 0.012 | 0.008 | 0.000 | 0.000 | 0.000 | 0.011 | 0.005 | 0.000 | 0.000 | 0.005 | 0.000 | 0.000 | 0.000 | 0.000 | 0.000 | 0.000 | 0.003 |
| p__Proteobacteria;c__Gammaproteobacteria;o__Gammaproteobacteria_Incertae_Sedis;f__Unknown_Family;g__Wenzhouxiangella | 0.033 | 0.024 | 0.000 | 0.000 | 0.000 | 0.000 | 0.017 | 0.000 | 0.000 | 0.000 | 0.000 | 0.019 | 0.000 | 0.004 | 0.009 | 0.000 | 0.000 | 0.000 |
| p__Proteobacteria;c__Gammaproteobacteria;o__Methylococcales;f__Methylococcaceae;g__uncultured | 0.000 | 0.000 | 0.000 | 0.000 | 0.000 | 0.000 | 0.000 | 0.000 | 0.000 | 0.000 | 0.000 | 0.000 | 0.000 | 0.004 | 0.000 | 0.000 | 0.000 | 0.003 |
| p__Proteobacteria;c__Gammaproteobacteria;o__Nitrosococcales;f__Methylophagaceae;g__Methylophaga | 0.000 | 0.000 | 0.005 | 0.010 | 0.000 | 0.000 | 0.000 | 0.000 | 0.000 | 0.000 | 0.000 | 0.000 | 0.000 | 0.000 | 0.000 | 0.000 | 0.000 | 0.000 |
| p__Proteobacteria;c__Gammaproteobacteria;o__Nitrosococcales;f__Nitrosococcaceae;g__AqS1 | 0.000 | 0.009 | 0.000 | 0.000 | 0.000 | 0.000 | 0.007 | 0.000 | 0.000 | 0.000 | 0.000 | 0.000 | 0.000 | 0.000 | 0.000 | 0.000 | 0.000 | 0.000 |
| p__Proteobacteria;c__Gammaproteobacteria;o__Oceanospirillales;f__Halomonadaceae;g__Halomonas | 0.000 | 0.000 | 0.000 | 0.000 | 0.000 | 0.000 | 0.000 | 0.000 | 0.000 | 0.000 | 0.000 | 0.004 | 0.003 | 0.000 | 0.000 | 0.000 | 0.000 | 0.000 |
| p__Proteobacteria;c__Gammaproteobacteria;o__Oceanospirillales;f__Marinomonadaceae;g__Marinomonas | 0.000 | 0.000 | 0.000 | 0.000 | 0.000 | 0.000 | 0.000 | 0.039 | 0.000 | 0.054 | 0.000 | 0.000 | 0.000 | 0.000 | 0.000 | 0.000 | 0.000 | 0.000 |
| p__Proteobacteria;c__Gammaproteobacteria;o__Oceanospirillales;f__Saccharospirillaceae;g__Reinekea | 0.000 | 0.000 | 0.000 | 0.000 | 0.000 | 0.000 | 0.000 | 0.043 | 0.000 | 0.042 | 0.000 | 0.000 | 0.000 | 0.000 | 0.000 | 0.000 | 0.000 | 0.000 |
| p__Proteobacteria;c__Gammaproteobacteria;o__Pseudomonadales;f__Moraxellaceae;g__Acinetobacter | 0.059 | 0.062 | 0.060 | 0.081 | 0.093 | 0.053 | 0.014 | 0.035 | 0.030 | 0.040 | 0.054 | 0.012 | 0.000 | 0.022 | 0.035 | 0.000 | 0.036 | 0.027 |
| p__Proteobacteria;c__Gammaproteobacteria;o__Pseudomonadales;f__Moraxellaceae;g__Psychrobacter | 0.024 | 0.019 | 0.016 | 0.017 | 0.019 | 0.015 | 0.000 | 0.000 | 0.006 | 0.015 | 0.008 | 0.000 | 0.000 | 0.000 | 0.000 | 0.000 | 0.000 | 0.000 |
| p__Proteobacteria;c__Gammaproteobacteria;o__Pseudomonadales;f__Pseudomonadaceae;g__Pseudomonas | 0.000 | 0.000 | 0.000 | 0.000 | 0.017 | 0.011 | 0.017 | 0.014 | 0.010 | 0.023 | 0.000 | 0.000 | 0.000 | 0.000 | 0.000 | 0.000 | 0.000 | 0.000 |
| p__Proteobacteria;c__Gammaproteobacteria;o__Vibrionales;f__Vibrionaceae | 0.051 | 0.038 | 0.000 | 0.000 | 0.000 | 0.117 | 0.000 | 0.489 | 0.030 | 0.920 | 0.050 | 0.031 | 0.021 | 0.013 | 0.023 | 0.000 | 0.000 | 0.016 |
| p__Proteobacteria;c__Gammaproteobacteria;o__Vibrionales;f__Vibrionaceae;g__Vibrio | 0.035 | 0.016 | 0.063 | 0.015 | 0.045 | 0.000 | 0.009 | 0.170 | 0.000 | 0.210 | 0.000 | 0.016 | 0.000 | 0.004 | 0.000 | 0.032 | 0.000 | 0.000 |
| p__Proteobacteria;c__Gammaproteobacteria;o__Xanthomonadales;f__Xanthomonadaceae | 0.086 | 0.076 | 0.052 | 0.039 | 0.030 | 0.047 | 0.017 | 0.025 | 0.027 | 0.054 | 0.056 | 0.016 | 0.000 | 0.000 | 0.000 | 0.000 | 0.000 | 0.000 |
| p__Proteobacteria;c__Gammaproteobacteria;o__Xanthomonadales;f__Xanthomonadaceae;g__Arenimonas | 0.026 | 0.000 | 0.015 | 0.010 | 0.013 | 0.000 | 0.007 | 0.000 | 0.010 | 0.020 | 0.023 | 0.005 | 0.000 | 0.000 | 0.000 | 0.000 | 0.000 | 0.000 |
| p__Proteobacteria;c__Gammaproteobacteria;o__Xanthomonadales;f__Xanthomonadaceae;g__Luteimonas | 0.018 | 0.022 | 0.023 | 0.039 | 0.022 | 0.017 | 0.000 | 0.000 | 0.000 | 0.000 | 0.023 | 0.000 | 0.000 | 0.000 | 0.000 | 0.000 | 0.000 | 0.000 |
| p__Proteobacteria;c__Gammaproteobacteria;o__Xanthomonadales;f__Xanthomonadaceae;g__Lysobacter | 0.063 | 0.033 | 0.054 | 0.066 | 0.048 | 0.024 | 0.045 | 0.032 | 0.037 | 0.035 | 0.049 | 0.029 | 0.000 | 0.000 | 0.000 | 0.000 | 0.000 | 0.000 |
| p__Proteobacteria;c__Gammaproteobacteria;o__Xanthomonadales;f__Xanthomonadaceae;g__Stenotrophomonas | 0.000 | 0.000 | 0.005 | 0.000 | 0.000 | 0.000 | 0.000 | 0.000 | 0.000 | 0.000 | 0.000 | 0.011 | 0.000 | 0.012 | 0.000 | 0.000 | 0.000 | 0.000 |
| p__Verrucomicrobiota;c__Verrucomicrobiae;o__Verrucomicrobiales | 0.000 | 0.000 | 0.000 | 0.000 | 0.000 | 0.000 | 0.000 | 0.018 | 0.000 | 0.000 | 0.005 | 0.000 | 0.000 | 0.000 | 0.000 | 0.000 | 0.000 | 0.000 |
| p__Verrucomicrobiota;c__Verrucomicrobiae;o__Verrucomicrobiales;f__Akkermansiaceae;g__Akkermansia | 0.055 | 0.066 | 1.161 | 0.077 | 0.035 | 0.064 | 4.214 | 0.436 | 0.050 | 1.727 | 0.393 | 0.145 | 0.334 | 18.450 | 33.630 | 0.116 | 0.093 | 0.105 |
| p__Verrucomicrobiota;c__Verrucomicrobiae;o__Verrucomicrobiales;f__Rubritaleaceae;g__Haloferula | 0.020 | 0.007 | 0.010 | 0.004 | 0.009 | 0.009 | 0.009 | 0.000 | 0.000 | 0.005 | 0.012 | 0.008 | 0.004 | 0.006 | 0.000 | 0.000 | 0.000 | 0.004 |
| p__Verrucomicrobiota;c__Verrucomicrobiae;o__Verrucomicrobiales;f__Rubritaleaceae;g__Luteolibacter | 0.000 | 0.000 | 0.000 | 0.000 | 0.002 | 0.006 | 0.000 | 0.002 | 0.018 | 0.000 | 0.000 | 0.008 | 0.000 | 0.000 | 0.000 | 0.000 | 0.000 | 0.000 |
| p__Verrucomicrobiota;c__Verrucomicrobiae;o__Verrucomicrobiales;f__Rubritaleaceae;g__Rubritalea | 0.020 | 0.019 | 0.013 | 0.019 | 0.017 | 0.015 | 0.000 | 0.000 | 0.000 | 0.020 | 0.018 | 0.000 | 0.000 | 0.000 | 0.000 | 0.000 | 0.000 | 0.000 |
| p__WPS-2;c__WPS-2;o__WPS-2;f__WPS-2;g__WPS-2 | 0.000 | 0.000 | 0.000 | 0.000 | 0.000 | 0.000 | 0.000 | 0.005 | 0.000 | 0.000 | 0.000 | 0.000 | 0.004 | 0.000 | 0.000 | 0.000 | 0.000 | 0.000 |
| Total | 100 | 100 | 100 | 100 | 100 | 100 | 100 | 100 | 100 | 100 | 100 | 100 | 100 | 100 | 100 | 100 | 100 | 100 |

**Table S4** the MetaCyc pathways relative abundance and significant difference of HFD+ZD compared with control (Cl) and HFD groups

|  |  |  |  | p-values | p-values | p-values |
| --- | --- | --- | --- | --- | --- | --- |
| description | CL(%) | HFD(%) | HFD+ZD(%) | CL vs HFD | CL vs HFD+ZD | HFD+ZDvsHFD |
| sucrose degradation IV (sucrose phosphorylase) | 0.04 | 0.23 | 0.06 | 0.001 | 0.492 | 0.003 |
| UDP-N-acetyl-D-glucosamine biosynthesis I | 0.58 | 0.63 | 0.57 | 0.029 | 0.597 | 0.017 |
| superpathway of (Kdo)2-lipid A biosynthesis | 0.02 | 0.05 | 0.00 | 0.002 | 0.002 | 0.002 |
| enterobacterial common antigen biosynthesis | 0.00 | 0.01 | 0.00 | 0.005 | 0.002 | 0.005 |
| polymyxin resistance | 0.00 | 0.02 | 0.00 | 0.010 | 0.009 | 0.004 |
| methylphosphonate degradation I | 0.01 | 0.01 | 0.00 | 0.016 | 0.001 | 0.003 |
| superpathway of chorismate metabolism | 0.03 | 0.06 | 0.02 | 0.020 | 0.000 | 0.003 |
| superpathway of L-tryptophan biosynthesis | 0.01 | 0.07 | 0.00 | 0.028 | 0.002 | 0.019 |
| glyoxylate cycle | 0.02 | 0.04 | 0.00 | 0.009 | 0.002 | 0.002 |
| fatty acid salvage | 0.02 | 0.04 | 0.01 | 0.029 | 0.005 | 0.001 |
| superpathway of L-arginine and L-ornithine degradation | 0.00 | 0.01 | 0.00 | 0.038 | 0.000 | 0.023 |
| superpathway of L-arginine, putrescine, and 4-aminobutanoate degradation | 0.00 | 0.01 | 0.00 | 0.038 | 0.000 | 0.023 |
| superpathway of glycolysis, pyruvate dehydrogenase, TCA, and glyoxylate bypass | 0.05 | 0.08 | 0.01 | 0.046 | 0.000 | 0.002 |
| superpathway of glyoxylate bypass and TCA | 0.03 | 0.04 | 0.00 | 0.049 | 0.000 | 0.002 |
| enterobactin biosynthesis | 0.01 | 0.04 | 0.00 | 0.002 | 0.002 | 0.002 |
| palmitate biosynthesis II (bacteria and plants) | 0.08 | 0.09 | 0.03 | 0.877 | 0.001 | 0.020 |
| superpathway of fatty acid biosynthesis initiation (E. coli) | 0.04 | 0.05 | 0.02 | 0.768 | 0.001 | 0.027 |
| peptidoglycan maturation (meso-diaminopimelate containing) | 0.58 | 0.75 | 0.30 | 0.093 | 0.000 | 0.002 |
| glutaryl-CoA degradation | 0.04 | 0.05 | 0.02 | 0.122 | 0.000 | 0.003 |
| fatty acid &beta;-oxidation I | 0.04 | 0.05 | 0.01 | 0.156 | 0.000 | 0.003 |
| sucrose degradation III (sucrose invertase) | 0.69 | 0.88 | 0.64 | 0.074 | 0.389 | 0.029 |
| superpathway of fucose and rhamnose degradation | 0.06 | 0.08 | 0.05 | 0.122 | 0.827 | 0.038 |
| NAD biosynthesis I (from aspartate) | 0.51 | 0.57 | 0.50 | 0.187 | 0.675 | 0.050 |
| superpathway of Clostridium acetobutylicum acidogenic fermentation | 0.09 | 0.12 | 0.04 | 0.061 | 0.000 | 0.000 |
| pyruvate fermentation to butanoate | 0.07 | 0.10 | 0.03 | 0.063 | 0.000 | 0.001 |
| peptidoglycan biosynthesis IV (Enterococcus faecium) | 0.46 | 0.50 | 0.21 | 0.647 | 0.002 | 0.004 |
| superpathway of glycol metabolism and degradation | 0.01 | 0.02 | 0.00 | 0.104 | 0.000 | 0.010 |
| ppGpp biosynthesis | 0.03 | 0.04 | 0.01 | 0.255 | 0.000 | 0.004 |
| formaldehyde oxidation I | 0.06 | 0.08 | 0.13 | 0.271 | 0.003 | 0.031 |
| aerobic respiration I (cytochrome c) | 0.10 | 0.13 | 0.03 | 0.308 | 0.002 | 0.012 |
| TCA cycle IV (2-oxoglutarate decarboxylase) | 0.07 | 0.08 | 0.01 | 0.444 | 0.000 | 0.003 |
| TCA cycle VII (acetate-producers) | 0.03 | 0.04 | 0.01 | 0.477 | 0.002 | 0.021 |
| dTDP-L-rhamnose biosynthesis I | 0.75 | 0.77 | 0.83 | 0.584 | 0.013 | 0.018 |
| (5Z)-dodec-5-enoate biosynthesis | 0.05 | 0.06 | 0.02 | 0.769 | 0.001 | 0.026 |
| palmitoleate biosynthesis I (from (5Z)-dodec-5-enoate) | 0.05 | 0.06 | 0.02 | 0.771 | 0.001 | 0.026 |
| mycolate biosynthesis | 0.06 | 0.07 | 0.03 | 0.772 | 0.001 | 0.026 |
| oleate biosynthesis IV (anaerobic) | 0.06 | 0.07 | 0.03 | 0.776 | 0.001 | 0.025 |
| stearate biosynthesis II (bacteria and plants) | 0.05 | 0.06 | 0.02 | 0.795 | 0.001 | 0.027 |
| superpathway of pyrimidine nucleobases salvage | 0.96 | 0.97 | 0.91 | 0.939 | 0.004 | 0.048 |
| aspartate superpathway | 0.32 | 0.33 | 0.17 | 0.950 | 0.010 | 0.021 |
| flavin biosynthesis I (bacteria and plants) | 0.58 | 0.43 | 0.61 | 0.026 | 0.318 | 0.002 |
| 4-deoxy-L-threo-hex-4-enopyranuronate degradation | 0.25 | 0.13 | 0.24 | 0.001 | 0.725 | 0.003 |
| L-histidine degradation I | 0.15 | 0.06 | 0.10 | 0.001 | 0.027 | 0.007 |
| GDP-mannose biosynthesis | 0.61 | 0.44 | 0.59 | 0.004 | 0.520 | 0.007 |
| ADP-L-glycero-&beta;-D-manno-heptose biosynthesis | 0.11 | 0.06 | 0.11 | 0.004 | 0.992 | 0.040 |
| pantothenate and coenzyme A biosynthesis I | 0.50 | 0.39 | 0.46 | 0.009 | 0.132 | 0.065 |
| superpathway of GDP-mannose-derived O-antigen building blocks biosynthesis | 0.44 | 0.30 | 0.42 | 0.027 | 0.531 | 0.045 |
| urea cycle | 0.08 | 0.04 | 0.09 | 0.047 | 0.552 | 0.007 |
| L-arginine biosynthesis III (via N-acetyl-L-citrulline) | 0.29 | 0.11 | 0.17 | 0.000 | 0.002 | 0.024 |
| acetyl-CoA fermentation to butanoate II | 0.15 | 0.07 | 0.21 | 0.015 | 0.015 | 0.002 |
| biotin biosynthesis II | 0.05 | 0.02 | 0.09 | 0.026 | 0.087 | 0.002 |
| urate biosynthesis/inosine 5'-phosphate degradation | 0.65 | 0.52 | 0.68 | 0.089 | 0.359 | 0.041 |
| colanic acid building blocks biosynthesis | 0.45 | 0.35 | 0.48 | 0.093 | 0.211 | 0.041 |
| superpathway of ubiquinol-8 biosynthesis (prokaryotic) | 0.06 | 0.05 | 0.07 | 0.460 | 0.314 | 0.016 |
| L-tryptophan biosynthesis | 0.57 | 0.53 | 0.68 | 0.475 | 0.009 | 0.020 |
| ubiquinol-10 biosynthesis (prokaryotic) | 0.06 | 0.05 | 0.08 | 0.500 | 0.275 | 0.016 |
| ubiquinol-7 biosynthesis (prokaryotic) | 0.06 | 0.05 | 0.08 | 0.500 | 0.275 | 0.016 |
| ubiquinol-8 biosynthesis (prokaryotic) | 0.06 | 0.05 | 0.08 | 0.500 | 0.275 | 0.016 |
| ubiquinol-9 biosynthesis (prokaryotic) | 0.06 | 0.05 | 0.08 | 0.500 | 0.275 | 0.016 |
| pyruvate fermentation to acetone | 0.18 | 0.16 | 0.26 | 0.567 | 0.034 | 0.013 |
| photorespiration | 0.13 | 0.12 | 0.26 | 0.685 | 0.010 | 0.008 |
| 2-methylcitrate cycle II | 0.013 | 0.012 | 0.00 | 0.907 | 0.000 | 0.013 |
| D-galacturonate degradation I | 0.33 | 0.34 | 0.48 | 0.577 | 0.006 | 0.009 |
| pyruvate fermentation to isobutanol (engineered) | 0.98 | 1.17 | 1.36 | 0.000 | 0.000 | 0.014 |
| purine nucleobases degradation I (anaerobic) | 0.20 | 0.38 | 0.64 | 0.000 | 0.000 | 0.005 |
| D-glucarate degradation I | 0.03 | 0.10 | 0.21 | 0.002 | 0.000 | 0.000 |
| L-isoleucine biosynthesis II | 0.98 | 1.11 | 1.21 | 0.009 | 0.001 | 0.048 |
| superpathway of D-glucarate and D-galactarate degradation | 0.01 | 0.04 | 0.10 | 0.006 | 0.000 | 0.001 |
| allantoin degradation to glyoxylate III | 0.01 | 0.04 | 0.10 | 0.003 | 0.001 | 0.003 |
| guanosine nucleotides degradation III | 0.25 | 0.33 | 0.57 | 0.078 | 0.000 | 0.000 |
| formaldehyde assimilation II (RuMP Cycle) | 0.08 | 0.15 | 0.30 | 0.117 | 0.000 | 0.005 |
| superpathway of glucose and xylose degradation | 0.13 | 0.17 | 0.26 | 0.129 | 0.001 | 0.014 |
| GDP-D-glycero-&alpha;-D-manno-heptose biosynthesis | 0.13 | 0.16 | 0.34 | 0.129 | 0.001 | 0.003 |
| purine nucleotides degradation II (aerobic) | 0.27 | 0.33 | 0.42 | 0.140 | 0.004 | 0.038 |
| adenosine nucleotides degradation II | 0.23 | 0.30 | 0.57 | 0.143 | 0.000 | 0.000 |
| D-galactarate degradation I | 0.01 | 0.04 | 0.10 | 0.006 | 0.000 | 0.001 |
| myo-, chiro- and scillo-inositol degradation | 0.06 | 0.06 | 0.14 | 0.888 | 0.004 | 0.005 |
